# DrSVision: A Machine Learning Tool for Cortical Region-Specific fNIRS Calibration Based on Cadaveric Head MRI

**DOI:** 10.3390/s25206340

**Published:** 2025-10-14

**Authors:** Serhat Ilgaz Yöner, Mehmet Emin Aksoy, Hayrettin Can Südor, Kurtuluş İzzetoğlu, Baran Bozkurt, Alp Dinçer

**Affiliations:** 1 Department of Biomedical Equipment Technology, Junior College, Acıbadem Mehmet Ali Aydınlar University, Istanbul 34752, Türkiye; emin.aksoy@acibadem.edu.tr (M.E.A.);; 2 CASE (Center of Advanced Simulation and Education), Acıbadem Mehmet Ali Aydınlar University, Istanbul 34752, Türkiye; 3 School of Biomedical Engineering, Science and Health Systems, Drexel University, Philadelphia, PA 19104, USA; 4 Department of Neurosurgery, School of Medicine, Acıbadem Mehmet Ali Aydınlar University, Istanbul 34752, Türkiye; 5 Neuroanatomy Laboratory, School of Medicine, Acıbadem Mehmet Ali Aydınlar University, Istanbul 34752, Türkiye; 6 Center for Neuroradiological Applications and Research, Acıbadem Mehmet Ali Aydınlar University, Istanbul 34752, Türkiye; 7 Department of Radiology, School of Medicine, Acıbadem Mehmet Ali Aydınlar University, Istanbul 34752, Türkiye

**Keywords:** functional near-infrared spectroscopy, fNIRS, source-detector separation, sensitivity at depth, Monte Carlo simulations, machine learning, gaussian process regression, magnetic resonance imaging, MRI, calibration tool

## Abstract

Functional Near-Infrared Spectroscopy is (fNIRS) a non-invasive neuroimaging technique that monitors cerebral hemodynamic responses by measuring near-infrared (NIR) light absorption caused by changes in oxygenated and deoxygenated hemoglobin concentrations. While fNIRS has been widely used in cognitive and clinical neuroscience, a key challenge persists: the lack of practical tools required for calibrating source-detector separation (SDS) to maximize sensitivity at depth (SAD) for monitoring specific cortical regions of interest to neuroscience and neuroimaging studies. This study presents DrSVision version 1.0, a standalone software developed to address this limitation. Monte Carlo (MC) simulations were performed using segmented magnetic resonance imaging (MRI) data from eight cadaveric heads to realistically model light attenuation across anatomical layers. SAD of 10–20 mm with SDS of 19–39 mm was computed. The dataset was used to train a Gaussian Process Regression (GPR)-based machine learning (ML) model that recommends optimal SDS for achieving maximal sensitivity at targeted depths. The software operates independently of any third-party platforms and provides users with region-specific calibration outputs tailored for experimental goals, supporting more precise application of fNIRS. Future developments aim to incorporate subject-specific calibration using anatomical data and broaden support for diverse and personalized experimental setups. DrSVision represents a step forward in fNIRS experimentation.

## 1. Introduction

fNIRS is a non-invasive neuroimaging technique that measures brain activity by detecting changes in cerebral blood oxygenation. fNIRS systems transmit NIR light through tissue layers the scalp-muscle, cranium, cerebrospinal fluid (CSF), and brain, and capture the light that is scattered back, allowing for the estimation of oxygenated and deoxygenated hemoglobin concentrations in the underlying cortical tissue. Since these hemodynamic changes are tightly coupled with neuronal activity, fNIRS provides an indirect yet reliable measure of brain function [1,2]. Its portability, affordability and motion tolerance make it especially suitable for populations and environments where traditional neuroimaging methods, such as functional magnetic resonance imaging (MRI) or positron emission tomography, are impractical, such as infants, individuals with movement disorders, or real-world behavioral experiments [3,4]. Furthermore, its safety, comfort, and scalability support repeated lengthy measurement sessions. These features position fNIRS as a highly promising tool for cognitive neuroscience, neurorehabilitation, psychiatry, and brain–computer interface applications [5]. Its growing use in occupational training, such as surgery and aviation, highlights fNIRS’s potential to objectively assess real time learning and decision-making in high-risk environments [6,7,8].

**Figure 1:**
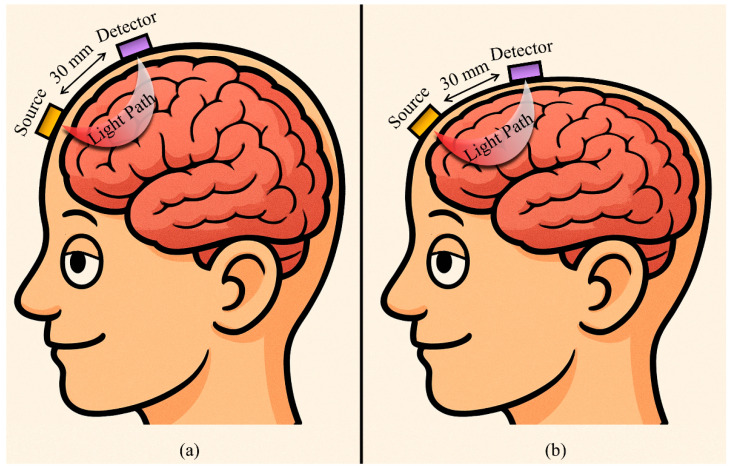
Illustrations of the fixed SDS problem in fNIRS; identical 30 mm SDS on both (**a**) a curved head, and (**b**) a flatter head leads to vastly different light paths, affecting SAD and spatial specificity.

However, one significant limitation of fNIRS is the absence of region-specific calibration for cortical probes, which can impact spatial accuracy. The accuracy and SAD (also known as depth sensitivity) of fNIRS signals are highly dependent on SDS, which determines how deep the NIR light can penetrate and from which cortical regions the signal originates [9]. Standard fNIRS probe designs typically employ fixed SDS, applied uniformly across subjects and tasks, without accounting for individual differences in head geometry or the depth of the targeted cortical region. This generalized approach risks reducing measurement precision, especially in studies aiming to localize activity at specific prefrontal cortex (PFC) subregions, like the dorsolateral PFC, ventromedial PFC, and frontopolar cortex (see Figure 1). Although this is the widespread practice, the problem of varying spatial accuracy is rarely examined or justified in the literature, despite these potential implications.

Some recent studies have attempted to improve anatomical specificity by aligning source-detector positions with standardized head models or using MRI-guided probe placement [10,11,12]. While these methods enhance spatial alignment, they often fall short of offering quantitative strategies for adjusting SDS to optimize the SAD of interest. To advance fNIRS as a study-oriented neuroimaging modality, calibration strategies must be tailored to both anatomical and experimental contexts. Region-specific optimization of SDS is therefore essential to ensure that measurements are anatomically relevant and spatially accurate, particularly when targeting regions located at varying cortical depths [13].

Some of these studies incorporate photon transport modeling to estimate the sensitivity profile of specific probe configurations and determine how effectively light reaches target cortical regions; among these, MC simulations have become the most widely adopted and validated approach [14,15]. In the context of fNIRS, MC methods are frequently used to simulate NIR light propagation through layered head structures, enabling researchers to quantify photon fluence distributions, absorption patterns, and SAD. Their probabilistic nature allows for the detailed modeling of scattering and absorption events, making them particularly suitable for evaluating how different SDS influence signal quality and spatial specificity. As such, MC simulations have become a cornerstone in bio-photonics research, supporting both theoretical and experimental efforts to optimize fNIRS measurements in anatomically realistic scenarios [16].

Despite their accuracy and detail, MC simulations are computationally intensive and time-consuming [17,18], limiting their practical use in rapid probe design and calibration. To address these limitations, recent advances in fNIRS research increasingly leverage ML techniques to enhance both data interpretation and system design. While much of the existing literature focuses on classifying clinical conditions using fNIRS-derived connectivity patterns [19], regression-based ML approaches have also been employed to efficiently generalize outcomes from computationally intensive MC simulations [20]. Once trained, these models can rapidly estimate sensitivity metrics such as SDS and SAD based on probe geometry and anatomical features. This capability enables faster geometry-focused probe design assessments that reduce reliance on repeated full-scale simulations [21]. To date, fully region-specific calibration, optimizing probe parameters explicitly for targeted cortical regions aligned with neuroscientific goals remains largely unexplored and represents a promising direction for future work.

This study implements a novel approach and uses MRI scans of cadaveric heads to model photon transport in eight different human heads within a MC simulation environment. The simulation outputs are then integrated with ML predictions to develop a neuroscientific study-oriented calibration tool, DrSVision. The following sections detail the materials and methods: Magnetic Resonance Imaging, Simulations, Post-Processing, Machine Learning Predictions, and DrSVision Software.

## 2. Materials and Methods

**Figure 2:**
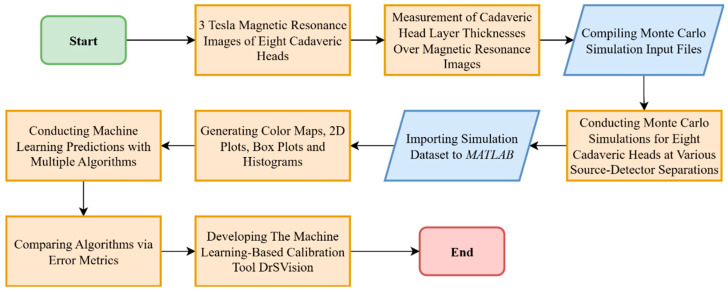
Overview of the methodological workflow in this study using flow chart symbols.

The methodological workflow begins with MRI scans of cadaveric heads, followed by the measurement of head layer thicknesses, MC simulations, post-processing of simulation outputs, ML modeling and evaluation, and the development of the ML-based calibration tool, DrSVision. A summary of the full workflow is presented in the flowchart in Figure 2.

### 2.1. Magnetic Resonance Imaging

The cadaveric human heads used in this study were obtained through a licensed anatomical supplier, with all donors having provided informed consent during their lifetime for the use of their remains in scientific research and education. The study protocol was approved by the institutional ethics committee, and all procedures were conducted in accordance with relevant ethical and legal guidelines governing the use of human cadaveric material. A total of eight adult cadaveric heads, five male and three female, with ages between 67 and 97 years were selected based on the absence of cranial trauma, prior surgical intervention, or visible pathology. All cadaveric heads were initially perfused with formalin to fix internal structures, then preserved via fresh-freezing [22]. Prior to scanning, the heads were thawed at room temperature for a minimum of 24 h to minimize imaging artifacts related to tissue freezing. This combined fixation and freezing protocol aimed to retain anatomical detail while preserving imaging contrast. During handling and imaging, standard biosafety and anatomical research protocols were strictly followed.

Each of the eight cadaveric heads was scanned individually using MAGNETOM Prisma Fit model (Siemens Healthineers, Erlangen, Germany) 3 Tesla MRI to acquire high-resolution anatomical data for subsequent analysis. Imaging was performed using a standard head coil to maximize signal-to-noise ratio (SNR) and spatial resolution (Voxel size: 0.80 × 0.80 × 0.90 mm^3^). The cadaveric heads were carefully positioned in the head coil to ensure consistent alignment and minimize movement related artifacts. T1 and T2 weighted sequences were acquired in axial, sagittal, and coronal planes to capture comprehensive soft tissue and structural contrast. Imaging parameters for the T1 weighted sequence included a repetition time (TR) of approximately 1960 ms and an echo time (TE) of 3.2 ms, while the T2 weighted sequence used a TR of approximately 3000 ms and a TE of around 408 ms. Field of view, matrix size, and slice thickness were standardized across all scans to allow for reliable inter-cadaveric comparisons. Each head scan required approximately 16 min in total. No contrast agents were used during scanning.

### 2.2. Simulations

NIR light transport in eight cadaveric heads was simulated using Monte Carlo eXtreme (MCX) v2025, an open-source MC-based software specifically developed for simulating photon transport in 3D turbid media by tracking photon packets through scattering and absorption events probabilistically [14]. Each photon deposits part of its energy as it propagates, and the cumulative energy deposition at each voxel represents the photon fluence Φ. Fluence $\Phi \left(x,y,z\right)$ at voxel coordinates $\left(x,y,z\right)$ is computed as the sum of weights of all photons passing through that voxel,

(1) $$\Phi \left(x,y,z\right)=\sum_{i=1}^{N_{x,y,z}}w_{i}$$

where $N_{x,y,z}$ is the number of photons passing through voxel $\left(x,y,z\right)$. $w_{i}$ is the weight of the i-th photon in that voxel. This discrete summation reflects the statistical accumulation of photon energy and forms the basis for analyzing light propagation and absorption in tissue models. A 3D domain consisting of 64 voxels along each axis (64 × 64 × 64), with each voxel measuring 1 mm^3^ to achieve a balance between resolution and computational cost, was structured to represent the scalp-muscle, cranium, CSF, and brain layers of the head. Planar slab geometries were used to model each tissue layer within this voxelated domain. Non-brain-tissue layer thicknesses for each cadaveric head were determined from MRI data, and the remaining domain was assigned as brain tissue.

The optical properties of each layer including absorption coefficient ($\mu_{a}$), scattering coefficient ($\mu_{s}$), anisotropy factor (g), and refractive index (n) were determined based on values reported in the scientific literature [23] (see Table 1).

To closely replicate the optical characteristics of the light-emitting diode (LED) used in conventional fNIRS systems, the light source type was set to ‘cone’ with a half-angle of 70°. The source and the detector were vertically centered and positioned directly above the scalp-muscle layer, perpendicular to the surface. Initially, the source and the detector were placed at horizontal coordinates of 29.5 and 34.5 mm, respectively, with their midpoint corresponding to the center of the plane. Simulations were conducted for each cadaveric head and repeated for the SDS ranging from 19 to 39 mm, with a step size of 2 mm. To obtain stable noise-free fluence distributions, each simulation used 10^9^ photons. This high photon count ensured that any statistical fluctuations were negligible compared to the optical variations being investigated, allowing for a precise analysis of the photon transport processes [24,25]. Boundary conditions accounted for refractive index mismatches between tissue layers, including Fresnel reflections at interfaces. The default MCX parameters for wavelength, power normalization, and initial photon weight were used. All simulations were performed on a workstation running Windows 10 Pro (v22H2, Microsoft Corporation, Redmond, WA, US) equipped with an NVIDIA GeForce MX450 GPU (NVIDIA Corporation, Santa Clara, CA, US) and 8 GB of RAM. Each simulation took approximately 290 s to complete. The resulting data were saved in .jnii and .jdat formats, containing fluence maps and detected photon counts, respectively.

### 2.3. Post-Processing

In MATLAB (v24.2, MathWorks Inc., Portola Valley, CA, USA) [26], fluence data in .jnii format were decoded, decompressed, and reshaped into 3D arrays with voxel dimensions of 1 × 1 × 1 mm^3^ for each cadaveric head–SDS combination. First, fluence volumes were visualized from the top and front anatomical perspectives using a custom red–white colormap in logarithmic scale to enhance dynamic range. Then, SAD values were computed in the percentage form by summing fluence within a circular region of interest (ROI) with 10 mm radius centered at the midpoint between the source and detector positions across each 1 mm depth increment perpendicular along the tissue depth axis. A 10 mm radius was selected, as it provides a balance between spatial specificity and sufficient sampling volume, consistent with common practice in the literature. This scale has also been shown to capture relevant hemodynamic or optical changes while minimizing contamination from the surrounding regions [27,28].

(2) $$SAD\left(z\right)=\frac{\sum_{\left(x,y\right)\in R\left(z\right)}\Phi \left(x,y,z\right)}{\sum_{x,y,z}\Phi \left(x,y,z\right)}\times 100\%$$

where $R\left(z\right)$ denotes the set of voxels within the ROI at depth z. SAD versus (vs.) depth curves were plotted for all cadaveric heads at each SDS and systematically recorded in Excel sheets. For statistical comparison, SAD vs. SDS box plots were generated for each depth layer across all heads. Lastly, detected photon data in .jdat format were decoded and used to plot histograms of mean photon count vs. mean path length for each cadaveric head and SDS. The utilized post-processing workflow enabled a high-resolution spatial and statistical assessment of depth sensitivity and photon propagation characteristics, with all plots saved as compressed .tiff images at 300 dpi to ensure optimal resolution and file size management.

### 2.4. Machine Learning Predictions and DrSVision Software

The dataset was modeled using Support Vector Regression (SVR) and GPR ML algorithms in MATLAB to examine the relationship between SDS and depth (independent variables) and SAD (dependent variable). SVR is a method that uses flexible curves to fit data, aiming to stay within a certain margin of error. This helps it capture patterns without getting too caught up in noise, reducing the risk of overfitting [29]. GPR takes a more statistical approach by treating predictions as part of a broader distribution, allowing for it to not only make forecasts, but also provide confidence estimates, especially useful for small or variable datasets. SVR tends to handle larger datasets more efficiently, while GPR is valuable when understanding prediction uncertainty is important [30]. For SVR, a Gaussian (RBF) kernel was used, with hyperparameters, including KernelScale, BoxConstraint, and Epsilon selected based on standard data-driven procedures implemented in MATLAB to ensure robust performance. For GPR, a squared exponential (RBF) kernel was used, with hyperparameters determined via maximum likelihood estimation. Leave-one-out cross-validation (LOOCV) was employed to evaluate model performance, with each iteration training on all but one data point and testing on the excluded sample. Model performance was evaluated using the adjusted R^2^ metric. The model yielding the higher adjusted R^2^ score was selected for integration into the standalone application named DrSVision (Doctor Signal Vision). For final predictions, each model was trained on the full standardized dataset and used to predict values over a standardized input grid. These settings ensured reproducibility, comparability across methods, and reliable estimation of sensitivity.

The application was designed in MATLAB App Designer to operate in two modes: estimation of SDS based on user-defined cortical depth inputs between 10 and 20 mm, or estimation of cortical depth based on SDS inputs between 19 and 39 mm. Mode selection is conducted through a “radio button” interface, and numerical input within the allowed range is adjusted using a “rotary knob” control. In both modes, when the “Calculate” button is pressed, output values corresponding to 1, 2, 3, 4, 5, and 6% SAD are presented to the user if available for the given input. The relationship among SDS, depth, and SAD is continuously visualized via a rotatable and zoomable 3D surface plot, rendered with a turbo color map, within the application interface.

For demonstration purposes, a mock application scenario targeting dorsolateral PFC region was prepared. A mean scalp-to-cortex depth of 14.8 mm, derived from anatomical references [31], was used as the input parameter.

## 3. Results

Table 2 presents the scalp-muscle, cranium, and CSF layer thicknesses measured from MRI scans of eight different cadaveric heads, along with the mean and standard deviation (std) values across all samples. The mean thicknesses of the scalp-muscle, cranium, and CSF layers were 5.64 ± 1.4, 7.81 ± 0.9, and 5.92 ± 4.82 mm, respectively. Among the cadaveric heads, #1 exhibited the thickest scalp-muscle layer, #8 had the thickest cranium, and #3 showed the thickest CSF layer. In contrast, the thinnest scalp-muscle, cranium, and CSF layers were observed in #6, #1, and #8, respectively.

**Figure 3:**
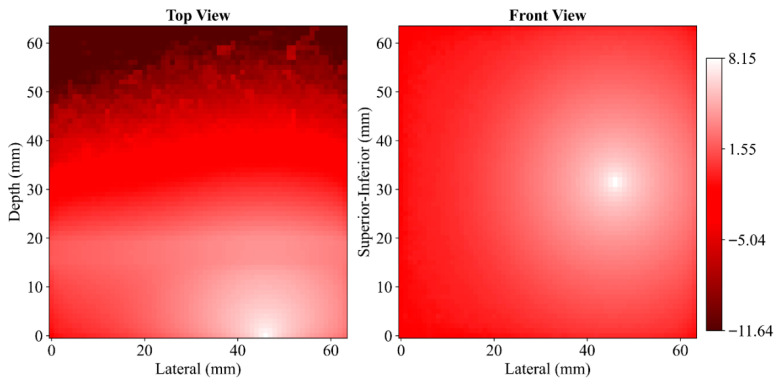
Spatial fluence color maps for cadaveric head #1 at 29 mm SDS from top view (**left**) and front view (**right**). The bright white spots indicate the position of the light source. The color bar represents photon fluence in a logarithmic scale to enhance visibility across a wide dynamic range.

Photon propagation patterns through anatomical layers were examined using 2D fluence maps generated from simulations. Figure 3 shows results for cadaveric head #1 at a SDS of 29 mm. In this simulation, the highest fluence values occur at the light source position, with a clear decline in intensity as photons spread frontally (top view), laterally and transversely (front view) through the tissue layers. The top view corresponds to the transverse plane intersecting the vertical axis of the light source, while the front view captures the frontal plane aligned with the point of contact. The spatial distribution shows a monotonic decrease in fluence with increasing distance from the source. Similar distributions were obtained across all cadaveric heads and SDSs. The full set of fluence maps is provided in the Supplementary Materials.

**Figure 4:**
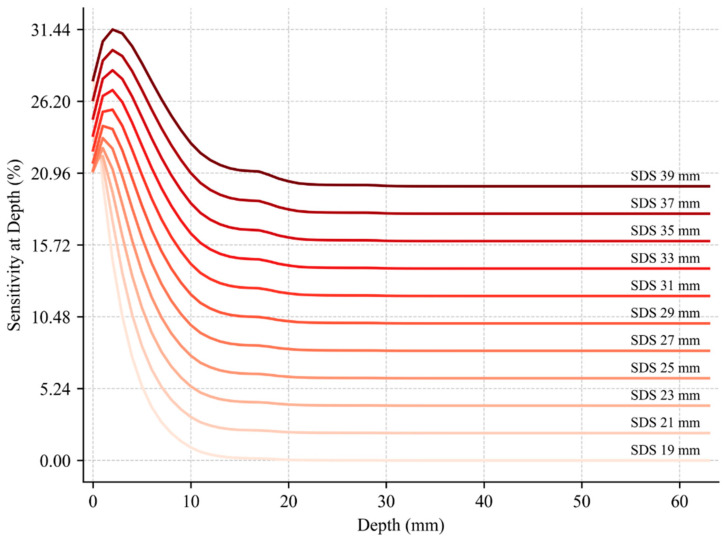
Mean SAD versus depth 2D plots for SDS values ranging from 19 to 39 mm, displayed from lightest to darkest red. Each curve represents the mean SAD computed across all cadaveric heads. To improve visual clarity and reduce overlap, each curve (except the first, SDS = 19 mm) has been vertically shifted upward in 2% increments. Readers should subtract these vertical shifts to interpret the absolute SAD values correctly.

To evaluate how SAD varies with different SDSs, mean SAD profiles were computed for SDSs ranging from 19 to 39 mm. For each SDS, mean SAD values across all cadaveric heads were computed to generate representative curves (see Figure 4). These profiles illustrate how the distribution of SAD changes with depth under varying geometric configurations. Since each cadaveric head has distinct anatomical features such as different scalp, skull, and CSF layer thicknesses, the mean profiles reflect SAD behavior across a diverse set of head geometries. Notably, the depth at which SAD reaches its maximum shifts progressively from approximately 0 to 2 mm as the SDS increases from 19 to 39 mm. This trend is consistent with established findings in the literature and reflects the expected behavior of photon migration in layered head tissue [32,33,34]. To minimize overlap and improve visual clarity, each curve was vertically shifted in 2% increments. This visualization enables the direct comparison of SAD trends across a range of SDS, independent of cadaveric head-specific variations. For each SDS, the corresponding plot showing SAD vs. depth curves for all eight cadaveric heads is provided in the Supplementary Materials to illustrate inter-subject variability and support further comparison across individual anatomical structures.

**Figure 5:**
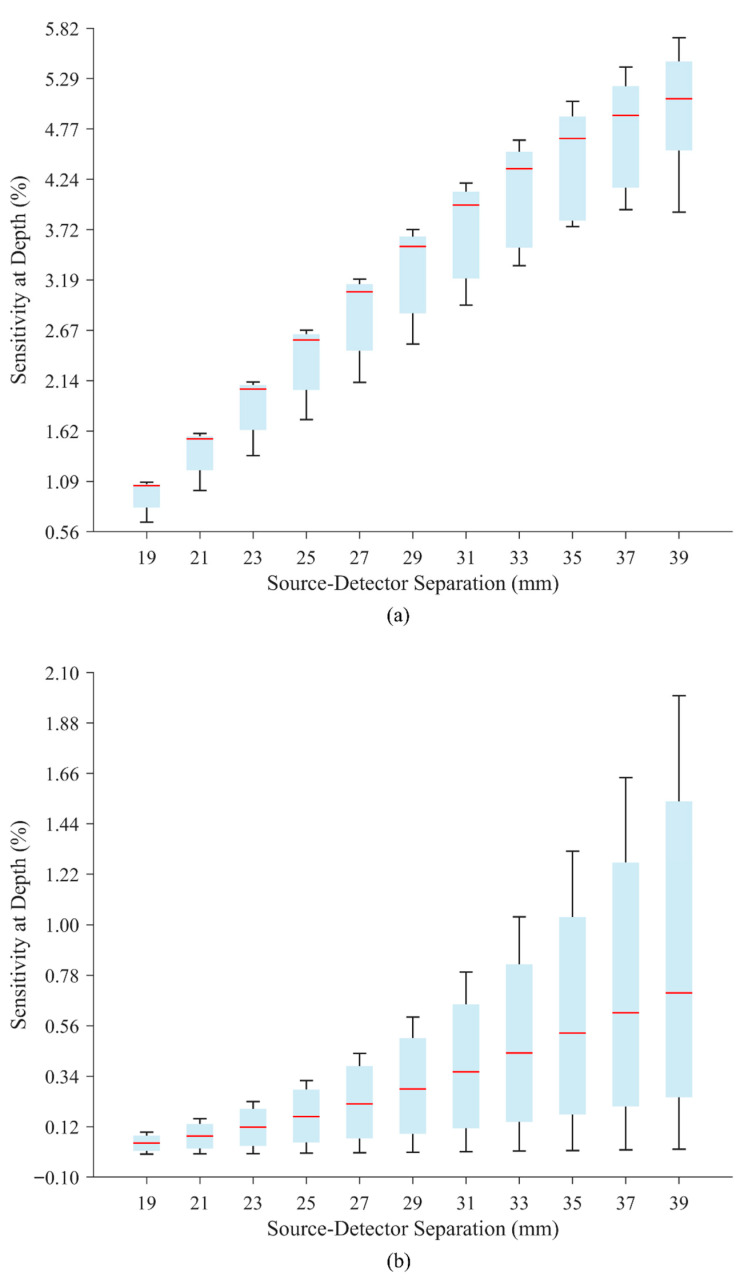
SAD vs. SDS box plots at (**a**) 10 (**b**) and 20 mm depths (each box plot is generated over all cadaveric heads).

Figure 5 shows the generated box plots for all SDSs at 10 and 20 mm depths, which represent the boundary values of the simulated depth range. These plots illustrate the distribution of SAD values across the eight cadaveric heads. Each box displays the median (red line) and interquartile range (blue region). At 10 mm depth, SAD values increased with larger SDSs, and the median values were consistently positioned closer to the upper quartile. At 20 mm depth, a similar trend was observed with generally lower SAD values, and the medians were more centrally located within the interquartile range. The variation in box heights reflects the inter-subject differences in the dataset. Corresponding box plots for all intermediate depths from 10 to 20 mm in 1 mm increments are provided in the Supplementary Materials to enable a more detailed inspection of depth-dependent variability; outliers (red cross) appear in some of these additional plots, although not in the representative plots shown in Figure 5.

Table 3 summarizes the total number of photons detected by the detector for each cadaveric head across SDSs ranging between 19 and 39 mm, as obtained from MC simulations under identical input conditions. For all cadaveric heads, a consistent decrease in detected photon count was observed with increasing SDS, as expected. Inter-cadaver variability was present at each SDS. Detector in #3 yielded the lowest number of detected photons across all SDSs, while detector in #5 consistently exhibited the highest counts within the 19–33 mm range. However, in simulations conducted beyond 35 mm SDS, detector in #5 no longer detected the highest photon counts among the dataset. The results show separation-dependent changes in the detected photon counts alongside inter-cadaver variability. Additionally, photon count vs. path length histograms were generated for each SDS and are provided in the Supplementary Materials, as these detailed analyses complement the main results without being central to the primary focus of this study.

**Figure 6:**
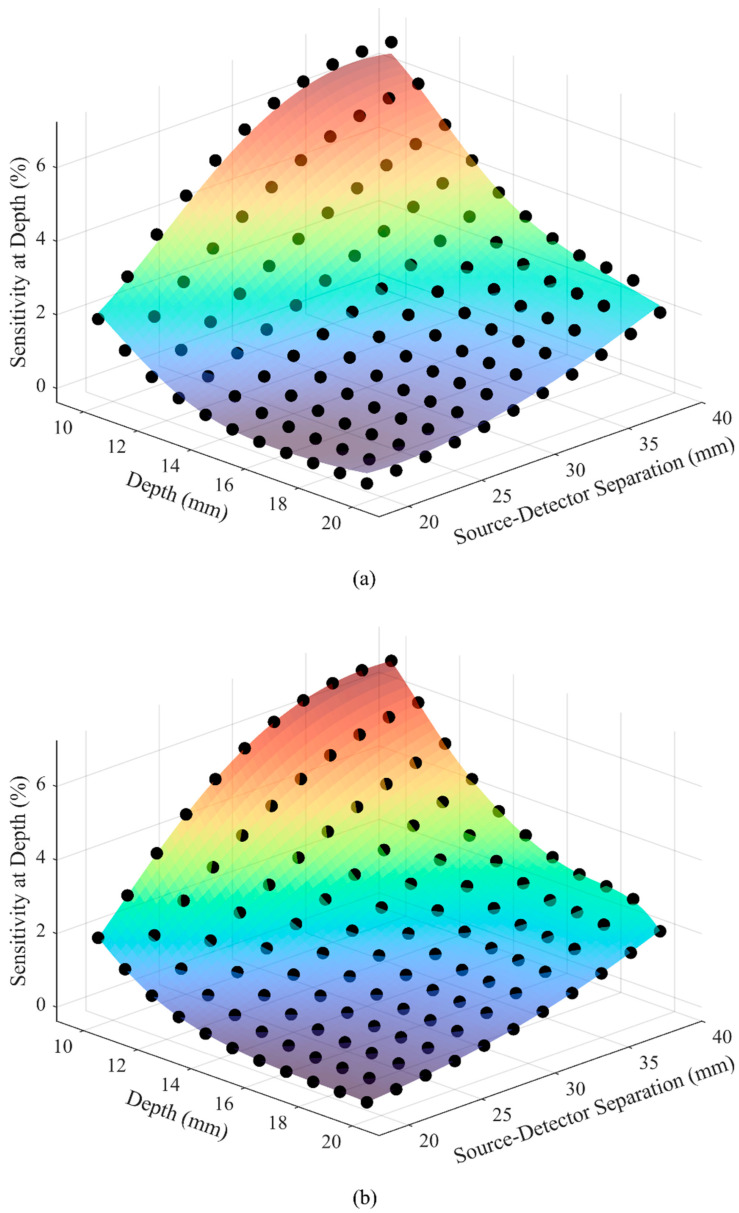
(**a**) SVR-based and (**b**) GPR-based model predictions of the relationship between SDS, depth, and SAD (Dots: Data; Surface: Predicted; Color: SAD magnitude, with dark blue indicating lowest and dark red indicating highest values).

Figure 6 displays the plots for predicted relationship between the SDS, depth, and SAD using SVR and GPR models. In both plots, the colored surfaces represent the model predictions, while the overlaid dots correspond to the original data points. The models were trained using SAD values obtained across a range of SDS and depth combinations. Visual comparison shows how each model captures the trend of SAD variation across the input parameter space. The adjusted R^2^ metric was computed for both models to assess their performance, yielding 0.9902 for SVR and 0.9999 for GPR. While both models showed strong predictive agreement with the data, SVR exhibited slightly reduced accuracy near the boundaries of the input space, whereas GPR provided a closer fit across the full domain and additionally offered uncertainty estimates for its predictions, enhancing model interpretability. For these reasons, GPR was selected for implementation in the DrSVision software.

**Figure 7:**
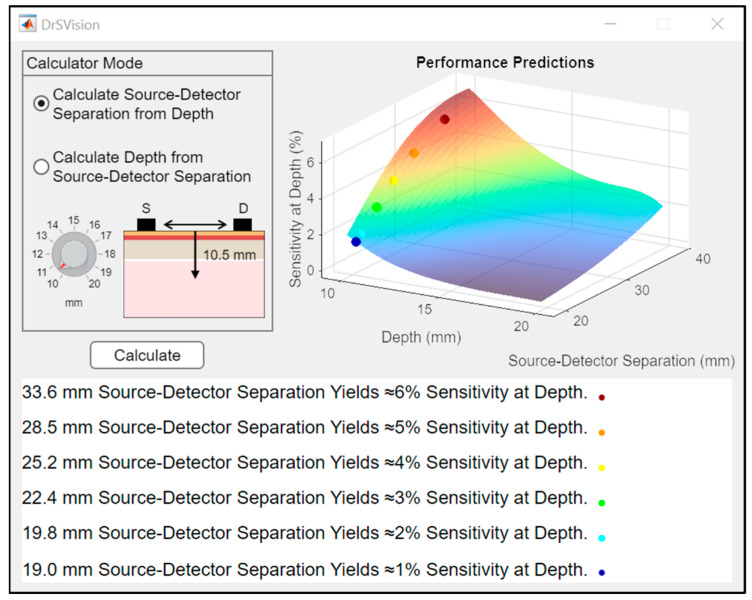
User interface of the designed and developed standalone application DrSVision.

The user interface of the standalone application DrSVision is shown in Figure 7. The application implements the GPR model to provide real time predictions of SAD values based on user input. An interactive 3D surface plot visualizes the relationship between SDS, depth, and SAD, allowing for users to explore model outputs dynamically. This interface represents the functional outcome of the model integration within the developed software.

To illustrate the use of DrSVision, a mock application scenario targeting the dorsolateral PFC is presented in Table 4. The mean scalp-to-cortex distance for the dorsolateral PFC is set to 14.8 mm, based on anatomical references [31]. The user selects the “Calculate Source-Detector Separation from Depth” mode, adjusts the depth input parameter to 14.8 mm using the interface knob (confirmed by the head slice illustration), and presses the “Calculate” button.

DrSVision subsequently provides feasible SDS values corresponding to different SAD levels between 1 and 6%. In this scenario, only SAD values ≤ 3% are feasible. The recommended SDS values for targeting the dorsolateral PFC are summarized in Table 4. This example demonstrates the practical application of DrSVision in generating anatomically informed calibration outputs for fNIRS probe configuration.

## 4. Discussion

This novel study utilized cadaveric heads preserved by formalin fixation followed by fresh-freezing to acquire high-resolution MRI datasets for neuroanatomical analysis. Although this preservation approach altered tissue relaxation properties, longer TR and TE values were applied to optimize image contrast [35,36]. Imaging on a MAGNETOM Prisma Fit system provided a high SNR and spatial resolution, enabling detailed and artifact-free visualization. Consistent scanning parameters ensured reliable comparisons across cadaveric heads and supported 3D reconstructions. No contrast agents were used to preserve cadaveric head integrity [37].

The use of cadaveric heads was essential for spatial accuracy comparisons. Their stability during MRI allowed for prolonged high-resolution scans free from motion artifacts or physiological variability, which would be challenging in living subjects [38]. This stability was critical for voxel-level accuracy and for implementing the extended high-resolution protocols required in this study. Moreover, cadaveric specimens eliminate physiological confounds such as blood pulsation, respiration, and CSF dynamics [39], thereby enabling controlled and repeatable anatomical evaluations across all samples. From a practical standpoint, such protocols would be ethically and logistically difficult to achieve in healthy volunteers. Cadaveric imaging also facilitated anatomically accurate segmentation for MC simulations, enabling repeated measures without in vivo imaging constraints. Notably, variations in tissue thickness across cadaveric heads (see Table 2) further introduced valuable anatomical diversity into the simulation set. Training the GPR-based ML model on mean SAD across structurally distinct heads improved model generalizability and strengthened DrSVision’s applicability across individuals. Boosting and Random Forest models were also tested. These ensemble methods are generally promising: Random Forest reduces variance via multiple trees, and Boosting minimizes bias by sequentially improving weak learners [40]. However, in our dataset, which is characterized by a small sample size, smooth response surfaces, and low-dimensional input space, SVR and GPR outperformed them (R^2^ = 0.9902 for SVR, R^2^ = 0.9999 for GPR, R^2^ = 0.9748 for Boosting, and R^2^ = 0.9168 for Random Forest). This superior performance is attributed to SVR’s ability to model complex non-linear relationships with kernel functions [29] and GPR’s flexibility in capturing smooth functions with probabilistic predictions [30], indicating that, despite the typical strengths of ensemble methods, SVR and GPR were better suited for cadaveric MRI-based predictions. This approach represents a novel integration of cadaveric imaging with GPR-based ML (see Figure 6b) to enhance personalized fNIRS calibration.

The precise anatomical data informed voxel-based MC simulations, realistically modeled NIR light propagation through layered tissue. These simulations formed the backbone of DrSVision, enabling it to predict both the optimal SDS for a target cortical depth and the achievable depth for a given SDS. In the mean SAD vs. depth curves (see Figure 4), SAD peaked at expected depths and then declined, consistent with the canonical “banana-shaped” photon path, validating the model’s physical accuracy [41,42].

Consistent with Okada et al. [33,34], simulations in this study show that superficial tissue thickness reduces sensitivity to deeper cortical regions while increasing SDS enhances depth penetration, but lowers overall signal. Unlike their primarily theoretical models, our approach integrates high-resolution cadaveric MRI- and GPR-based ML, capturing realistic inter-individual anatomical variability. This allowed us not only to reproduce the general trends reported by Okada et al., but also to quantify how optimal SDS must be adjusted per measurement scenario to achieve target cortical sampling, highlighting the importance of region-specific fNIRS probe calibration.

Planar slab geometries ensured consistent photon behavior across heterogeneous layers, and the literature-based optical properties preserved translational relevance [43]. Simulations across eight heads and varying SDS generated high-resolution fluence maps. Post-processing revealed key findings: (1) SAD values varied across cadaveric heads at the same SDS, as shown by vertically overlapping box plots (see Figure 5), highlighting the inherent anatomical variability between individuals. While this study does not resolve these inter-individual differences, documenting and incorporating this variability within the modeling process is an important step toward understanding its impact on fNIRS calibration, emphasizing that individualized fNIRS calibration is the inevitable future for optimizing probe designs and improving overall system performance [23]. Importantly, while participant specific LED power calibration is standard in fNIRS primarily due to inter-individual skin pigmentation differences [44,45], this study emphasizes that SDS should also be adapted per subject, as they depend on anatomical and optical differences across subjects. These parameters are not fixed design constants, but are rather dynamic variables shaped by individual anatomy. This is especially relevant in inter-individual studies, where standard probe configurations may fail to consistently sample the intended brain region [32]; therefore, individualized attention must be given to reduce inter-subject variabilities and enhance fNIRS accuracy.

Beyond individual calibration, this study addresses a broader methodological gap: the absence of tools for study-driven fNIRS probe calibration. fNIRS is often used to study specific PFC subregions, like the dorsolateral PFC for working memory [46], ventromedial PFC for emotional processing [47], or frontopolar cortex for decision-making [48,49], all located at varying cortical depths. The abovementioned regions fall between 10 and 20 mm depths, rationalizing the choice of target range in this study [50]. This ensured that simulations and calibrations focused on anatomically relevant and commonly targeted cortical areas in fNIRS research. Using fixed SDS risks sampling outside the desired target [51,52]. DrSVision addresses this gap by enabling users to specify their region of interest (by depth) and proposing an appropriate source-detector configuration to reach it, advancing precision in functional brain imaging. This represents a novel shift toward neuro-scientifically informed probe calibration, improving spatial measurement sensitivity in fNIRS studies.

Although fNIRS devices vary considerably in their hardware (e.g., light source types, detector sensitivities, and aperture sizes) and software pipelines, the principles of photon transport in tissue remain device-independent for continuous-wave systems. By parameterizing SDS, depth, and SAD variables, DrSVision can be adapted to different platforms. This flexibility highlights the broader applicability of the tool across diverse fNIRS systems.

DrSVision (see Figure 7), developed in MATLAB, offers bidirectional predictions for either depth or separation and outputs SAD estimates across six thresholds (1–6%) [32,53,54] to account for device limitations. Since higher SAD levels require larger SDSs and greater LED output [55], this flexible calibration algorithm allows researchers to work within the optical capacity of their systems. A dynamic 3D surface plot further enhances interpretability, visualizing the relationship between separation, depth and SAD, and supporting evidence-based fNIRS probe design.

While this study presents a robust framework for fNIRS calibration, certain limitations should be noted. Choice of planar slabs were intentional to systematically evaluate hierarchical tissue effects while keeping the model tractable. Not modeling head curvature is a limitation. The planar slab geometry, although computationally efficient, simplifies complex head anatomy and may not fully capture photon path intricacies; however, incorporating curved layers is not straightforward due to substantial individual variability in head geometry, and it is unclear whether this would meaningfully improve simulation accuracy. Planar models remain widely used in fNIRS studies and provide meaningful insights while maintaining computational efficiency [56]. The sample size of eight cadaveric heads, while sufficient to introduce anatomical variability, may limit broader demographic representation. In addition, the age range of the specimens (67–97 years) is skewed toward older adults, which may further restrict the generalizability of the findings. Age-related anatomical changes, such as increased skull thickness, progressive brain atrophy, and alterations in tissue properties, could influence the observed outcomes, and these factors may not accurately reflect characteristics in younger populations. Consequently, caution is warranted when extrapolating the present results beyond the demographics studied. Importantly, since SAD cannot be directly measured experimentally [32,57,58], it is typically computed using MC or similar light-transport simulations, as performed in this and other studies. Consequently, DrSVision was developed based on these theoretical SAD values derived from simulations; empirical validation of the software’s predictive performance remains challenging and was beyond this study’s scope. Another important consideration for broader applicability is addressing data decentralization, an increasingly recognized critical challenge in fNIRS research, largely due to variations in experimental protocols and difficulties in sharing raw data across institutions. Federated learning and multi-center data sharing frameworks have been previously proposed as promising solutions, enabling broader model validation while maintaining privacy and ethical compliance [59,60]. Although DrSVision was developed using a centralized cadaveric dataset, its standardized and device-independent calibration outputs provide a foundation that can facilitate multi-center studies, improve reproducibility, and support data interoperability in future developments.

Future studies should expand the sample size and include more diverse anatomical data to improve model robustness and applicability across populations. Moving beyond planar slab models to curved slab geometries can help to capture individual differences in tissue boundary curvature. Unlike planar layers, where light refraction is uniform, curved layers cause varying incident angles and refraction, affecting photon propagation and better reflecting real head anatomical variabilities. Furthermore, developing more comprehensive experimental validation strategies, such as correlating DrSVision’s predicted SAD values with measured fNIRS signal quality or functional task outcomes in vivo, employing controlled phantom studies or integrating complementary imaging modalities would provide valuable empirical support for DrSVision’s utility. To support these future directions and promote community-driven development, the DrSVision tool will be made freely available as a standalone executable compiled using the MATLAB App Designer. Users will be required to install the free MATLAB Runtime v9.12 from MathWorks (Portola Valley, CA, US) to run the application. The .exe and user instructions will be shared via platforms such as GitHub, Zenodo, and Figshare, and dissemination will be carried out through the Society for functional Near-Infrared Spectroscopy to ensure broad visibility and adoption within the fNIRS research field. These advancements will help bridge the gap between theoretical simulations and practical fNIRS applications, strengthening the calibration framework for broader research and clinical use.

## 5. Conclusions

fNIRS is a promising non-invasive neuroimaging technique, yet its spatial precision is often limited by the use of fixed SDS values, which may not adapt to both individual anatomical variability and neuroscientific study oriented region-specific targeting. This study presents DrSVision, a novel calibration tool that combines MC simulations of cadaveric head MRI data with GPR to estimate SAD for fNIRS. The tool enables region-specific calibration by predicting either the optimal SDS for a desired cortical depth or the achievable depth for a given SDS. MC results demonstrate that SAD is highly sensitive to inter-individual anatomical differences, highlighting the limitations of uniform probe designs across studies.

To the best of our knowledge, such a region-specific ML-integrated calibration framework using anatomically realistic simulation data has never been performed before. By enabling fast, depth-aware, and study-oriented calibration, DrSVision offers a practical step toward more anatomically precise and functionally targeted fNIRS experiments. Future work should focus on incorporating more diverse anatomical datasets, enhancing simulation realism with 3D head models and validating SAD predictions through experimental studies.

## Figures and Tables

**Table 1:** Absorption coefficient, scattering coefficient, anisotropy factor, and refractive index optical properties of each layer in simulation environment. The light wavelengths used for assessing these coefficients were 735 nm [23].

Layers	$\mu_{a}$ (mm^−1^)	$\mu_{s}$ (mm^−1^)	g	n
Void	0	0	0	1
Scalp-Muscle	0.016	19	0.9	1.6
Cranium	0.018	16	0.9	1.56
CSF	0.004	0.3	0	1.33
Brain	0.09	21.5	0.9	1.4

**Table 2:** Thicknesses of scalp-muscle, cranium, CSF, and brain layers for each cadaveric head, measured from MRI data and used in MC simulation models. Mean and standard deviation values across all cadaveric heads are also provided for each layer.

Cadaveric Head	Layers			
	Scalp-Muscle (mm)	Cranium (mm)	CSF (mm)	Brain (mm)
#1	8	6.8	5.55	43.65
#2	6.1	8.1	6.05	43.75
#3	5.2	8	16.1	34.7
#4	4.4	6.9	6.53	46.17
#5	4.4	8.5	0.68	50.42
#6	4	7	7.13	45.87
#7	6	7.8	4.68	45.52
#8	7	9.4	0.6	47
Mean ± Std	5.64 ± 1.4	7.81 ± 0.9	5.92 ± 4.82	44.64 ± 4.54

**Table 3:** Total amount of detected photons for each cadaveric head source—detector combination.

Source-Detector Separation (mm)	Cadaveric Heads							
	#1	#2	#3	#4	#5	#6	#7	#8
19	35,656	34,047	26,219	29,101	48,926	29,295	37,293	44,634
21	24,309	23,589	16,888	20,607	35,238	20,766	26,296	31,196
23	17,497	16,588	11,191	15,062	25,890	15,134	19,134	22,562
25	12,577	11,980	7715	11,769	19,333	11,989	14,196	16,536
27	9289	9168	5501	9301	14,530	9440	10,691	12,438
29	7246	6916	4131	7720	10,986	7821	8326	8865
31	5457	5639	3069	6495	8471	6544	6620	6761
33	4424	4454	2437	5553	6394	5504	5374	5153
35	3434	3643	1940	4728	4878	4632	4312	3737
37	2870	2975	1670	3879	3757	3986	3428	2929
39	2348	2441	1408	3387	2891	3420	2933	2115

**Table 4:** Recommended SDS values for targeting the dorsolateral PFC at a mean scalp-to-cortex depth input of 14.8 mm, as calculated by DrSVision. Corresponding applicable SAD levels are provided.

Depth (mm)	Source-Detector Separation (mm)	Sensitivity at Depth (%)
14.8	-	6
	-	5
	-	4
	37.5	3
	32.5	2
	25.6	1

## Data Availability

The data presented in this study and Supplementary Materials are available on request from the corresponding author.

## Supplementary Materials

The following supporting information can be downloaded at: https://www.mdpi.com/article/10.3390/s25206340/s1, Figure S1: Spatial fluence color maps for cadaveric head #1 at 19 mm source-detector separation from top view (left) and front view (right). The bright white spots indicate the position of the light source. The color bar represents photon fluence in a logarithmic scale to enhance visibility across a wide dynamic range; Figure S2: Spatial fluence color maps for cadaveric head #1 at 21 mm source-detector separation from top view (left) and front view (right). The bright white spots indicate the position of the light source. The color bar represents photon fluence in a logarithmic scale to enhance visibility across a wide dynamic range; Figure S3: Spatial fluence color maps for cadaveric head #1 at 23 mm source-detector separation from top view (left) and front view (right). The bright white spots indicate the position of the light source. The color bar represents photon fluence in a logarithmic scale to enhance visibility across a wide dynamic range; Figure S4: Spatial fluence color maps for cadaveric head #1 at 25 mm source-detector separation from top view (left) and front view (right). The bright white spots indicate the position of the light source. The color bar represents photon fluence in a logarithmic scale to enhance visibility across a wide dynamic range; Figure S5: Spatial fluence color maps for cadaveric head #1 at 27 mm source-detector separation from top view (left) and front view (right). The bright white spots indicate the position of the light source. The color bar represents photon fluence in a logarithmic scale to enhance visibility across a wide dynamic range; Figure S6: Spatial fluence color maps for cadaveric head #1 at 29 mm source-detector separation from top view (left) and front view (right). The bright white spots indicate the position of the light source. The color bar represents photon fluence in a logarithmic scale to enhance visibility across a wide dynamic range; Figure S7: Spatial fluence color maps for cadaveric head #1 at 31 mm source-detector separation from top view (left) and front view (right). The bright white spots indicate the position of the light source. The color bar represents photon fluence in a logarithmic scale to enhance visibility across a wide dynamic range; Figure S8: Spatial fluence color maps for cadaveric head #1 at 33 mm source-detector separation from top view (left) and front view (right). The bright white spots indicate the position of the light source. The color bar represents photon fluence in a logarithmic scale to enhance visibility across a wide dynamic range; Figure S9: Spatial fluence color maps for cadaveric head #1 at 35 mm source-detector separation from top view (left) and front view (right). The bright white spots indicate the position of the light source. The color bar represents photon fluence in a logarithmic scale to enhance visibility across a wide dynamic range; Figure S10: Spatial fluence color maps for cadaveric head #1 at 37 mm source-detector separation from top view (left) and front view (right). The bright white spots indicate the position of the light source. The color bar represents photon fluence in a logarithmic scale to enhance visibility across a wide dynamic range; Figure S11: Spatial fluence color maps for cadaveric head #1 at 39 mm source-detector separation from top view (left) and front view (right). The bright white spots indicate the position of the light source. The color bar represents photon fluence in a logarithmic scale to enhance visibility across a wide dynamic range; Figure S12: Spatial fluence color maps for cadaveric head #2 at 19 mm source-detector separation from top view (left) and front view (right). The bright white spots indicate the position of the light source. The color bar represents photon fluence in a logarithmic scale to enhance visibility across a wide dynamic range; Figure S13: Spatial fluence color maps for cadaveric head #2 at 21 mm source-detector separation from top view (left) and front view (right). The bright white spots indicate the position of the light source. The color bar represents photon fluence in a logarithmic scale to enhance visibility across a wide dynamic range; Figure S14: Spatial fluence color maps for cadaveric head #2 at 23 mm source-detector separation from top view (left) and front view (right). The bright white spots indicate the position of the light source. The color bar represents photon fluence in a logarithmic scale to enhance visibility across a wide dynamic range; Figure S15: Spatial fluence color maps for cadaveric head #2 at 25 mm source-detector separation from top view (left) and front view (right). The bright white spots indicate the position of the light source. The color bar represents photon fluence in a logarithmic scale to enhance visibility across a wide dynamic range; Figure S16: Spatial fluence color maps for cadaveric head #2 at 27 mm source-detector separation from top view (left) and front view (right). The bright white spots indicate the position of the light source. The color bar represents photon fluence in a logarithmic scale to enhance visibility across a wide dynamic range; Figure S17: Spatial fluence color maps for cadaveric head #2 at 29 mm source-detector separation from top view (left) and front view (right). The bright white spots indicate the position of the light source. The color bar represents photon fluence in a logarithmic scale to enhance visibility across a wide dynamic range; Figure S18: Spatial fluence color maps for cadaveric head #2 at 31 mm source-detector separation from top view (left) and front view (right). The bright white spots indicate the position of the light source. The color bar represents photon fluence in a logarithmic scale to enhance visibility across a wide dynamic range; Figure S19: Spatial fluence color maps for cadaveric head #2 at 33 mm source-detector separation from top view (left) and front view (right). The bright white spots indicate the position of the light source. The color bar represents photon fluence in a logarithmic scale to enhance visibility across a wide dynamic range; Figure S20: Spatial fluence color maps for cadaveric head #2 at 35 mm source-detector separation from top view (left) and front view (right). The bright white spots indicate the position of the light source. The color bar represents photon fluence in a logarithmic scale to enhance visibility across a wide dynamic range; Figure S21: Spatial fluence color maps for cadaveric head #2 at 37 mm source-detector separation from top view (left) and front view (right). The bright white spots indicate the position of the light source. The color bar represents photon fluence in a logarithmic scale to enhance visibility across a wide dynamic range; Figure S22: Spatial fluence color maps for cadaveric head #2 at 39 mm source-detector separation from top view (left) and front view (right). The bright white spots indicate the position of the light source. The color bar represents photon fluence in a logarithmic scale to enhance visibility across a wide dynamic range; Figure S23: Spatial fluence color maps for cadaveric head #3 at 19 mm source-detector separation from top view (left) and front view (right). The bright white spots indicate the position of the light source. The color bar represents photon fluence in a logarithmic scale to enhance visibility across a wide dynamic range; Figure S24: Spatial fluence color maps for cadaveric head #3 at 21 mm source-detector separation from top view (left) and front view (right). The bright white spots indicate the position of the light source. The color bar represents photon fluence in a logarithmic scale to enhance visibility across a wide dynamic range; Figure S25: Spatial fluence color maps for cadaveric head #3 at 23 mm source-detector separation from top view (left) and front view (right). The bright white spots indicate the position of the light source. The color bar represents photon fluence in a logarithmic scale to enhance visibility across a wide dynamic range; Figure S26: Spatial fluence color maps for cadaveric head #3 at 25 mm source-detector separation from top view (left) and front view (right). The bright white spots indicate the position of the light source. The color bar represents photon fluence in a logarithmic scale to enhance visibility across a wide dynamic range; Figure S27: Spatial fluence color maps for cadaveric head #3 at 27 mm source-detector separation from top view (left) and front view (right). The bright white spots indicate the position of the light source. The color bar represents photon fluence in a logarithmic scale to enhance visibility across a wide dynamic range; Figure S28: Spatial fluence color maps for cadaveric head #3 at 29 mm source-detector separation from top view (left) and front view (right). The bright white spots indicate the position of the light source. The color bar represents photon fluence in a logarithmic scale to enhance visibility across a wide dynamic range; Figure S29: Spatial fluence color maps for cadaveric head #3 at 31 mm source-detector separation from top view (left) and front view (right). The bright white spots indicate the position of the light source. The color bar represents photon fluence in a logarithmic scale to enhance visibility across a wide dynamic range; Figure S30: Spatial fluence color maps for cadaveric head #3 at 33 mm source-detector separation from top view (left) and front view (right). The bright white spots indicate the position of the light source. The color bar represents photon fluence in a logarithmic scale to enhance visibility across a wide dynamic range; Figure S31: Spatial fluence color maps for cadaveric head #3 at 35 mm source-detector separation from top view (left) and front view (right). The bright white spots indicate the position of the light source. The color bar represents photon fluence in a logarithmic scale to enhance visibility across a wide dynamic range; Figure S32: Spatial fluence color maps for cadaveric head #3 at 37 mm source-detector separation from top view (left) and front view (right). The bright white spots indicate the position of the light source. The color bar represents photon fluence in a logarithmic scale to enhance visibility across a wide dynamic range; Figure S33: Spatial fluence color maps for cadaveric head #3 at 39 mm source-detector separation from top view (left) and front view (right). The bright white spots indicate the position of the light source. The color bar represents photon fluence in a logarithmic scale to enhance visibility across a wide dynamic range; Figure S34: Spatial fluence color maps for cadaveric head #4 at 19 mm source-detector separation from top view (left) and front view (right). The bright white spots indicate the position of the light source. The color bar represents photon fluence in a logarithmic scale to enhance visibility across a wide dynamic range; Figure S35: Spatial fluence color maps for cadaveric head #4 at 21 mm source-detector separation from top view (left) and front view (right). The bright white spots indicate the position of the light source. The color bar represents photon fluence in a logarithmic scale to enhance visibility across a wide dynamic range; Figure S36: Spatial fluence color maps for cadaveric head #4 at 23 mm source-detector separation from top view (left) and front view (right). The bright white spots indicate the position of the light source. The color bar represents photon fluence in a logarithmic scale to enhance visibility across a wide dynamic range; Figure S37: Spatial fluence color maps for cadaveric head #4 at 25 mm source-detector separation from top view (left) and front view (right). The bright white spots indicate the position of the light source. The color bar represents photon fluence in a logarithmic scale to enhance visibility across a wide dynamic range; Figure S38: Spatial fluence color maps for cadaveric head #4 at 27 mm source-detector separation from top view (left) and front view (right). The bright white spots indicate the position of the light source. The color bar represents photon fluence in a logarithmic scale to enhance visibility across a wide dynamic range; Figure S39: Spatial fluence color maps for cadaveric head #4 at 29 mm source-detector separation from top view (left) and front view (right). The bright white spots indicate the position of the light source. The color bar represents photon fluence in a logarithmic scale to enhance visibility across a wide dynamic range; Figure S40: Spatial fluence color maps for cadaveric head #4 at 31 mm source-detector separation from top view (left) and front view (right). The bright white spots indicate the position of the light source. The color bar represents photon fluence in a logarithmic scale to enhance visibility across a wide dynamic range; Figure S41: Spatial fluence color maps for cadaveric head #4 at 33 mm source-detector separation from top view (left) and front view (right). The bright white spots indicate the position of the light source. The color bar represents photon fluence in a logarithmic scale to enhance visibility across a wide dynamic range; Figure S42: Spatial fluence color maps for cadaveric head #4 at 35 mm source-detector separation from top view (left) and front view (right). The bright white spots indicate the position of the light source. The color bar represents photon fluence in a logarithmic scale to enhance visibility across a wide dynamic range; Figure S43: Spatial fluence color maps for cadaveric head #4 at 37 mm source-detector separation from top view (left) and front view (right). The bright white spots indicate the position of the light source. The color bar represents photon fluence in a logarithmic scale to enhance visibility across a wide dynamic range; Figure S44: Spatial fluence color maps for cadaveric head #4 at 39 mm source-detector separation from top view (left) and front view (right). The bright white spots indicate the position of the light source. The color bar represents photon fluence in a logarithmic scale to enhance visibility across a wide dynamic range; Figure S45: Spatial fluence color maps for cadaveric head #5 at 19 mm source-detector separation from top view (left) and front view (right). The bright white spots indicate the position of the light source. The color bar represents photon fluence in a logarithmic scale to enhance visibility across a wide dynamic range; Figure S46: Spatial fluence color maps for cadaveric head #5 at 21 mm source-detector separation from top view (left) and front view (right). The bright white spots indicate the position of the light source. The color bar represents photon fluence in a logarithmic scale to enhance visibility across a wide dynamic range; Figure S47: Spatial fluence color maps for cadaveric head #5 at 23 mm source-detector separation from top view (left) and front view (right). The bright white spots indicate the position of the light source. The color bar represents photon fluence in a logarithmic scale to enhance visibility across a wide dynamic range; Figure S48: Spatial fluence color maps for cadaveric head #5 at 25 mm source-detector separation from top view (left) and front view (right). The bright white spots indicate the position of the light source. The color bar represents photon fluence in a logarithmic scale to enhance visibility across a wide dynamic range; Figure S49: Spatial fluence color maps for cadaveric head #5 at 27 mm source-detector separation from top view (left) and front view (right). The bright white spots indicate the position of the light source. The color bar represents photon fluence in a logarithmic scale to enhance visibility across a wide dynamic range; Figure S50: Spatial fluence color maps for cadaveric head #5 at 29 mm source-detector separation from top view (left) and front view (right). The bright white spots indicate the position of the light source. The color bar represents photon fluence in a logarithmic scale to enhance visibility across a wide dynamic range; Figure S51: Spatial fluence color maps for cadaveric head #5 at 31 mm source-detector separation from top view (left) and front view (right). The bright white spots indicate the position of the light source. The color bar represents photon fluence in a logarithmic scale to enhance visibility across a wide dynamic range; Figure S52: Spatial fluence color maps for cadaveric head #5 at 33 mm source-detector separation from top view (left) and front view (right). The bright white spots indicate the position of the light source. The color bar represents photon fluence in a logarithmic scale to enhance visibility across a wide dynamic range; Figure S53: Spatial fluence color maps for cadaveric head #5 at 35 mm source-detector separation from top view (left) and front view (right). The bright white spots indicate the position of the light source. The color bar represents photon fluence in a logarithmic scale to enhance visibility across a wide dynamic range; Figure S54: Spatial fluence color maps for cadaveric head #5 at 37 mm source-detector separation from top view (left) and front view (right). The bright white spots indicate the position of the light source. The color bar represents photon fluence in a logarithmic scale to enhance visibility across a wide dynamic range; Figure S55: Spatial fluence color maps for cadaveric head #5 at 39 mm source-detector separation from top view (left) and front view (right). The bright white spots indicate the position of the light source. The color bar represents photon fluence in a logarithmic scale to enhance visibility across a wide dynamic range; Figure S56: Spatial fluence color maps for cadaveric head #6 at 19 mm source-detector separation from top view (left) and front view (right). The bright white spots indicate the position of the light source. The color bar represents photon fluence in a logarithmic scale to enhance visibility across a wide dynamic range; Figure S57: Spatial fluence color maps for cadaveric head #6 at 21 mm source-detector separation from top view (left) and front view (right). The bright white spots indicate the position of the light source. The color bar represents photon fluence in a logarithmic scale to enhance visibility across a wide dynamic range; Figure S58: Spatial fluence color maps for cadaveric head #6 at 23 mm source-detector separation from top view (left) and front view (right). The bright white spots indicate the position of the light source. The color bar represents photon fluence in a logarithmic scale to enhance visibility across a wide dynamic range; Figure S59: Spatial fluence color maps for cadaveric head #6 at 25 mm source-detector separation from top view (left) and front view (right). The bright white spots indicate the position of the light source. The color bar represents photon fluence in a logarithmic scale to enhance visibility across a wide dynamic range; Figure S60: Spatial fluence color maps for cadaveric head #6 at 27 mm source-detector separation from top view (left) and front view (right). The bright white spots indicate the position of the light source. The color bar represents photon fluence in a logarithmic scale to enhance visibility across a wide dynamic range; Figure S61: Spatial fluence color maps for cadaveric head #6 at 29 mm source-detector separation from top view (left) and front view (right). The bright white spots indicate the position of the light source. The color bar represents photon fluence in a logarithmic scale to enhance visibility across a wide dynamic range; Figure S62: Spatial fluence color maps for cadaveric head #6 at 31 mm source-detector separation from top view (left) and front view (right). The bright white spots indicate the position of the light source. The color bar represents photon fluence in a logarithmic scale to enhance visibility across a wide dynamic range; Figure S63: Spatial fluence color maps for cadaveric head #6 at 33 mm source-detector separation from top view (left) and front view (right). The bright white spots indicate the position of the light source. The color bar represents photon fluence in a logarithmic scale to enhance visibility across a wide dynamic range; Figure S64: Spatial fluence color maps for cadaveric head #6 at 35 mm source-detector separation from top view (left) and front view (right). The bright white spots indicate the position of the light source. The color bar represents photon fluence in a logarithmic scale to enhance visibility across a wide dynamic range; Figure S65: Spatial fluence color maps for cadaveric head #6 at 37 mm source-detector separation from top view (left) and front view (right). The bright white spots indicate the position of the light source. The color bar represents photon fluence in a logarithmic scale to enhance visibility across a wide dynamic range; Figure S66: Spatial fluence color maps for cadaveric head #6 at 39 mm source-detector separation from top view (left) and front view (right). The bright white spots indicate the position of the light source. The color bar represents photon fluence in a logarithmic scale to enhance visibility across a wide dynamic range; Figure S67: Spatial fluence color maps for cadaveric head #7 at 19 mm source-detector separation from top view (left) and front view (right). The bright white spots indicate the position of the light source. The color bar represents photon fluence in a logarithmic scale to enhance visibility across a wide dynamic range; Figure S68: Spatial fluence color maps for cadaveric head #7 at 21 mm source-detector separation from top view (left) and front view (right). The bright white spots indicate the position of the light source. The color bar represents photon fluence in a logarithmic scale to enhance visibility across a wide dynamic range; Figure S69: Spatial fluence color maps for cadaveric head #7 at 23 mm source-detector separation from top view (left) and front view (right). The bright white spots indicate the position of the light source. The color bar represents photon fluence in a logarithmic scale to enhance visibility across a wide dynamic range; Figure S70: Spatial fluence color maps for cadaveric head #7 at 25 mm source-detector separation from top view (left) and front view (right). The bright white spots indicate the position of the light source. The color bar represents photon fluence in a logarithmic scale to enhance visibility across a wide dynamic range; Figure S71: Spatial fluence color maps for cadaveric head #7 at 27 mm source-detector separation from top view (left) and front view (right). The bright white spots indicate the position of the light source. The color bar represents photon fluence in a logarithmic scale to enhance visibility across a wide dynamic range; Figure S72: Spatial fluence color maps for cadaveric head #7 at 29 mm source-detector separation from top view (left) and front view (right). The bright white spots indicate the position of the light source. The color bar represents photon fluence in a logarithmic scale to enhance visibility across a wide dynamic range; Figure S73: Spatial fluence color maps for cadaveric head #7 at 31 mm source-detector separation from top view (left) and front view (right). The bright white spots indicate the position of the light source. The color bar represents photon fluence in a logarithmic scale to enhance visibility across a wide dynamic range; Figure S74: Spatial fluence color maps for cadaveric head #7 at 33 mm source-detector separation from top view (left) and front view (right). The bright white spots indicate the position of the light source. The color bar represents photon fluence in a logarithmic scale to enhance visibility across a wide dynamic range; Figure S75: Spatial fluence color maps for cadaveric head #7 at 35 mm source-detector separation from top view (left) and front view (right). The bright white spots indicate the position of the light source. The color bar represents photon fluence in a logarithmic scale to enhance visibility across a wide dynamic range; Figure S76: Spatial fluence color maps for cadaveric head #7 at 37 mm source-detector separation from top view (left) and front view (right). The bright white spots indicate the position of the light source. The color bar represents photon fluence in a logarithmic scale to enhance visibility across a wide dynamic range; Figure S77: Spatial fluence color maps for cadaveric head #7 at 39 mm source-detector separation from top view (left) and front view (right). The bright white spots indicate the position of the light source. The color bar represents photon fluence in a logarithmic scale to enhance visibility across a wide dynamic range; Figure S78: Spatial fluence color maps for cadaveric head #8 at 19 mm source-detector separation from top view (left) and front view (right). The bright white spots indicate the position of the light source. The color bar represents photon fluence in a logarithmic scale to enhance visibility across a wide dynamic range; Figure S79: Spatial fluence color maps for cadaveric head #8 at 21 mm source-detector separation from top view (left) and front view (right). The bright white spots indicate the position of the light source. The color bar represents photon fluence in a logarithmic scale to enhance visibility across a wide dynamic range; Figure S80: Spatial fluence color maps for cadaveric head #8 at 23 mm source-detector separation from top view (left) and front view (right). The bright white spots indicate the position of the light source. The color bar represents photon fluence in a logarithmic scale to enhance visibility across a wide dynamic range; Figure S81: Spatial fluence color maps for cadaveric head #8 at 25 mm source-detector separation from top view (left) and front view (right). The bright white spots indicate the position of the light source. The color bar represents photon fluence in a logarithmic scale to enhance visibility across a wide dynamic range; Figure S82: Spatial fluence color maps for cadaveric head #8 at 27 mm source-detector separation from top view (left) and front view (right). The bright white spots indicate the position of the light source. The color bar represents photon fluence in a logarithmic scale to enhance visibility across a wide dynamic range; Figure S83: Spatial fluence color maps for cadaveric head #8 at 29 mm source-detector separation from top view (left) and front view (right). The bright white spots indicate the position of the light source. The color bar represents photon fluence in a logarithmic scale to enhance visibility across a wide dynamic range; Figure S84: Spatial fluence color maps for cadaveric head #8 at 31 mm source-detector separation from top view (left) and front view (right). The bright white spots indicate the position of the light source. The color bar represents photon fluence in a logarithmic scale to enhance visibility across a wide dynamic range; Figure S85: Spatial fluence color maps for cadaveric head #8 at 33 mm source-detector separation from top view (left) and front view (right). The bright white spots indicate the position of the light source. The color bar represents photon fluence in a logarithmic scale to enhance visibility across a wide dynamic range; Figure S86: Spatial fluence color maps for cadaveric head #8 at 35 mm source-detector separation from top view (left) and front view (right). The bright white spots indicate the position of the light source. The color bar represents photon fluence in a logarithmic scale to enhance visibility across a wide dynamic range; Figure S87: Spatial fluence color maps for cadaveric head #8 at 37 mm source-detector separation from top view (left) and front view (right). The bright white spots indicate the position of the light source. The color bar represents photon fluence in a logarithmic scale to enhance visibility across a wide dynamic range; Figure S88: Spatial fluence color maps for cadaveric head #8 at 39 mm source-detector separation from top view (left) and front view (right). The bright white spots indicate the position of the light source. The color bar represents photon fluence in a logarithmic scale to enhance visibility across a wide dynamic range; Figure S89: Sensitivity at depth vs. depth 2D plots for all cadaveric heads at 19 mm source-detector separation. Curves correspond to Heads #1–8, shown from lightest to darkest red. For clarity, curves for Heads #2–8 are vertically shifted in 2% increments to reduce overlap; Figure S90: Sensitivity at depth vs. depth 2D plots for all cadaveric heads at 21 mm source-detector separation. Curves correspond to Heads #1–8, shown from lightest to darkest red. For clarity, curves for Heads #2–8 are vertically shifted in 2% increments to reduce overlap; Figure S91: Sensitivity at depth vs. depth 2D plots for all cadaveric heads at 23 mm source-detector separation. Curves correspond to Heads #1–8, shown from lightest to darkest red. For clarity, curves for Heads #2–8 are vertically shifted in 2% increments to reduce overlap; Figure S92: Sensitivity at depth vs. depth 2D plots for all cadaveric heads at 25 mm source-detector separation. Curves correspond to Heads #1–8, shown from lightest to darkest red. For clarity, curves for Heads #2–8 are vertically shifted in 2% increments to reduce overlap; Figure S93: Sensitivity at depth vs. depth 2D plots for all cadaveric heads at 27 mm source-detector separation. Curves correspond to Heads #1–8, shown from lightest to darkest red. For clarity, curves for Heads #2–8 are vertically shifted in 2% increments to reduce overlap; Figure S94: Sensitivity at depth vs. depth 2D plots for all cadaveric heads at 29 mm source-detector separation. Curves correspond to Heads #1–8, shown from lightest to darkest red. For clarity, curves for Heads #2–8 are vertically shifted in 2% increments to reduce overlap; Figure S95: Sensitivity at depth vs. depth 2D plots for all cadaveric heads at 31 mm source-detector separation. Curves correspond to Heads #1–8, shown from lightest to darkest red. For clarity, curves for Heads #2–8 are vertically shifted in 2% increments to reduce overlap; Figure S96: Sensitivity at depth vs. depth 2D plots for all cadaveric heads at 33 mm source-detector separation. Curves correspond to Heads #1–8, shown from lightest to darkest red. For clarity, curves for Heads #2–8 are vertically shifted in 2% increments to reduce overlap; Figure S97: Sensitivity at depth vs. depth 2D plots for all cadaveric heads at 35 mm source-detector separation. Curves correspond to Heads #1–8, shown from lightest to darkest red. For clarity, curves for Heads #2–8 are vertically shifted in 2% increments to reduce overlap; Figure S98: Sensitivity at depth vs. depth 2D plots for all cadaveric heads at 37 mm source-detector separation. Curves correspond to Heads #1–8, shown from lightest to darkest red. For clarity, curves for Heads #2–8 are vertically shifted in 2% increments to reduce overlap; Figure S99: Sensitivity at depth vs. depth 2D plots for all cadaveric heads at 39 mm source-detector separation. Curves correspond to Heads #1–8, shown from lightest to darkest red. For clarity, curves for Heads #2–8 are vertically shifted in 2% increments to reduce overlap; Figure S100: Sensitivity at depth vs. source-detector separation box plots at 10 mm depth. (Each box plot is generated over all cadaveric heads); Figure S101: Sensitivity at depth vs. source-detector separation box plots at 11 mm depth. (Each box plot is generated over all cadaveric heads); Figure S102: Sensitivity at depth vs. source-detector separation box plots at 12 mm depth. (Each box plot is generated over all cadaveric heads); Figure S103: Sensitivity at depth vs. source-detector separation box plots at 13 mm depth. (Each box plot is generated over all cadaveric heads); Figure S104: Sensitivity at depth vs. source-detector separation box plots at 14 mm depth. (Each box plot is generated over all cadaveric heads); Figure S105: Sensitivity at depth vs. source-detector separation box plots at 15 mm depth. (Each box plot is generated over all cadaveric heads); Figure S106: Sensitivity at depth vs. source-detector separation box plots at 16 mm depth. (Each box plot is generated over all cadaveric heads; Figure S107: Sensitivity at depth vs. source-detector separation box plots at 17 mm depth. (Each box plot is generated over all cadaveric heads); Figure S108: Sensitivity at depth vs. source-detector separation box plots at 18 mm depth. (Each box plot is generated over all cadaveric heads); Figure S109: Sensitivity at depth vs. source-detector separation box plots at 19 mm depth. (Each box plot is generated over all cadaveric heads); Figure S110: Sensitivity at depth vs. source-detector separation box plots at 20 mm depth. (Each box plot is generated over all cadaveric heads); Figure S111: Photon count vs. path length histogram at 19 mm source-detector separation. Each histogram bin is generated over mean across all cadaveric heads for that data range. Vertical blue dashed line shows mean across all data; Figure S112: Photon count vs. path length histogram at 21 mm source-detector separation. Each histogram bin is generated over mean across all cadaveric heads for that data range. Vertical blue dashed line shows mean across all data; Figure S113: Photon count vs. path length histogram at 23 mm source-detector separation. Each histogram bin is generated over mean across all cadaveric heads for that data range. Vertical blue dashed line shows mean across all data; Figure S114: Photon count vs. path length histogram at 25 mm source-detector separation. Each histogram bin is generated over mean across all cadaveric heads for that data range. Vertical blue dashed line shows mean across all data; Figure S115: Photon count vs. path length histogram at 27 mm source-detector separation. Each histogram bin is generated over mean across all cadaveric heads for that data range. Vertical blue dashed line shows mean across all data; Figure S116: Photon count vs. path length histogram at 29 mm source-detector separation. Each histogram bin is generated over mean across all cadaveric heads for that data range. Vertical blue dashed line shows mean across all data; Figure S117: Photon count vs. path length histogram at 31 mm source-detector separation. Each histogram bin is generated over mean across all cadaveric heads for that data range. Vertical blue dashed line shows mean across all data; Figure S118: Photon count vs. path length histogram at 33 mm source-detector separation. Each histogram bin is generated over mean across all cadaveric heads for that data range. Vertical blue dashed line shows mean across all data; Figure S119: Photon count vs. path length histogram at 35 mm source-detector separation. Each histogram bin is generated over mean across all cadaveric heads for that data range. Vertical blue dashed line shows mean across all data; Figure S120: Photon count vs. path length histogram at 37 mm source-detector separation. Each histogram bin is generated over mean across all cadaveric heads for that data range. Vertical blue dashed line shows mean across all data; Figure S121: Photon count vs. path length histogram at 39 mm source-detector separation. Each histogram bin is generated over mean across all cadaveric heads for that data range. Vertical blue dashed line shows mean across all data.
