# Supplementary material for: DrSVision: A Machine Learning Tool for Cortical Region-Specific fNIRS Calibration Based on Cadaveric Head MRI
Source: Sensors (Basel). 2025 Oct 14;25(20):6340. doi: 10.3390/s25206340 (PMC12567750; doi:10.3390/s25206340)
Supplement: Supplementary file 1 [file sensors-25-06340-s001.zip › sensors-3810043-supplementary.pdf]

## Supplementary Materials

### A. Spatial Fluence (Color Maps)

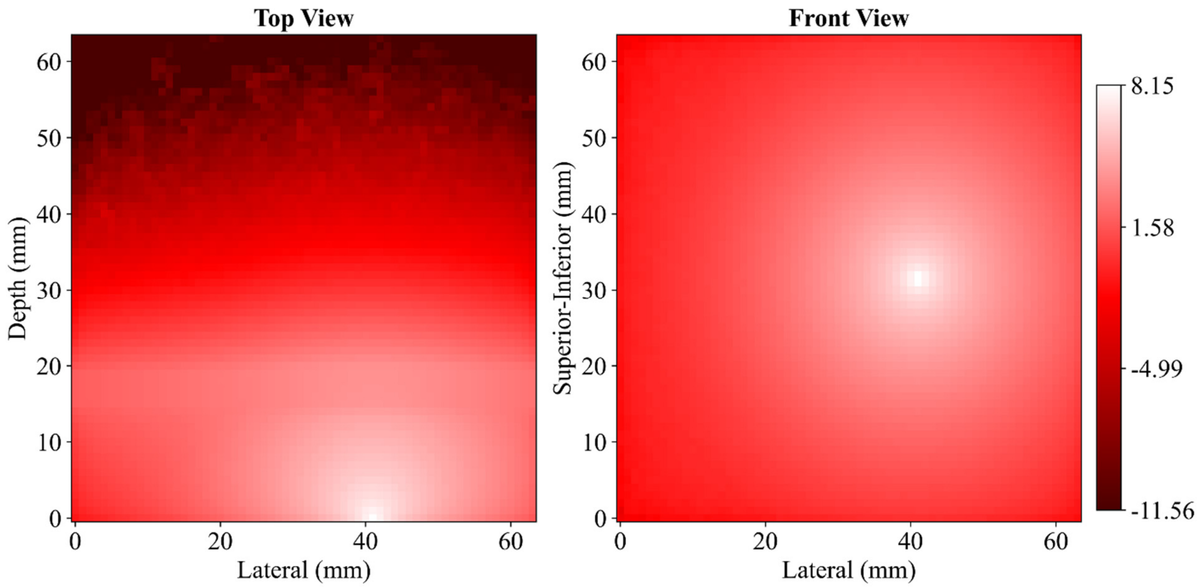


**Figure S1** Spatial fluence color maps for cadaveric head #1 at 19 mm source-detector separation from top view (left) and front view (right). The bright white spots indicate the position of the light source. The color bar represents photon fluence in a logarithmic scale to enhance visibility across a wide dynamic range.

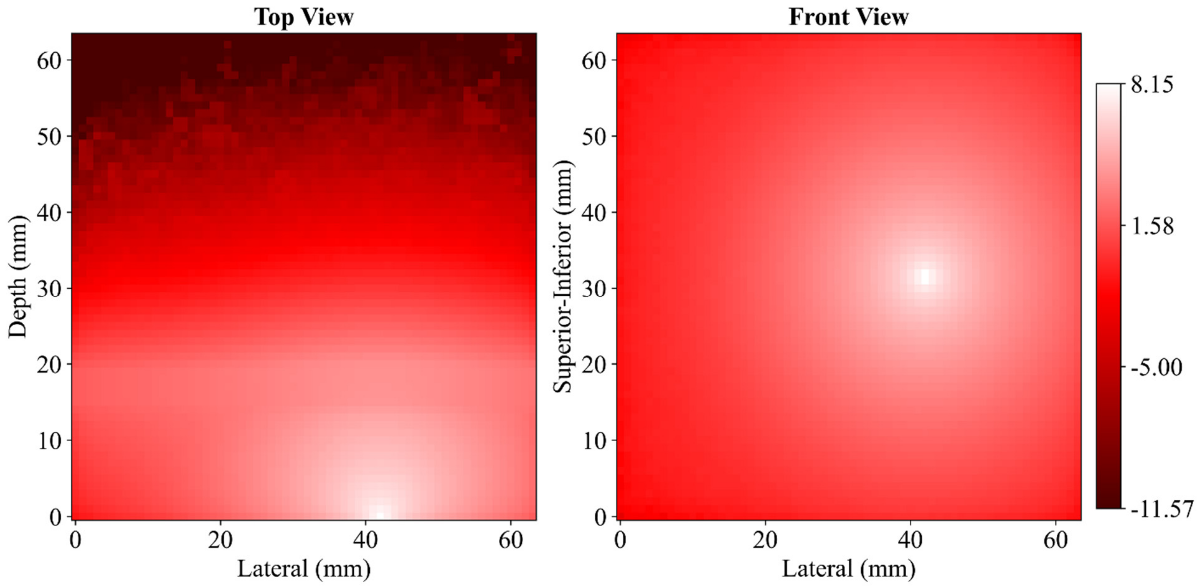


**Figure S2** Spatial fluence color maps for cadaveric head #1 at 21 mm source-detector separation from top view (left) and front view (right). The bright white spots indicate the position of the light source. The color bar represents photon fluence in a logarithmic scale to enhance visibility across a wide dynamic range.

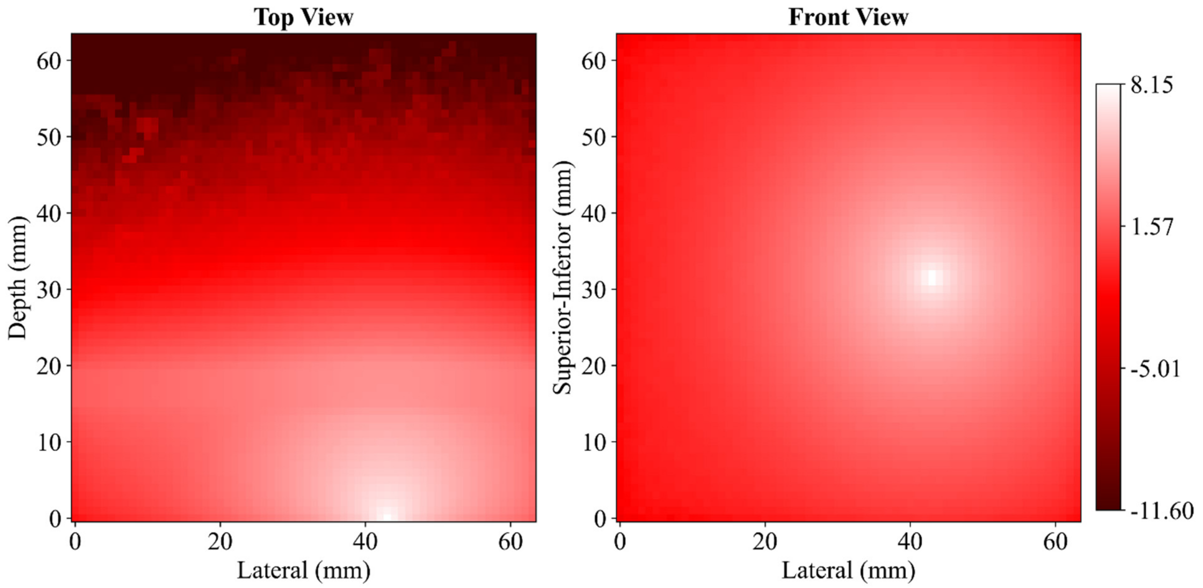


**Figure S3** Spatial fluence color maps for cadaveric head #1 at 23 mm source-detector separation from top view (left) and front view (right). The bright white spots indicate the position of the light source. The color bar represents photon fluence in a logarithmic scale to enhance visibility across a wide dynamic range.

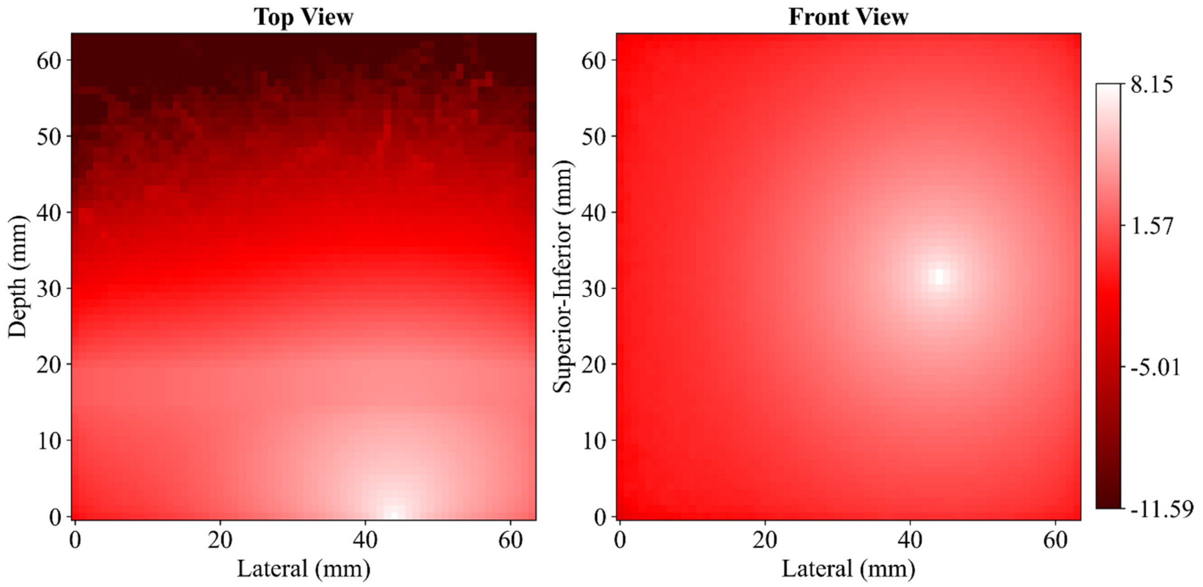


**Figure S4** Spatial fluence color maps for cadaveric head #1 at 25 mm source-detector separation from top view (left) and front view (right). The bright white spots indicate the position of the light source. The color bar represents photon fluence in a logarithmic scale to enhance visibility across a wide dynamic range.

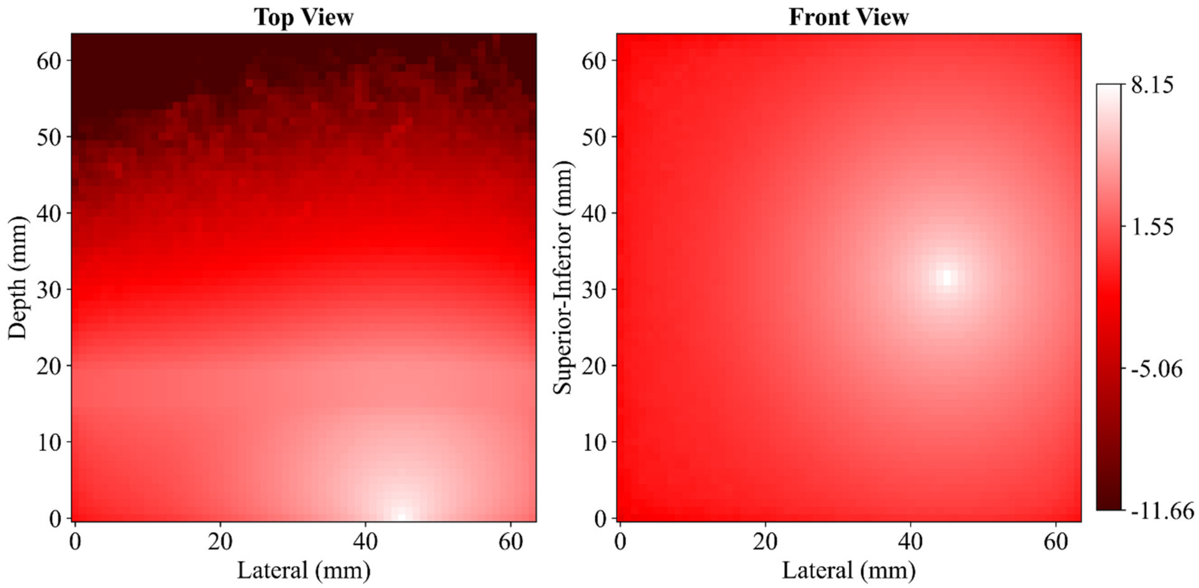


**Figure S5** Spatial fluence color maps for cadaveric head #1 at 27 mm source-detector separation from top view (left) and front view (right). The bright white spots indicate the position of the light source. The color bar represents photon fluence in a logarithmic scale to enhance visibility across a wide dynamic range.

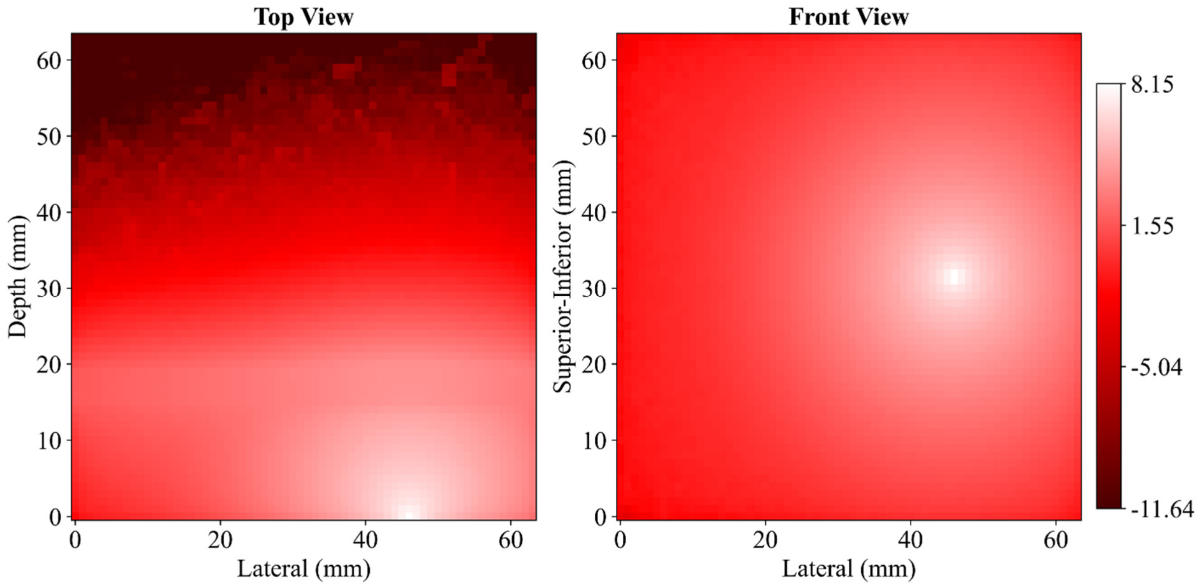


**Figure S6** Spatial fluence color maps for cadaveric head #1 at 29 mm source-detector separation from top view (left) and front view (right). The bright white spots indicate the position of the light source. The color bar represents photon fluence in a logarithmic scale to enhance visibility across a wide dynamic range.

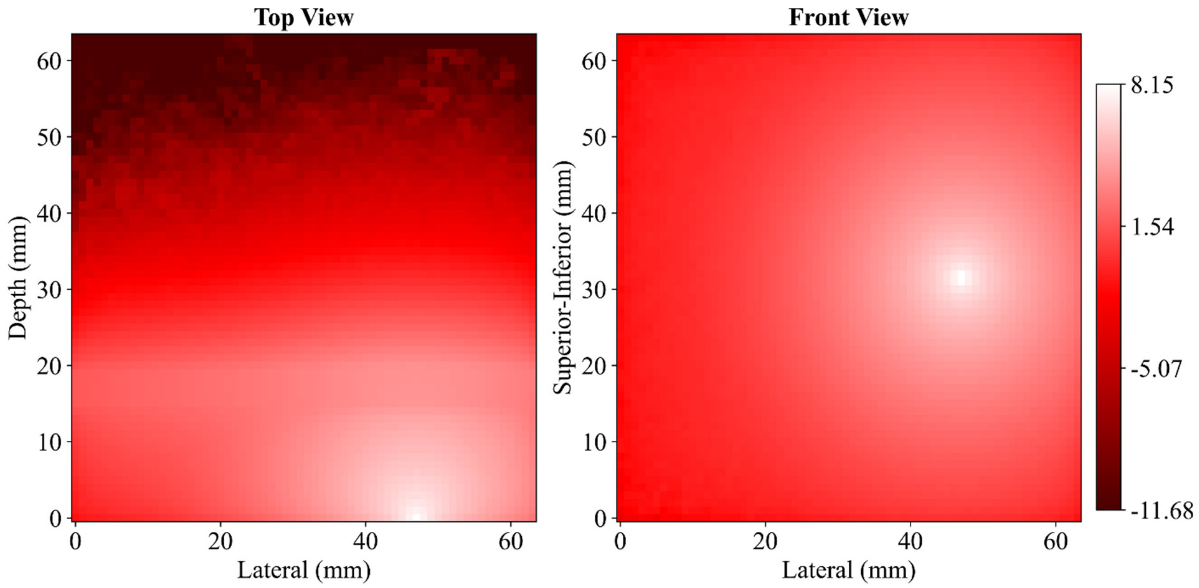


**Figure S7** Spatial fluence color maps for cadaveric head #1 at 31 mm source-detector separation from top view (left) and front view (right). The bright white spots indicate the position of the light source. The color bar represents photon fluence in a logarithmic scale to enhance visibility across a wide dynamic range.

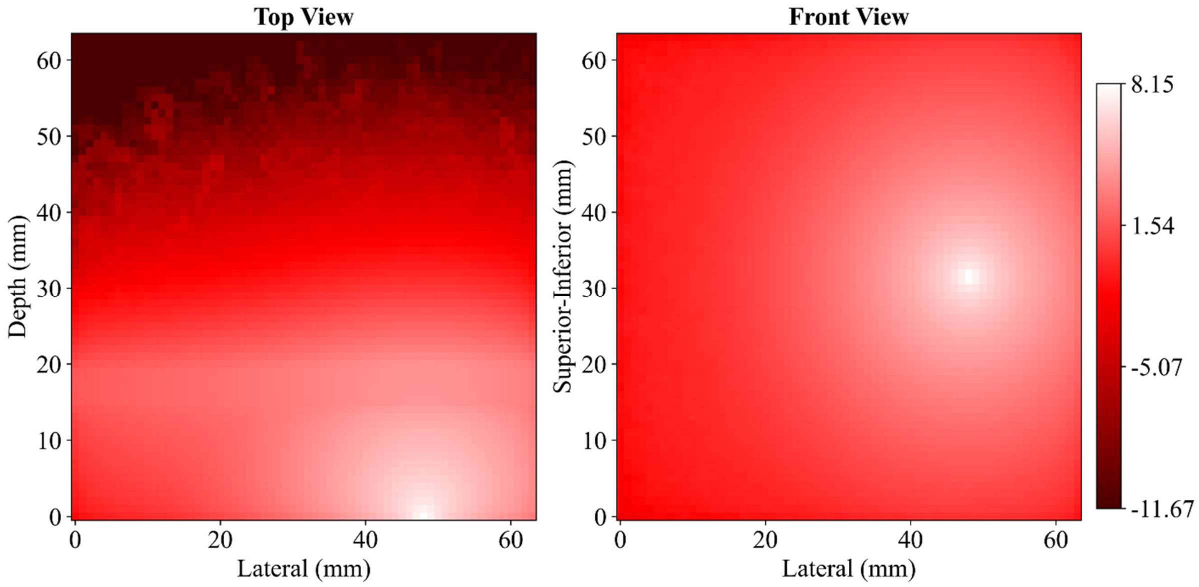


**Figure S8** Spatial fluence color maps for cadaveric head #1 at 33 mm source-detector separation from top view (left) and front view (right). The bright white spots indicate the position of the light source. The color bar represents photon fluence in a logarithmic scale to enhance visibility across a wide dynamic range.

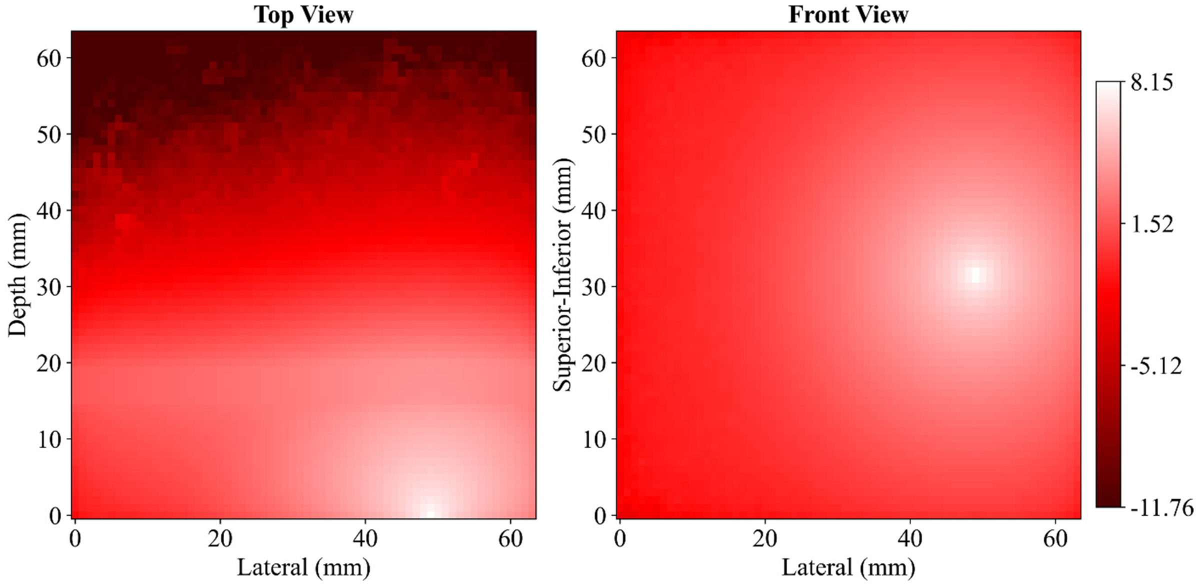


**Figure S9** Spatial fluence color maps for cadaveric head #1 at 35 mm source-detector separation from top view (left) and front view (right). The bright white spots indicate the position of the light source. The color bar represents photon fluence in a logarithmic scale to enhance visibility across a wide dynamic range.

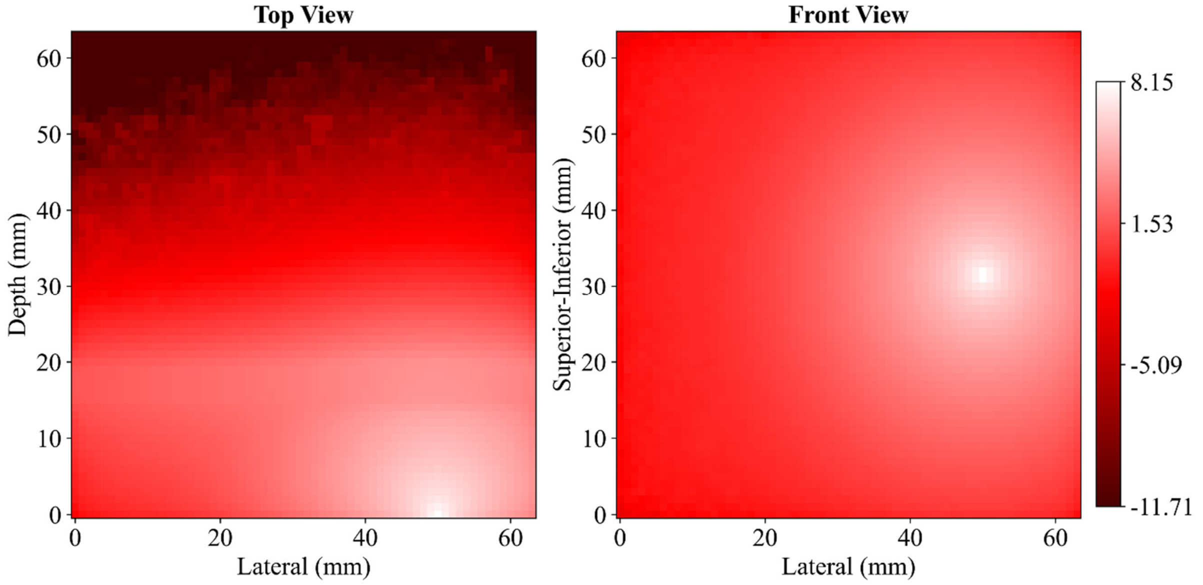


**Figure S10** Spatial fluence color maps for cadaveric head #1 at 37 mm source-detector separation from top view (left) and front view (right). The bright white spots indicate the position of the light source. The color bar represents photon fluence in a logarithmic scale to enhance visibility across a wide dynamic range.

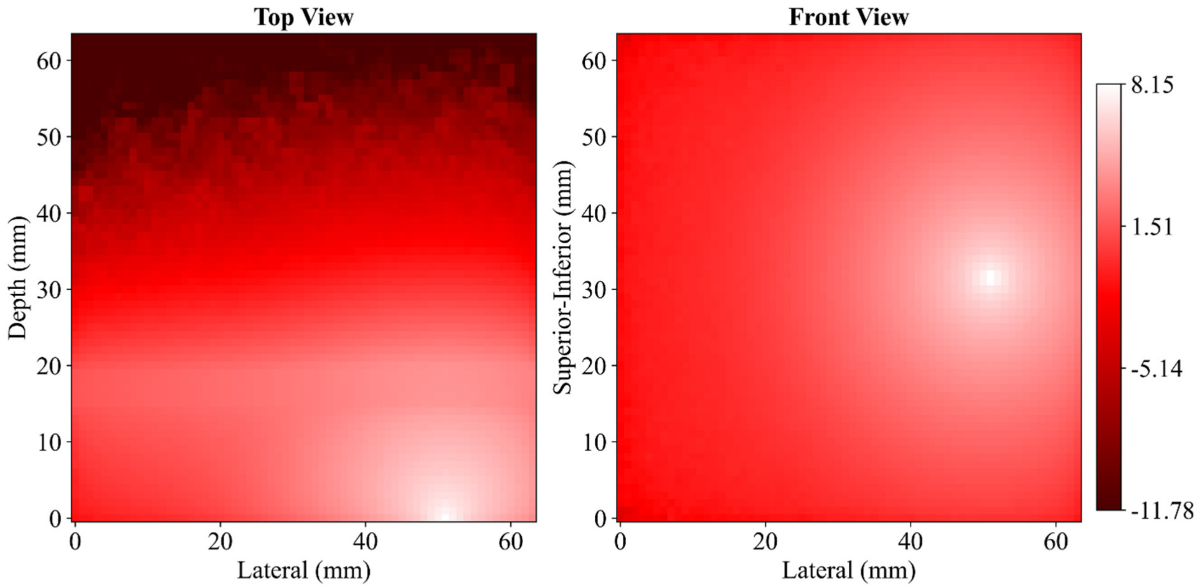


**Figure S11** Spatial fluence color maps for cadaveric head #1 at 39 mm source-detector separation from top view (left) and front view (right). The bright white spots indicate the position of the light source. The color bar represents photon fluence in a logarithmic scale to enhance visibility across a wide dynamic range.

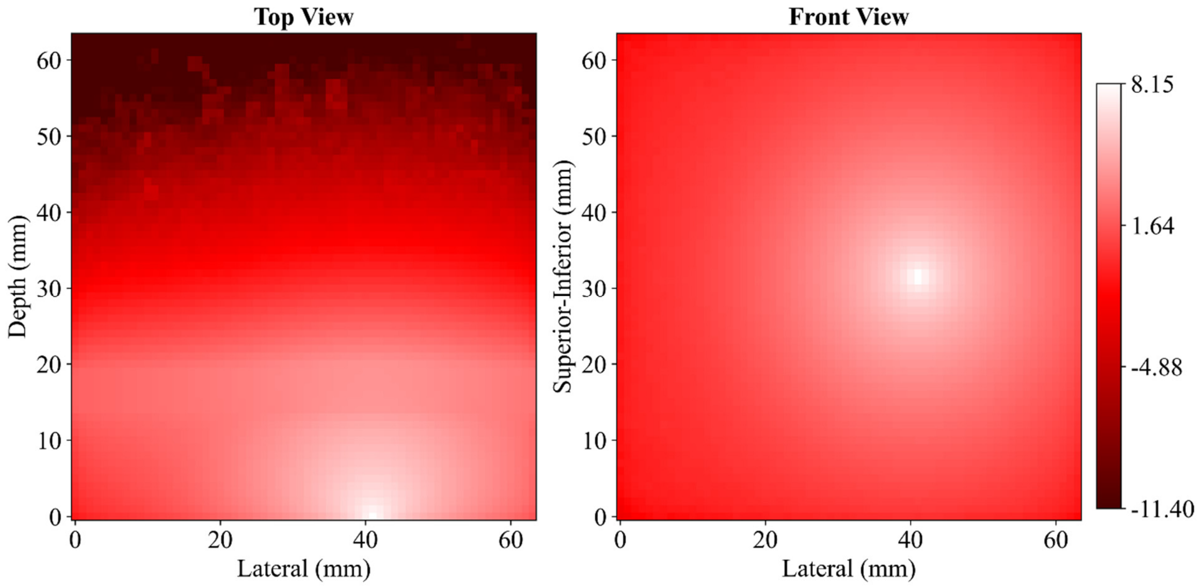


**Figure S12** Spatial fluence color maps for cadaveric head #2 at 19 mm source-detector separation from top view (left) and front view (right). The bright white spots indicate the position of the light source. The color bar represents photon fluence in a logarithmic scale to enhance visibility across a wide dynamic range.

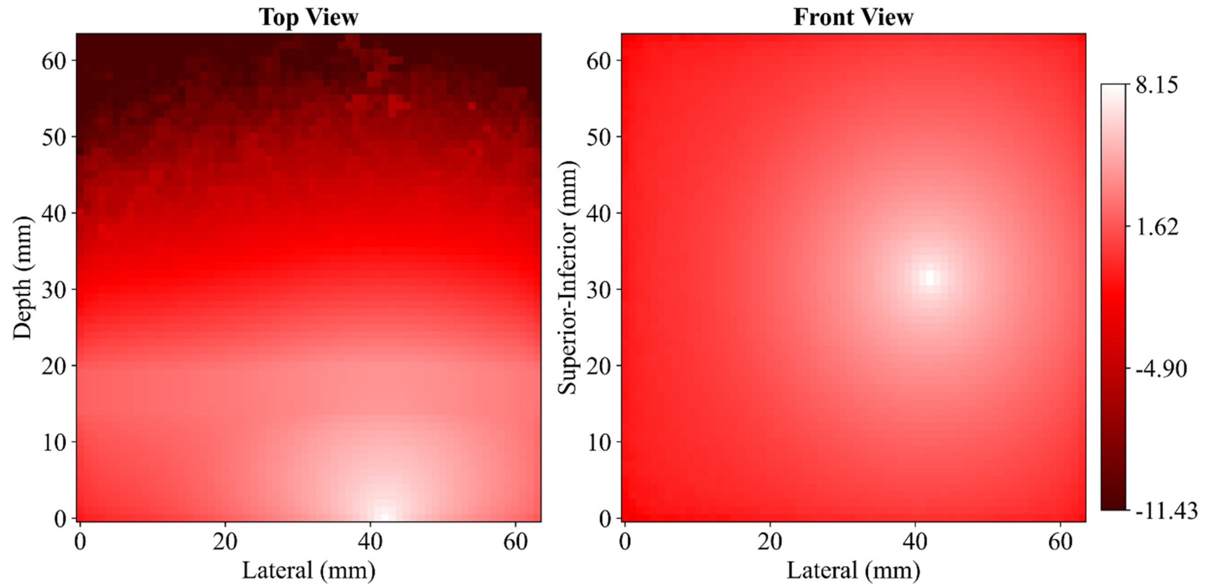


**Figure S13** Spatial fluence color maps for cadaveric head #2 at 21 mm source-detector separation from top view (left) and front view (right). The bright white spots indicate the position of the light source. The color bar represents photon fluence in a logarithmic scale to enhance visibility across a wide dynamic range.

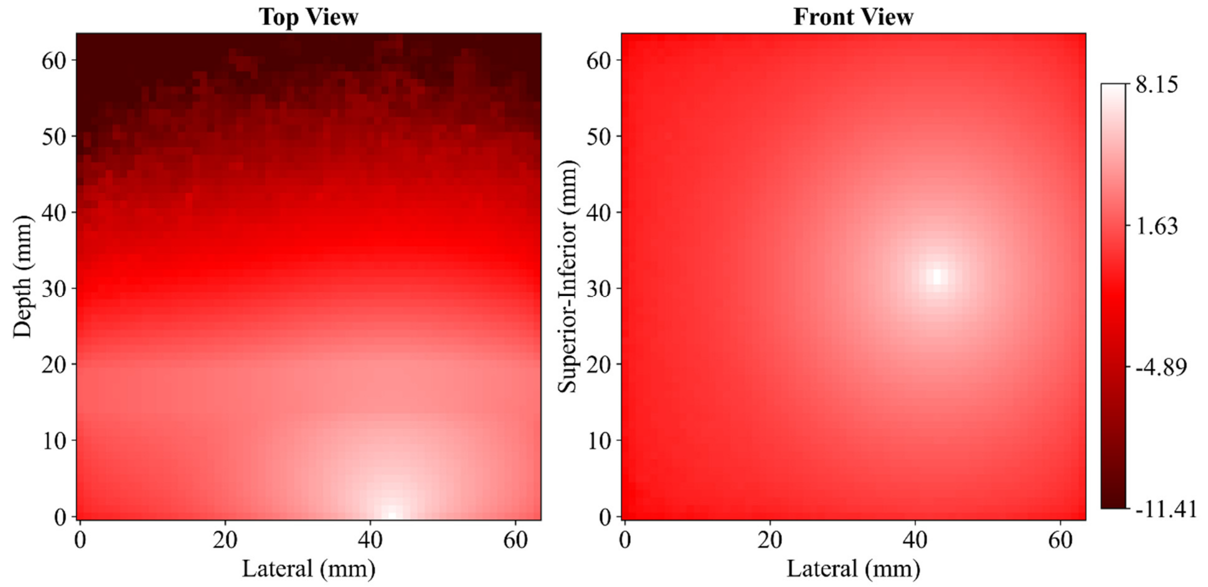


**Figure S14** Spatial fluence color maps for cadaveric head #2 at 23 mm source-detector separation from top view (left) and front view (right). The bright white spots indicate the position of the light source. The color bar represents photon fluence in a logarithmic scale to enhance visibility across a wide dynamic range.

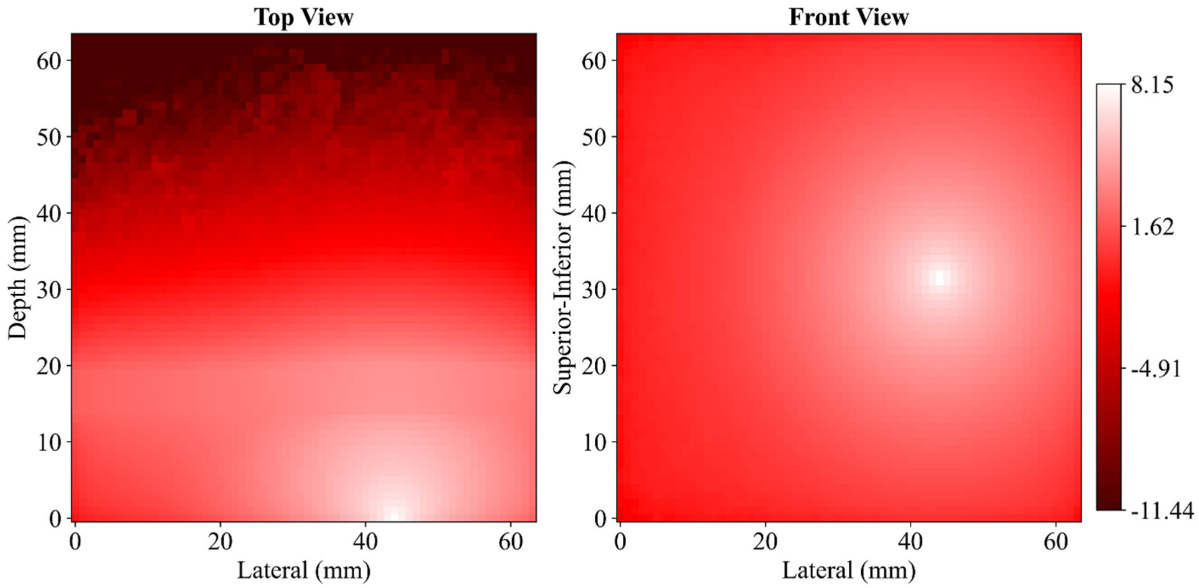


**Figure S15** Spatial fluence color maps for cadaveric head #2 at 25 mm source-detector separation from top view (left) and front view (right). The bright white spots indicate the position of the light source. The color bar represents photon fluence in a logarithmic scale to enhance visibility across a wide dynamic range.

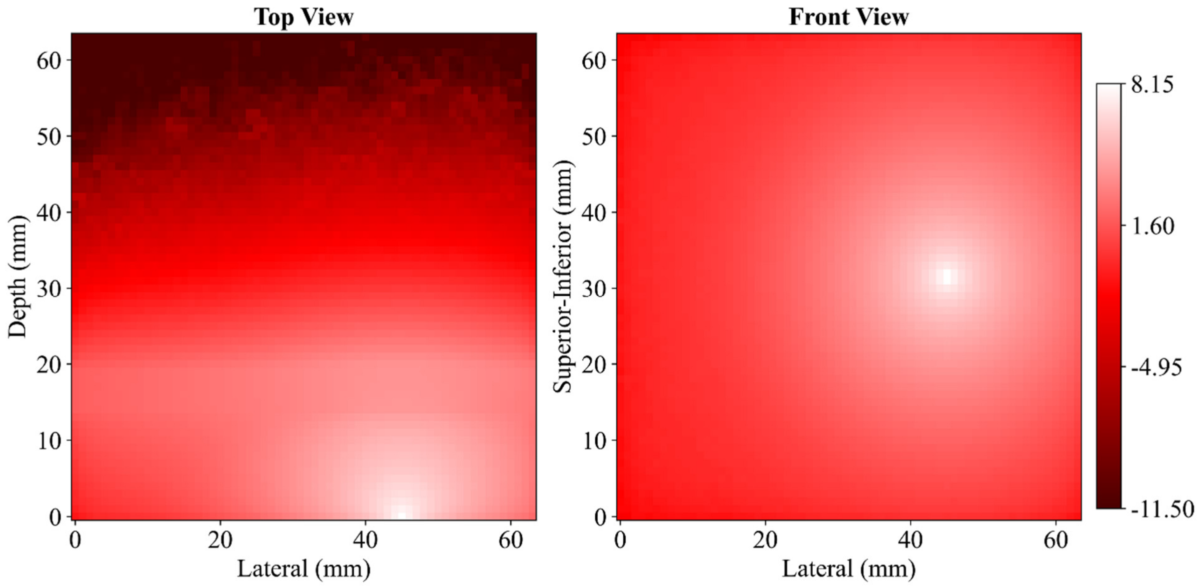


**Figure S16** Spatial fluence color maps for cadaveric head #2 at 27 mm source-detector separation from top view (left) and front view (right). The bright white spots indicate the position of the light source. The color bar represents photon fluence in a logarithmic scale to enhance visibility across a wide dynamic range.

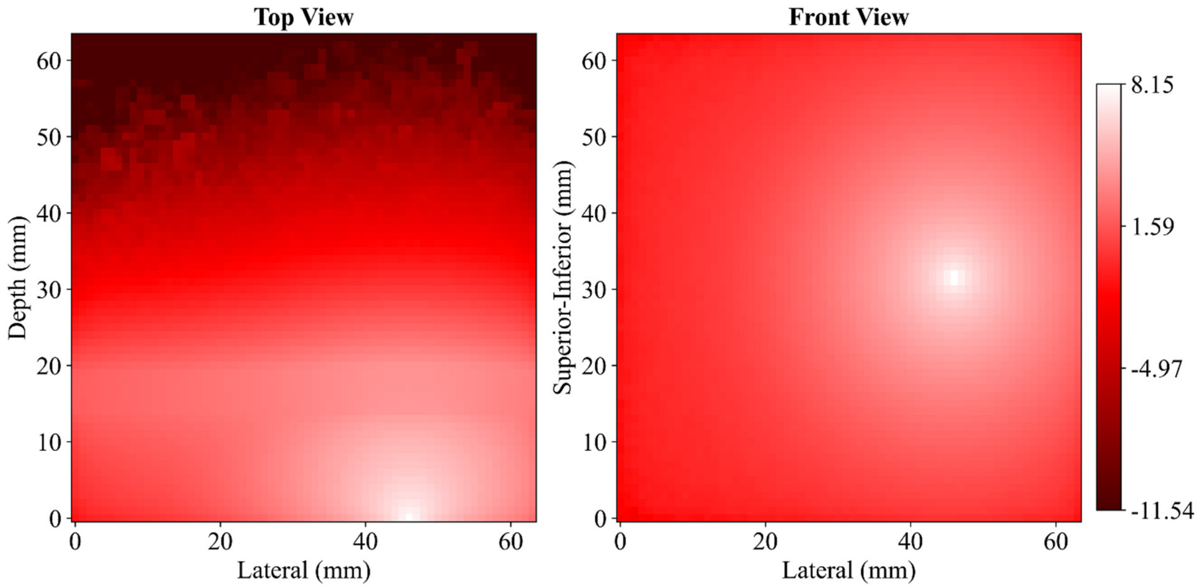


**Figure S17** Spatial fluence color maps for cadaveric head #2 at 29 mm source-detector separation from top view (left) and front view (right). The bright white spots indicate the position of the light source. The color bar represents photon fluence in a logarithmic scale to enhance visibility across a wide dynamic range.

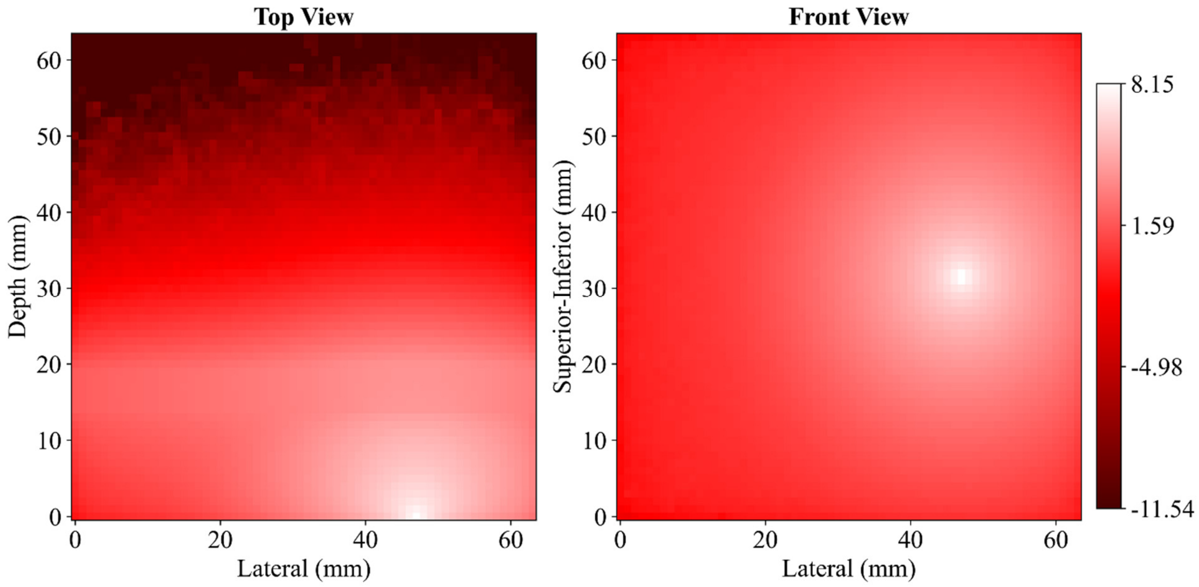


**Figure S18** Spatial fluence color maps for cadaveric head #2 at 31 mm source-detector separation from top view (left) and front view (right). The bright white spots indicate the position of the light source. The color bar represents photon fluence in a logarithmic scale to enhance visibility across a wide dynamic range.

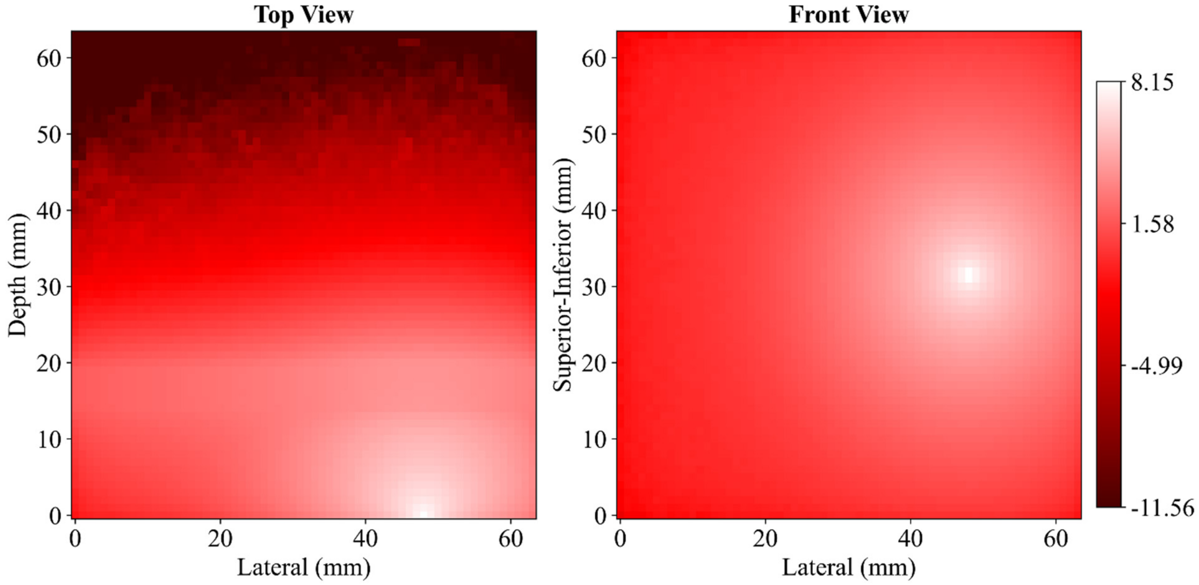


**Figure S19** Spatial fluence color maps for cadaveric head #2 at 33 mm source-detector separation from top view (left) and front view (right). The bright white spots indicate the position of the light source. The color bar represents photon fluence in a logarithmic scale to enhance visibility across a wide dynamic range.

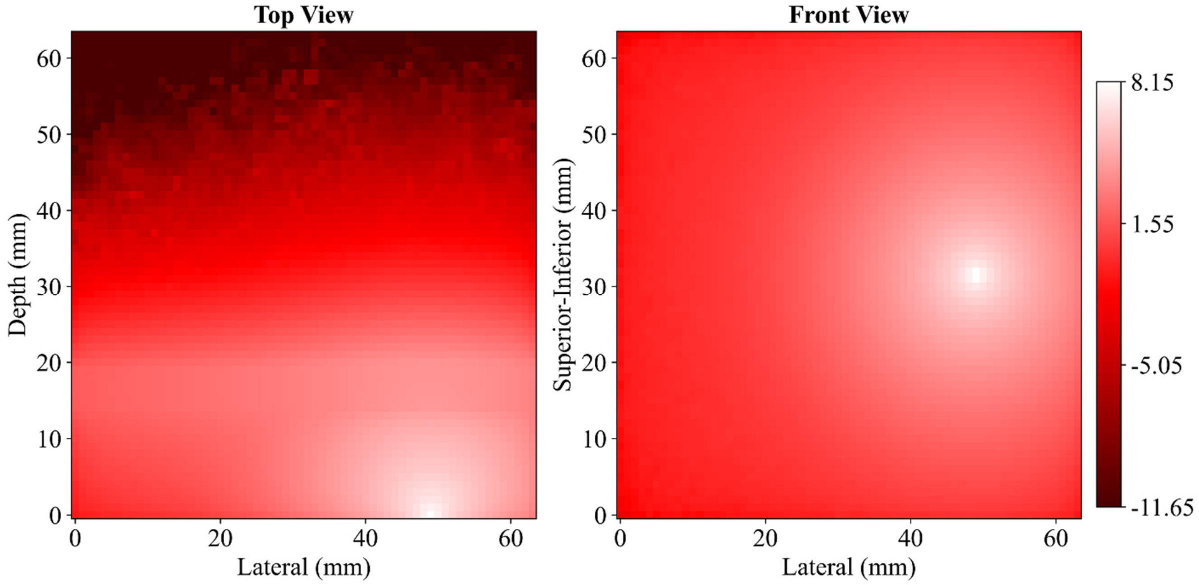


**Figure S20** Spatial fluence color maps for cadaveric head #2 at 35 mm source-detector separation from top view (left) and front view (right). The bright white spots indicate the position of the light source. The color bar represents photon fluence in a logarithmic scale to enhance visibility across a wide dynamic range.

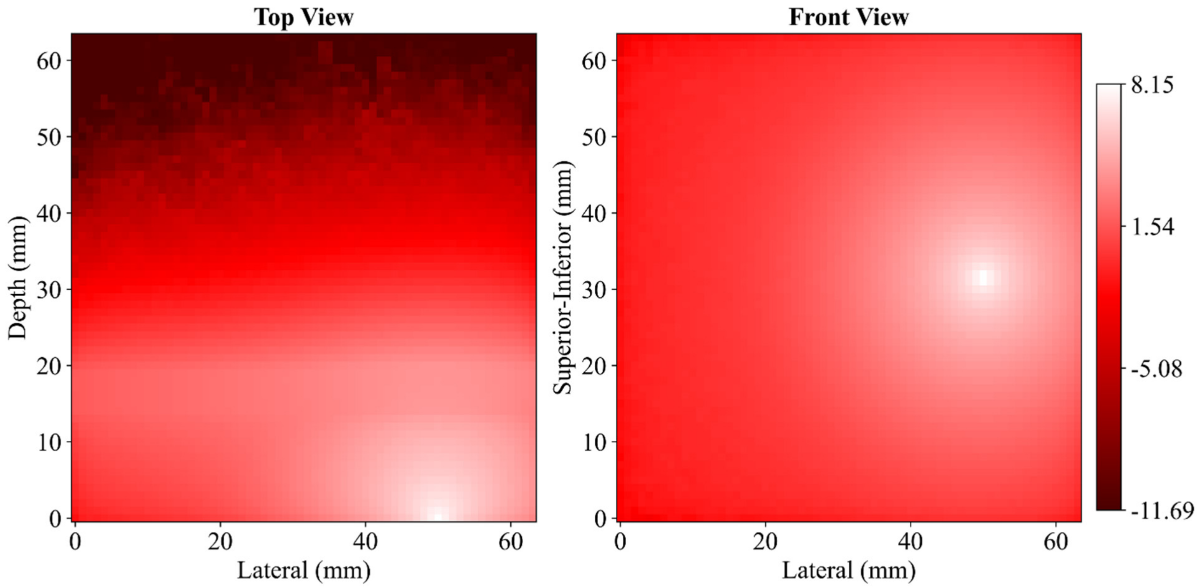


**Figure S21** Spatial fluence color maps for cadaveric head #2 at 37 mm source-detector separation from top view (left) and front view (right). The bright white spots indicate the position of the light source. The color bar represents photon fluence in a logarithmic scale to enhance visibility across a wide dynamic range.

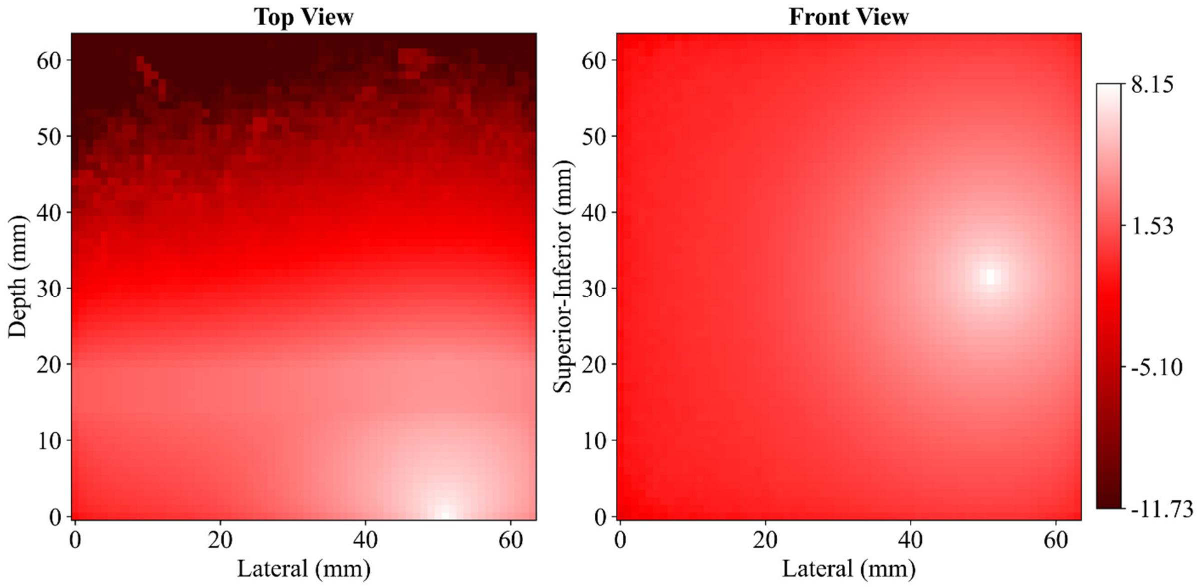


**Figure S22** Spatial fluence color maps for cadaveric head #2 at 39 mm source-detector separation from top view (left) and front view (right). The bright white spots indicate the position of the light source. The color bar represents photon fluence in a logarithmic scale to enhance visibility across a wide dynamic range.

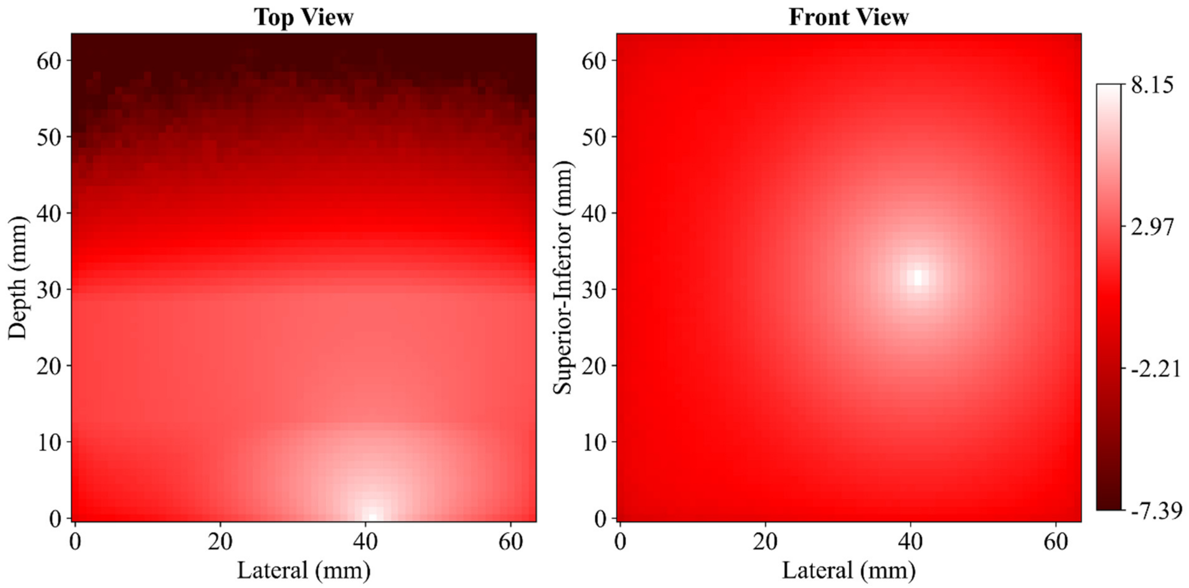


**Figure S23** Spatial fluence color maps for cadaveric head #3 at 19 mm source-detector separation from top view (left) and front view (right). The bright white spots indicate the position of the light source. The color bar represents photon fluence in a logarithmic scale to enhance visibility across a wide dynamic range.

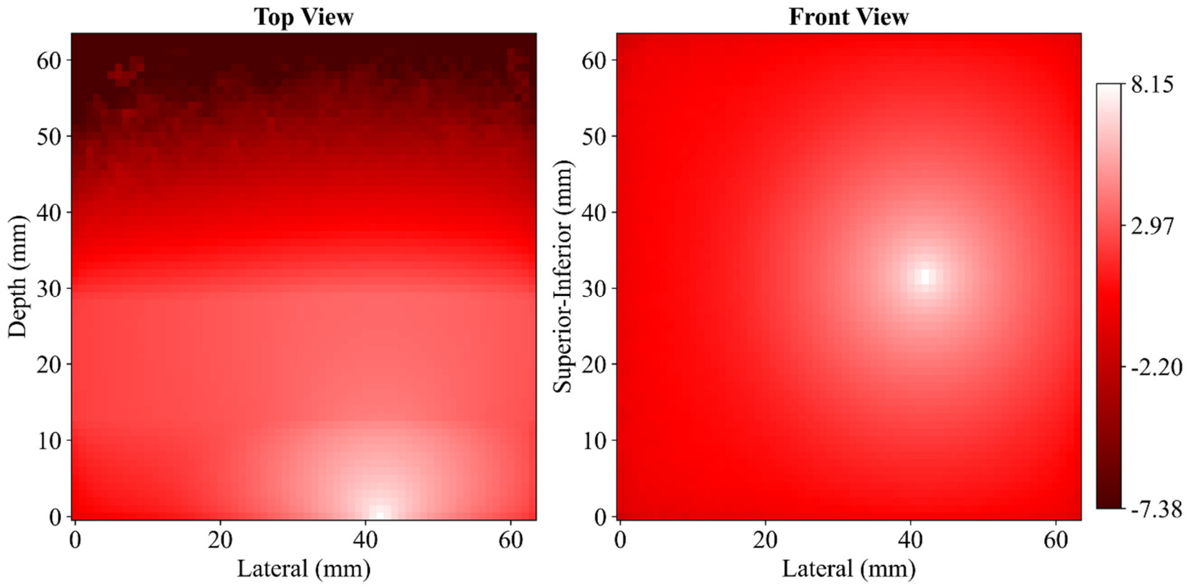


**Figure S24** Spatial fluence color maps for cadaveric head #3 at 21 mm source-detector separation from top view (left) and front view (right). The bright white spots indicate the position of the light source. The color bar represents photon fluence in a logarithmic scale to enhance visibility across a wide dynamic range.

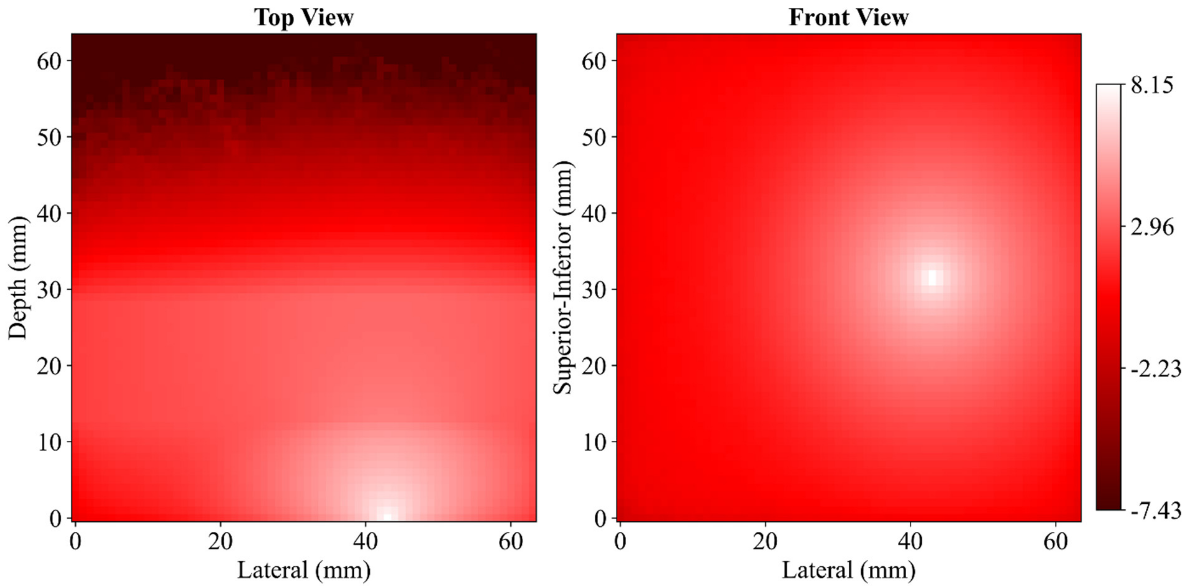


**Figure S25** Spatial fluence color maps for cadaveric head #3 at 23 mm source-detector separation from top view (left) and front view (right). The bright white spots indicate the position of the light source. The color bar represents photon fluence in a logarithmic scale to enhance visibility across a wide dynamic range.

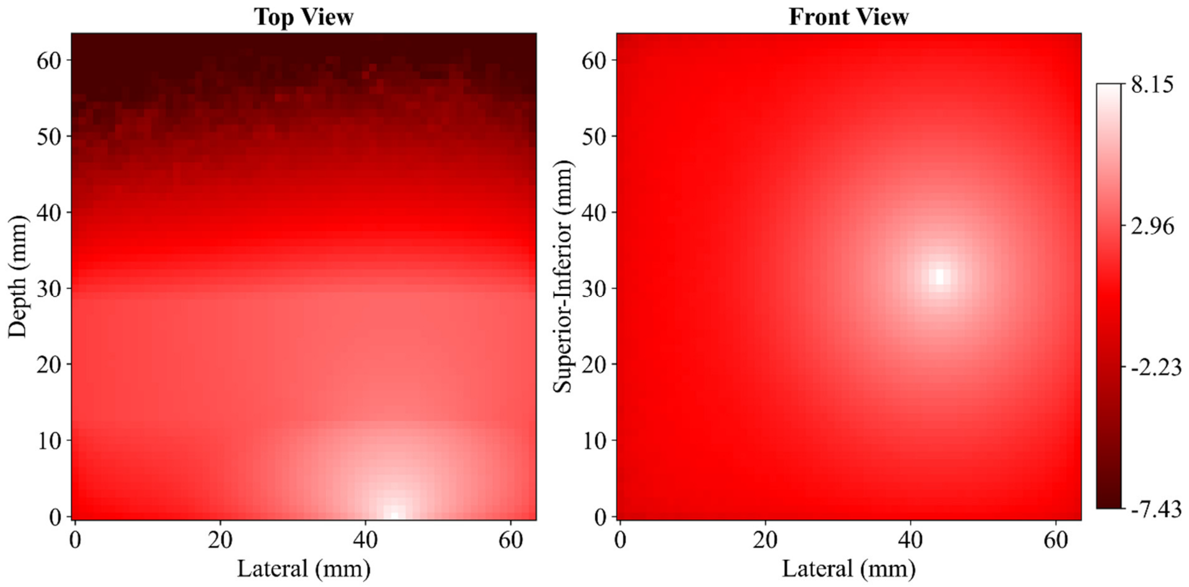


**Figure S26** Spatial fluence color maps for cadaveric head #3 at 25 mm source-detector separation from top view (left) and front view (right). The bright white spots indicate the position of the light source. The color bar represents photon fluence in a logarithmic scale to enhance visibility across a wide dynamic range.

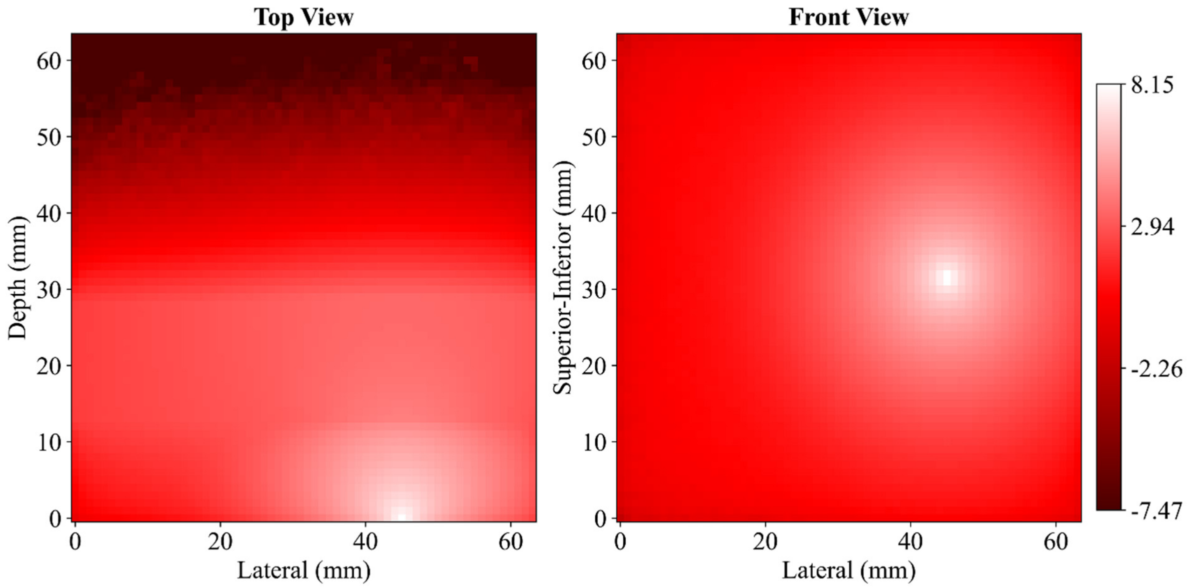


**Figure S27** Spatial fluence color maps for cadaveric head #3 at 27 mm source-detector separation from top view (left) and front view (right). The bright white spots indicate the position of the light source. The color bar represents photon fluence in a logarithmic scale to enhance visibility across a wide dynamic range.

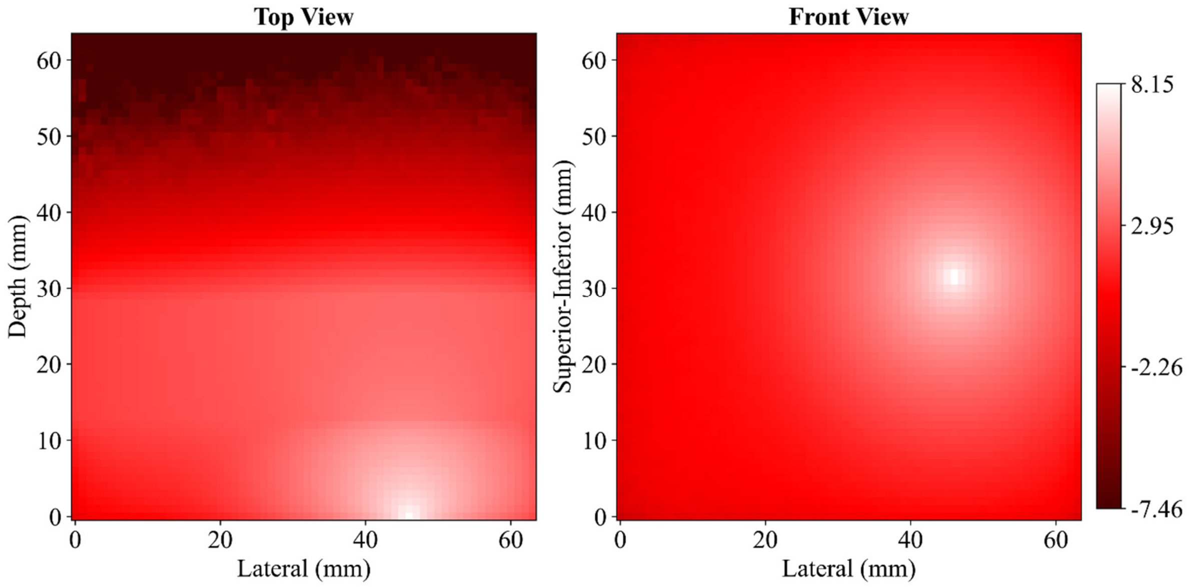


**Figure S28** Spatial fluence color maps for cadaveric head #3 at 29 mm source-detector separation from top view (left) and front view (right). The bright white spots indicate the position of the light source. The color bar represents photon fluence in a logarithmic scale to enhance visibility across a wide dynamic range.

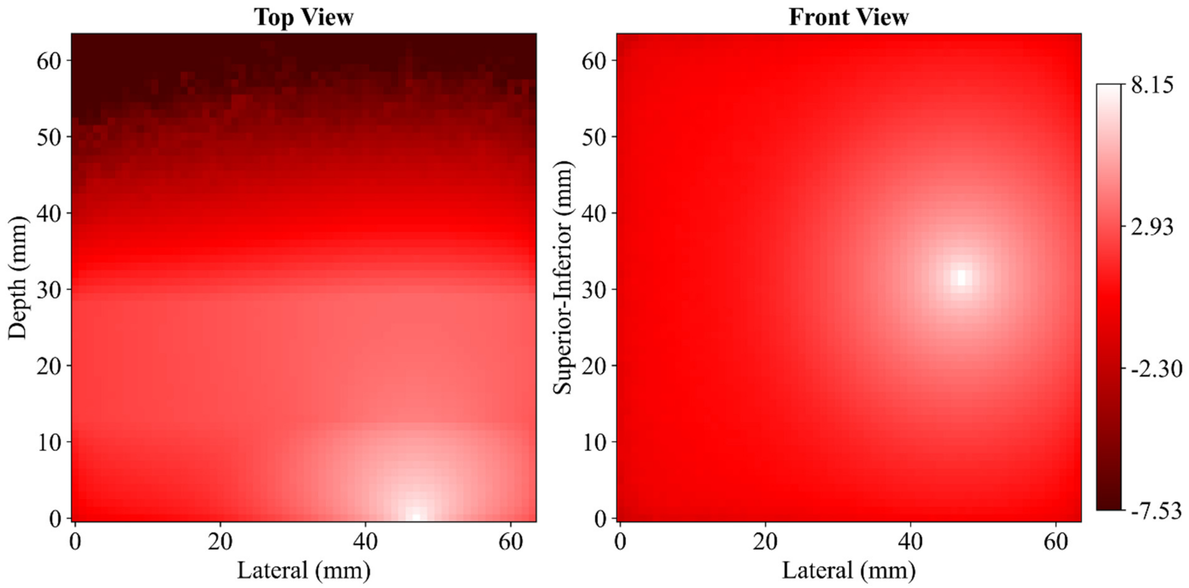


**Figure S29** Spatial fluence color maps for cadaveric head #3 at 31 mm source-detector separation from top view (left) and front view (right). The bright white spots indicate the position of the light source. The color bar represents photon fluence in a logarithmic scale to enhance visibility across a wide dynamic range.

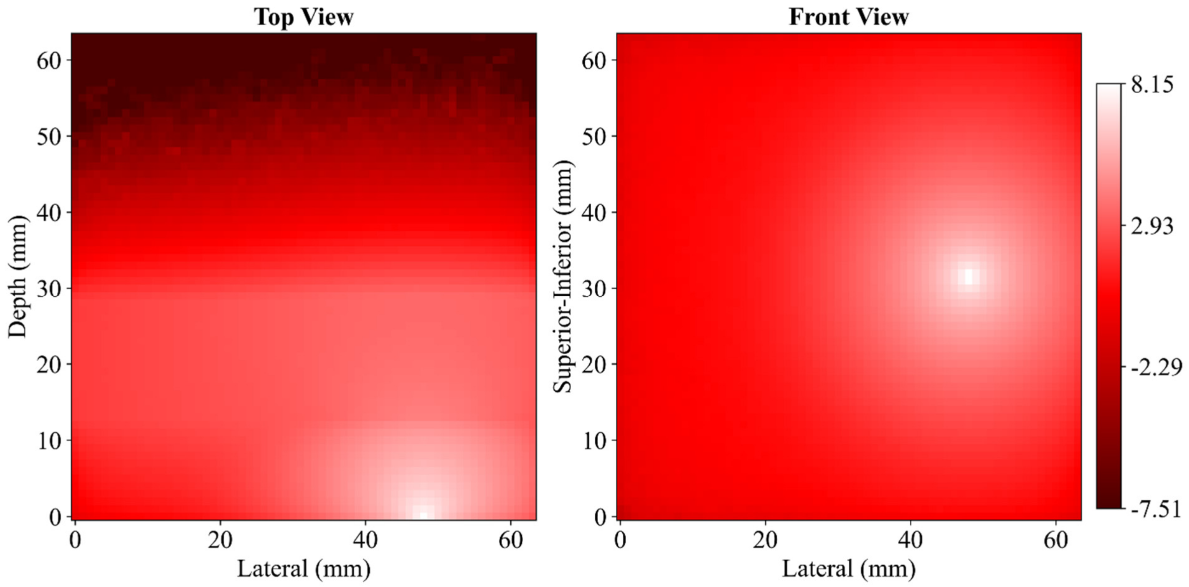


**Figure S30** Spatial fluence color maps for cadaveric head #3 at 33 mm source-detector separation from top view (left) and front view (right). The bright white spots indicate the position of the light source. The color bar represents photon fluence in a logarithmic scale to enhance visibility across a wide dynamic range.

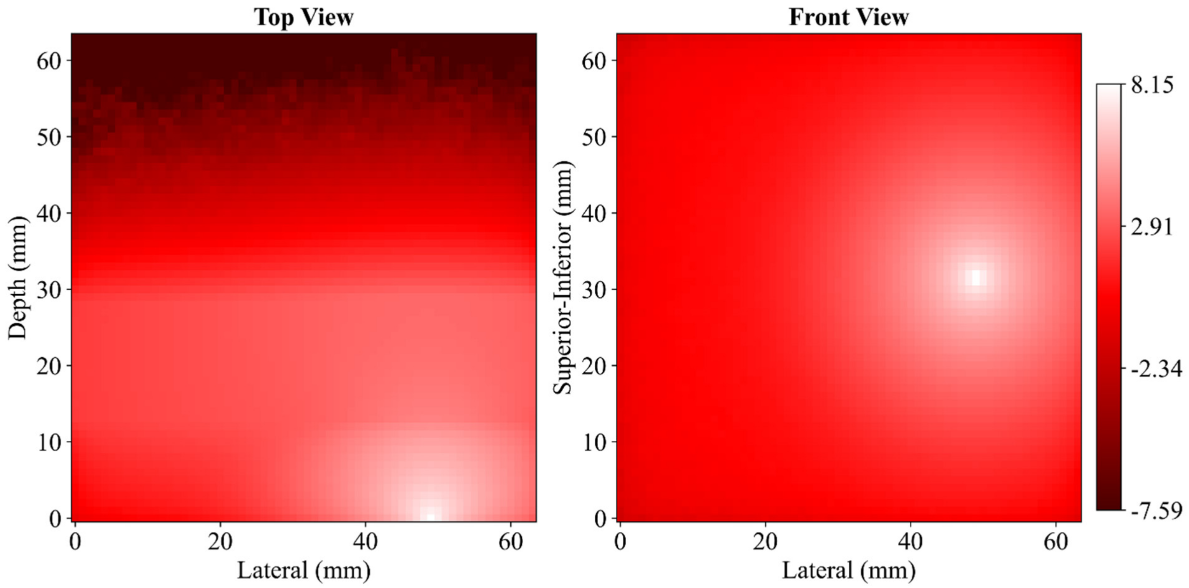


**Figure S31** Spatial fluence color maps for cadaveric head #3 at 35 mm source-detector separation from top view (left) and front view (right). The bright white spots indicate the position of the light source. The color bar represents photon fluence in a logarithmic scale to enhance visibility across a wide dynamic range.

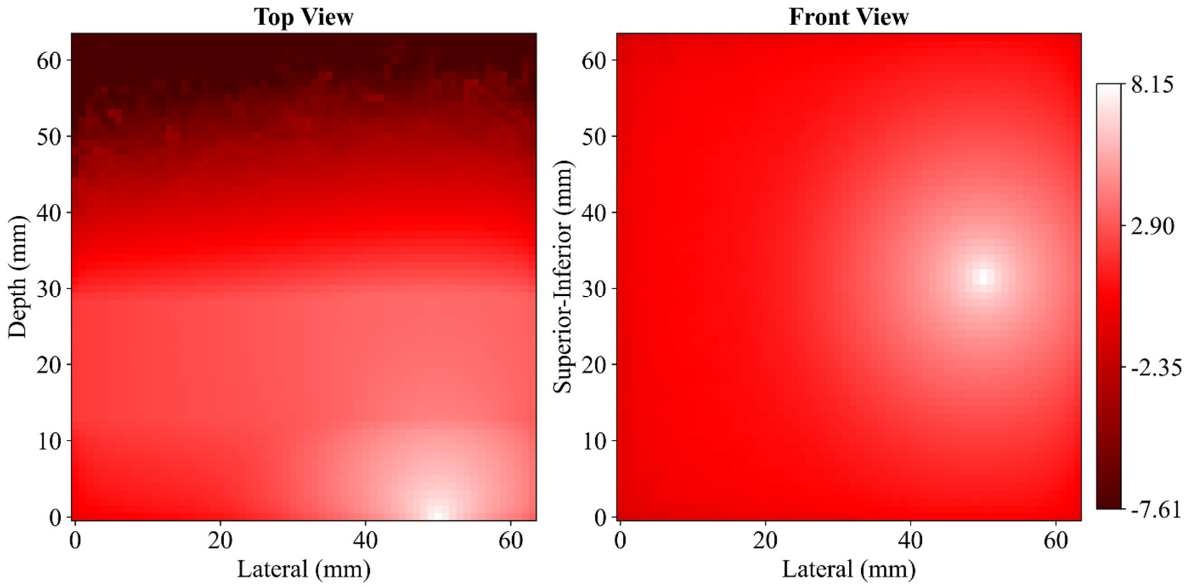


**Figure S32** Spatial fluence color maps for cadaveric head #3 at 37 mm source-detector separation from top view (left) and front view (right). The bright white spots indicate the position of the light source. The color bar represents photon fluence in a logarithmic scale to enhance visibility across a wide dynamic range.

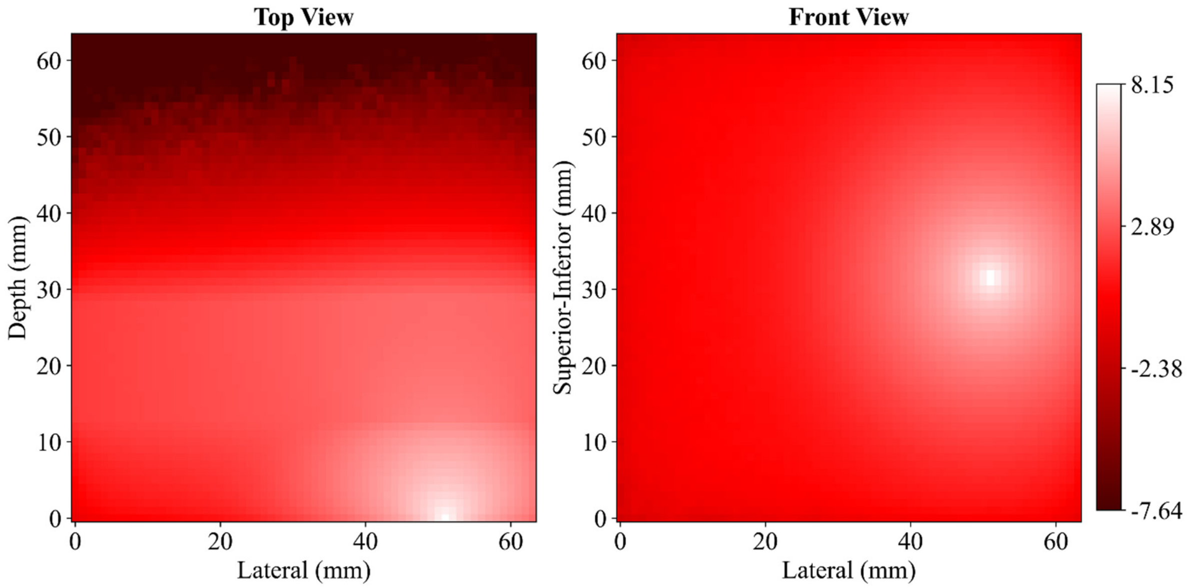


**Figure S33** Spatial fluence color maps for cadaveric head #3 at 39 mm source-detector separation from top view (left) and front view (right). The bright white spots indicate the position of the light source. The color bar represents photon fluence in a logarithmic scale to enhance visibility across a wide dynamic range.

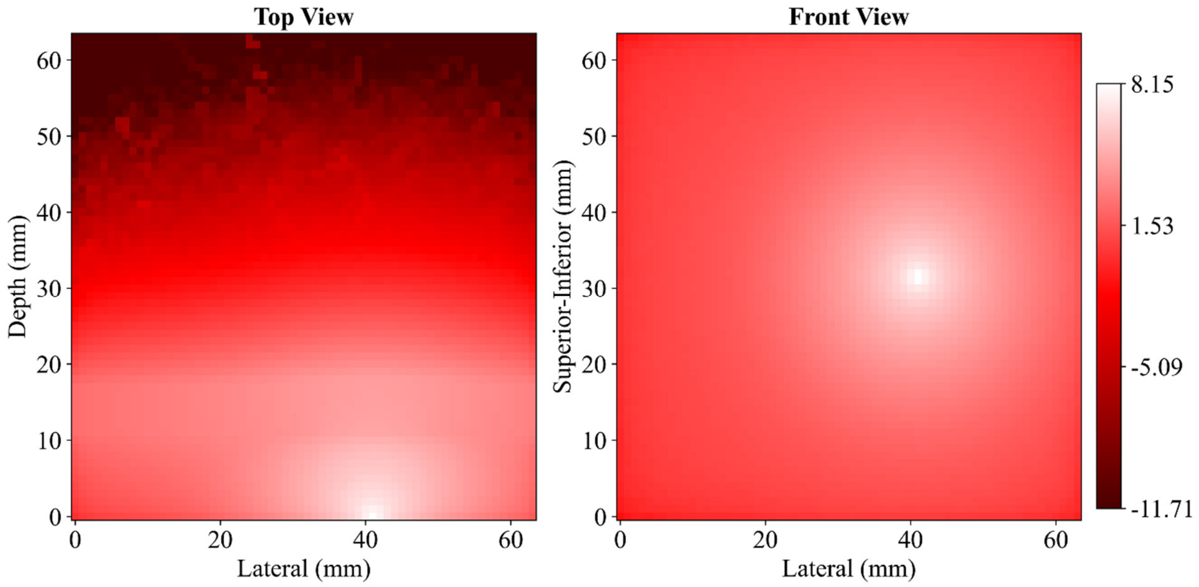


**Figure S34** Spatial fluence color maps for cadaveric head #4 at 19 mm source-detector separation from top view (left) and front view (right). The bright white spots indicate the position of the light source. The color bar represents photon fluence in a logarithmic scale to enhance visibility across a wide dynamic range.

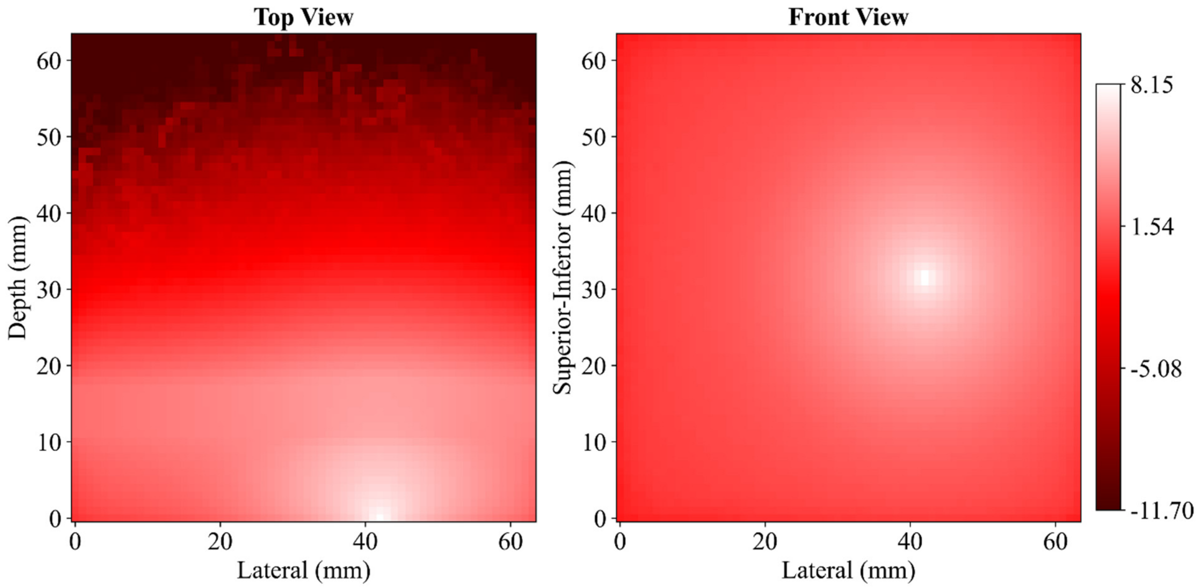


**Figure S35** Spatial fluence color maps for cadaveric head #4 at 21 mm source-detector separation from top view (left) and front view (right). The bright white spots indicate the position of the light source. The color bar represents photon fluence in a logarithmic scale to enhance visibility across a wide dynamic range.

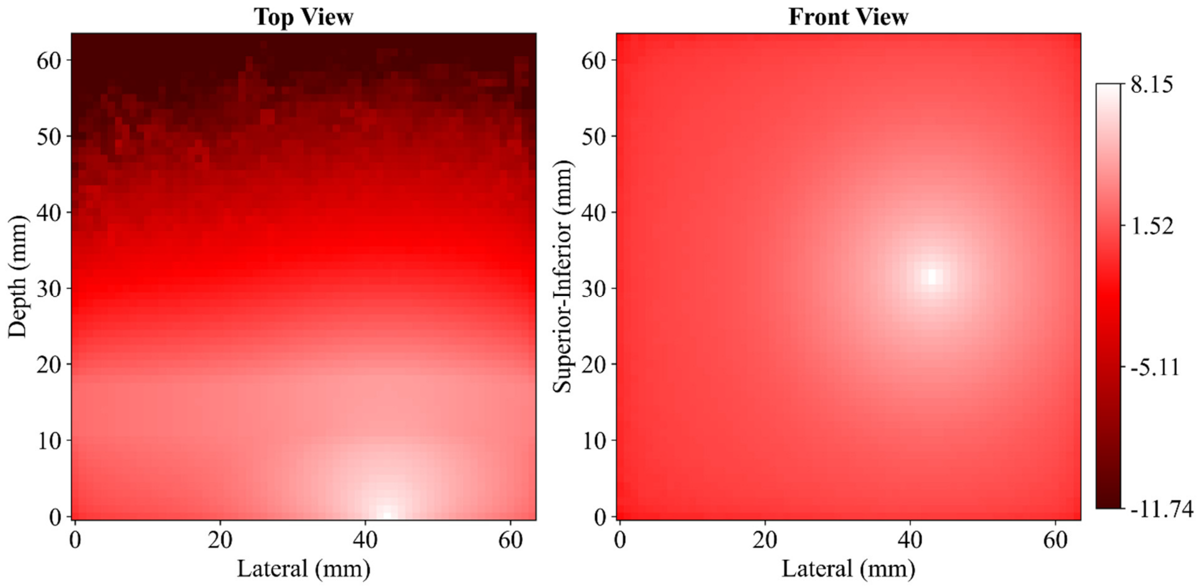


**Figure S36** Spatial fluence color maps for cadaveric head #4 at 23 mm source-detector separation from top view (left) and front view (right). The bright white spots indicate the position of the light source. The color bar represents photon fluence in a logarithmic scale to enhance visibility across a wide dynamic range.

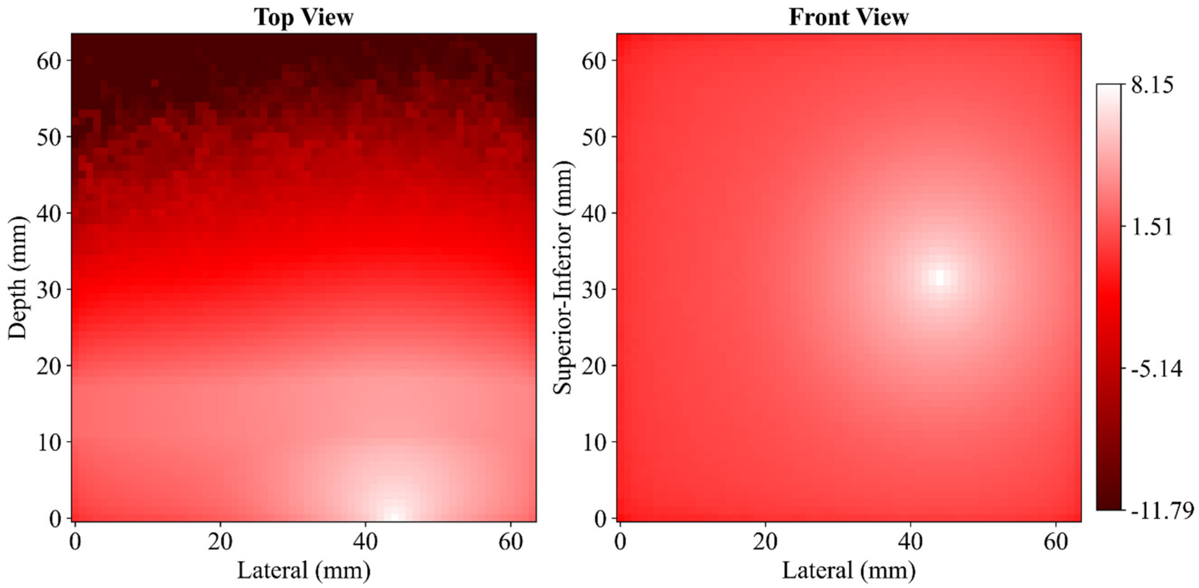


**Figure S37** Spatial fluence color maps for cadaveric head #4 at 25 mm source-detector separation from top view (left) and front view (right). The bright white spots indicate the position of the light source. The color bar represents photon fluence in a logarithmic scale to enhance visibility across a wide dynamic range.

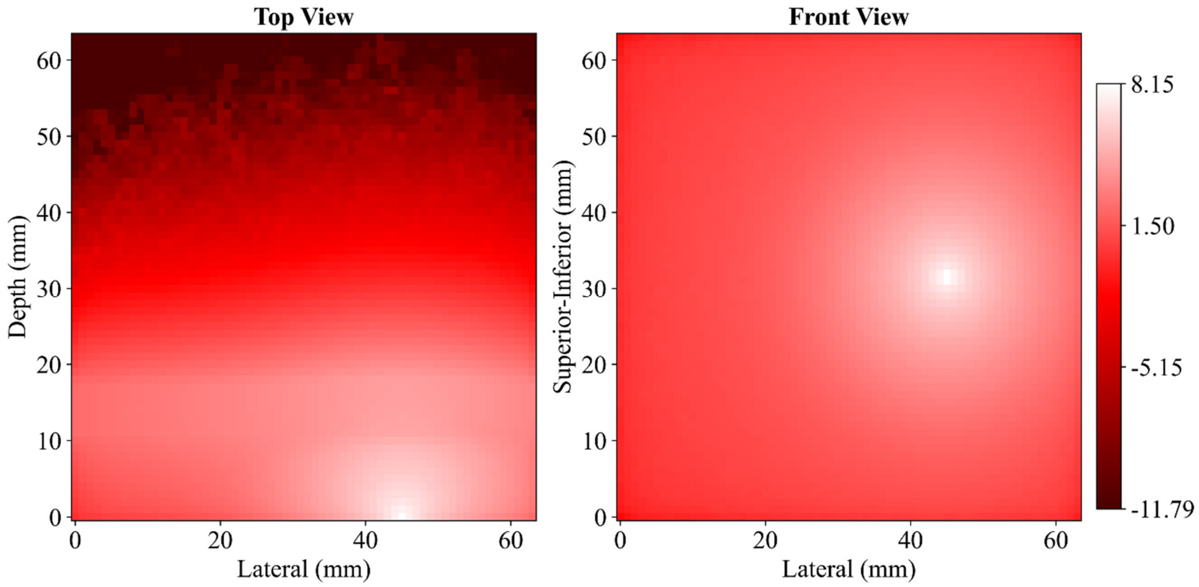


**Figure S38** Spatial fluence color maps for cadaveric head #4 at 27 mm source-detector separation from top view (left) and front view (right). The bright white spots indicate the position of the light source. The color bar represents photon fluence in a logarithmic scale to enhance visibility across a wide dynamic range.

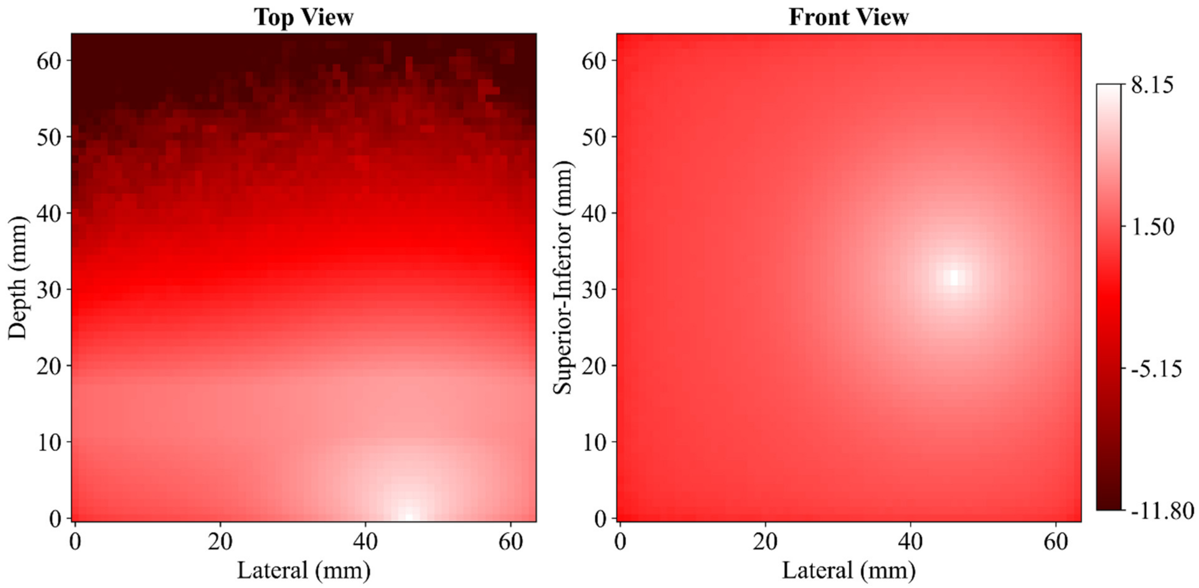


**Figure S39** Spatial fluence color maps for cadaveric head #4 at 29 mm source-detector separation from top view (left) and front view (right). The bright white spots indicate the position of the light source. The color bar represents photon fluence in a logarithmic scale to enhance visibility across a wide dynamic range.

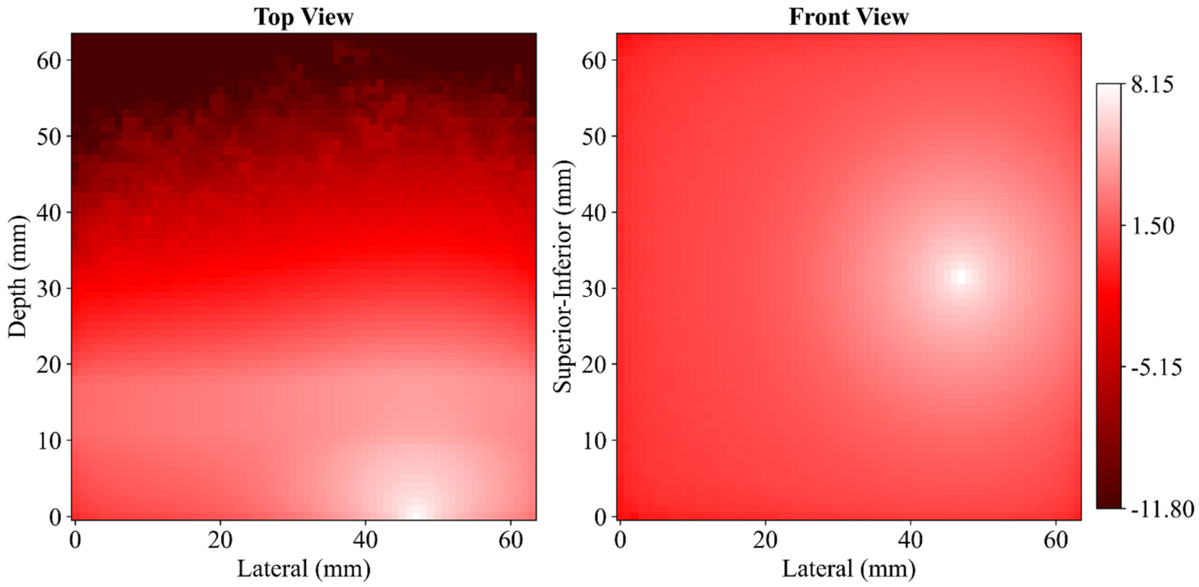


**Figure S40** Spatial fluence color maps for cadaveric head #4 at 31 mm source-detector separation from top view (left) and front view (right). The bright white spots indicate the position of the light source. The color bar represents photon fluence in a logarithmic scale to enhance visibility across a wide dynamic range.

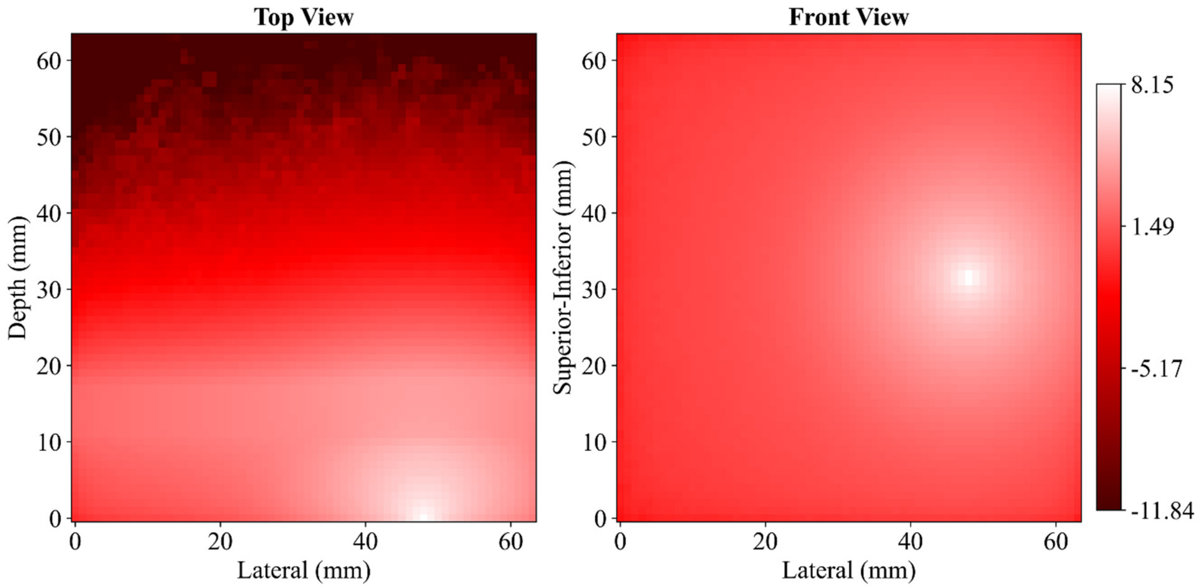


**Figure S41** Spatial fluence color maps for cadaveric head #4 at 33 mm source-detector separation from top view (left) and front view (right). The bright white spots indicate the position of the light source. The color bar represents photon fluence in a logarithmic scale to enhance visibility across a wide dynamic range.

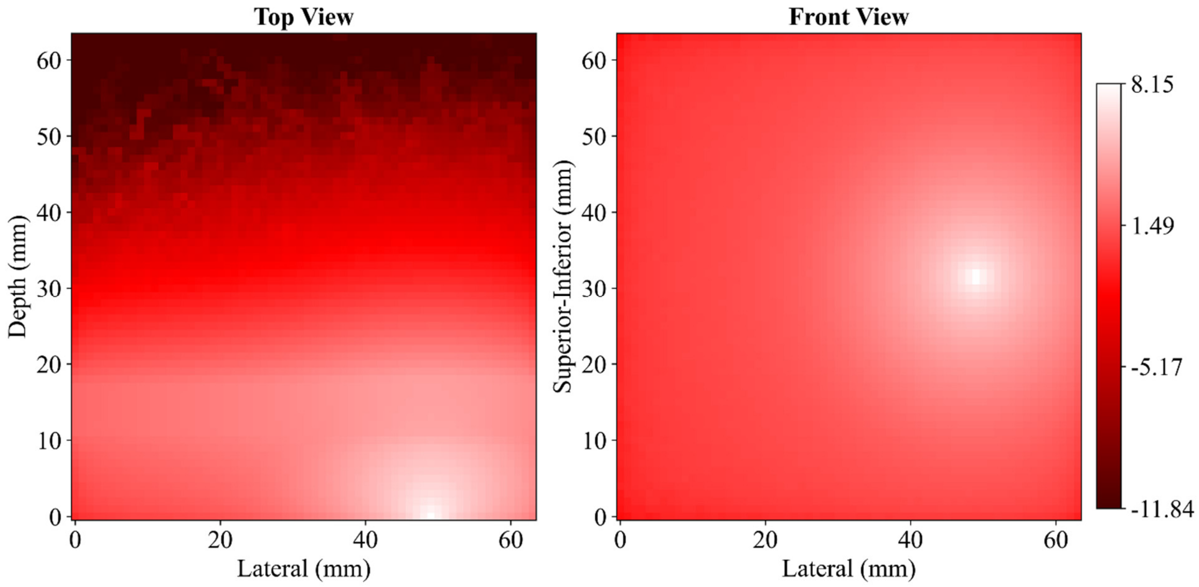


**Figure S42** Spatial fluence color maps for cadaveric head #4 at 35 mm source-detector separation from top view (left) and front view (right). The bright white spots indicate the position of the light source. The color bar represents photon fluence in a logarithmic scale to enhance visibility across a wide dynamic range.

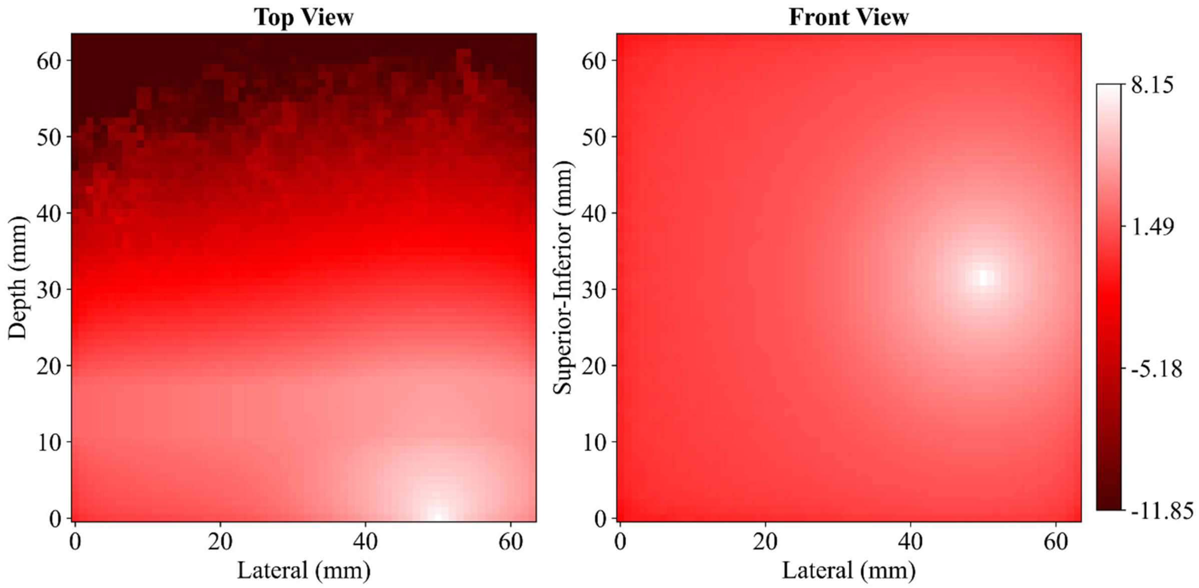


**Figure S43** Spatial fluence color maps for cadaveric head #4 at 37 mm source-detector separation from top view (left) and front view (right). The bright white spots indicate the position of the light source. The color bar represents photon fluence in a logarithmic scale to enhance visibility across a wide dynamic range.

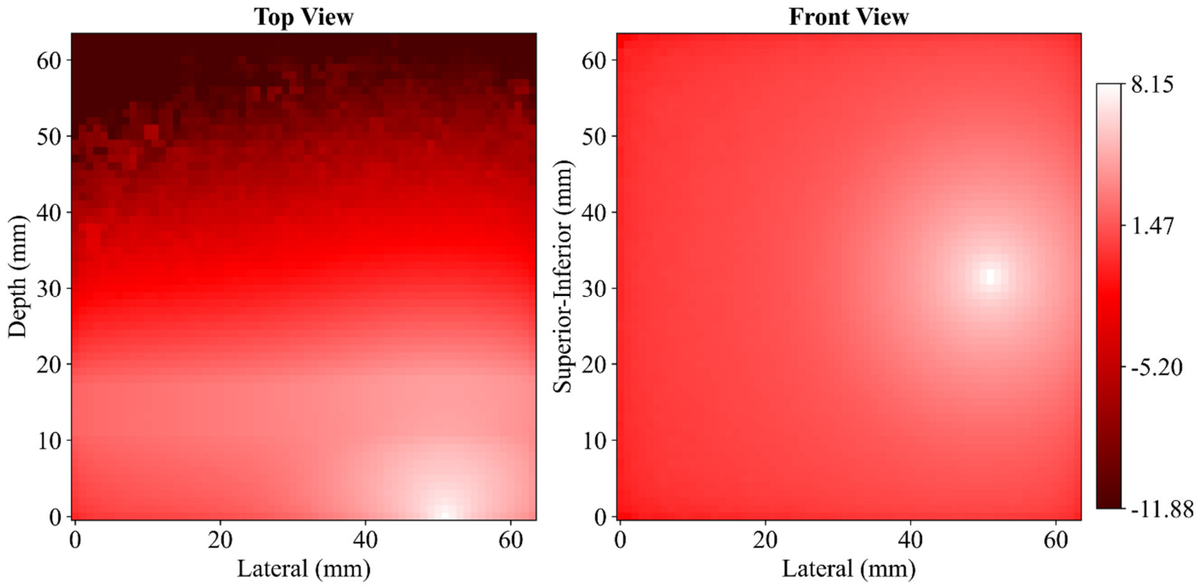


**Figure S44** Spatial fluence color maps for cadaveric head #4 at 39 mm source-detector separation from top view (left) and front view (right). The bright white spots indicate the position of the light source. The color bar represents photon fluence in a logarithmic scale to enhance visibility across a wide dynamic range.

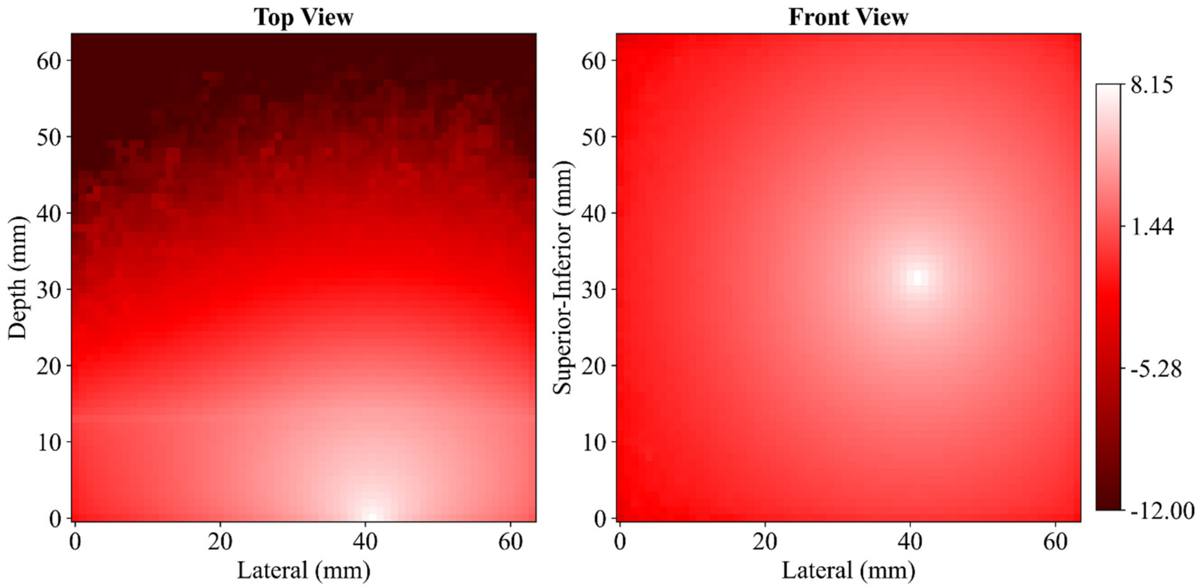


**Figure S45** Spatial fluence color maps for cadaveric head #5 at 19 mm source-detector separation from top view (left) and front view (right). The bright white spots indicate the position of the light source. The color bar represents photon fluence in a logarithmic scale to enhance visibility across a wide dynamic range.

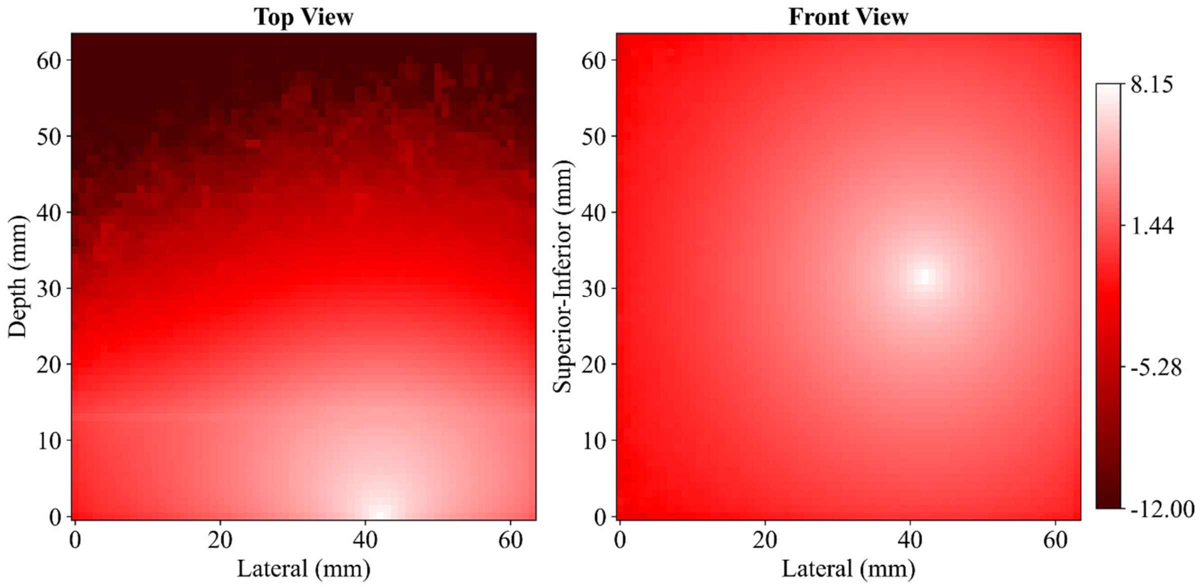


**Figure S46** Spatial fluence color maps for cadaveric head #5 at 21 mm source-detector separation from top view (left) and front view (right). The bright white spots indicate the position of the light source. The color bar represents photon fluence in a logarithmic scale to enhance visibility across a wide dynamic range.

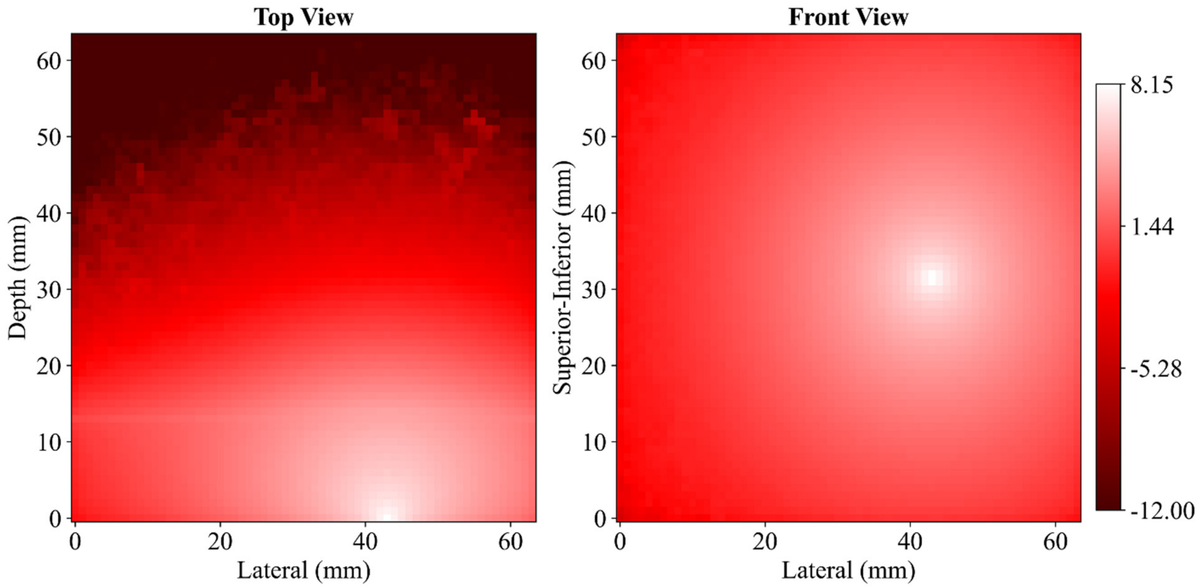


**Figure S47** Spatial fluence color maps for cadaveric head #5 at 23 mm source-detector separation from top view (left) and front view (right). The bright white spots indicate the position of the light source. The color bar represents photon fluence in a logarithmic scale to enhance visibility across a wide dynamic range.

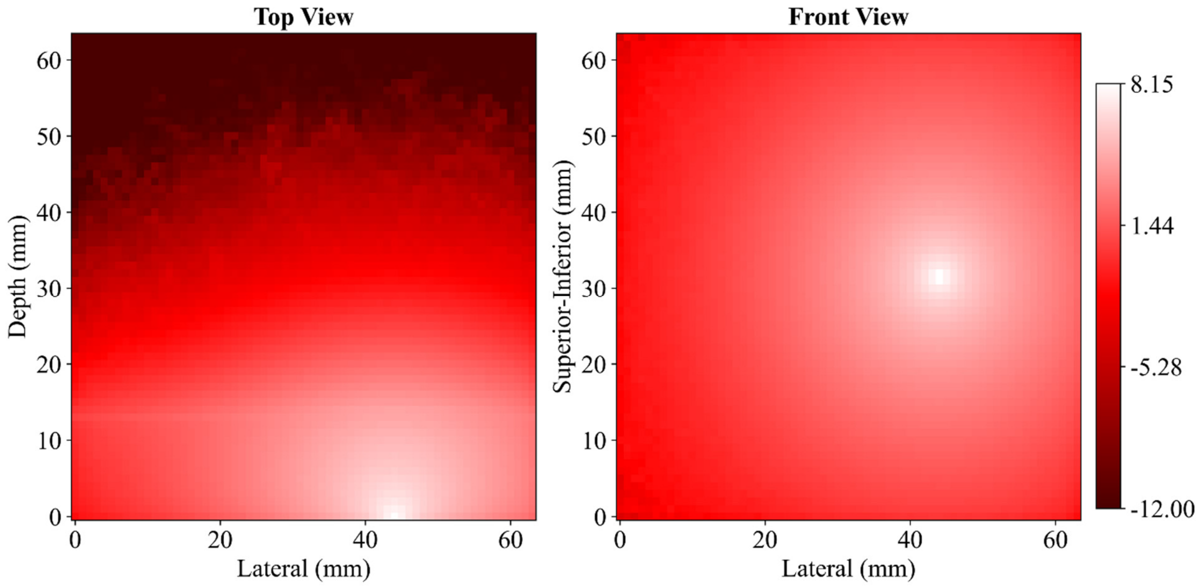


**Figure S48** Spatial fluence color maps for cadaveric head #5 at 25 mm source-detector separation from top view (left) and front view (right). The bright white spots indicate the position of the light source. The color bar represents photon fluence in a logarithmic scale to enhance visibility across a wide dynamic range.

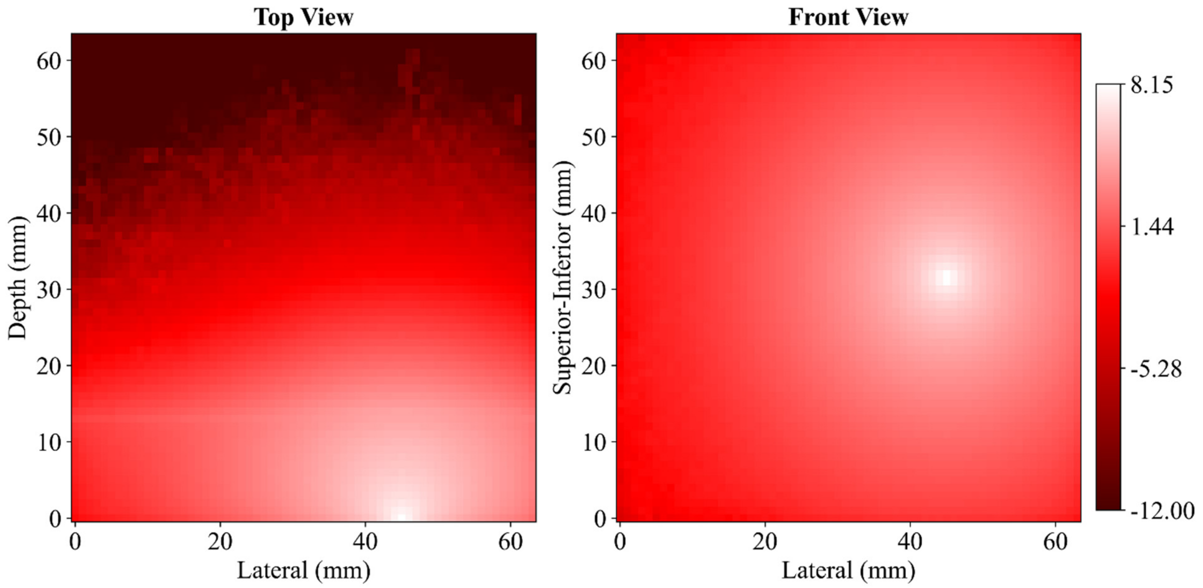


**Figure S49** Spatial fluence color maps for cadaveric head #5 at 27 mm source-detector separation from top view (left) and front view (right). The bright white spots indicate the position of the light source. The color bar represents photon fluence in a logarithmic scale to enhance visibility across a wide dynamic range.

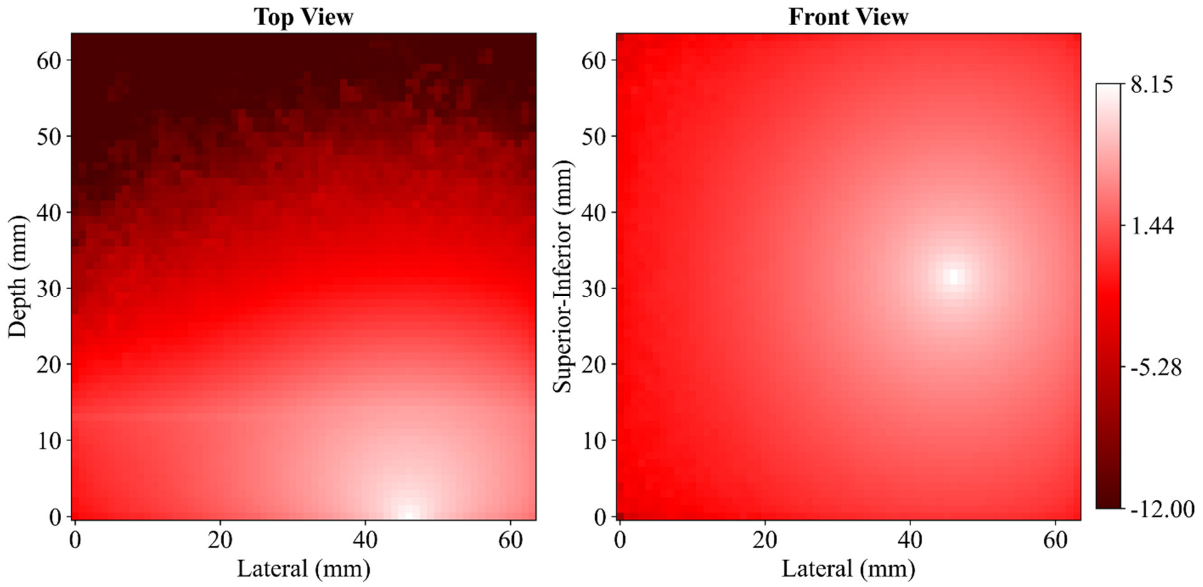


**Figure S50** Spatial fluence color maps for cadaveric head #5 at 29 mm source-detector separation from top view (left) and front view (right). The bright white spots indicate the position of the light source. The color bar represents photon fluence in a logarithmic scale to enhance visibility across a wide dynamic range.

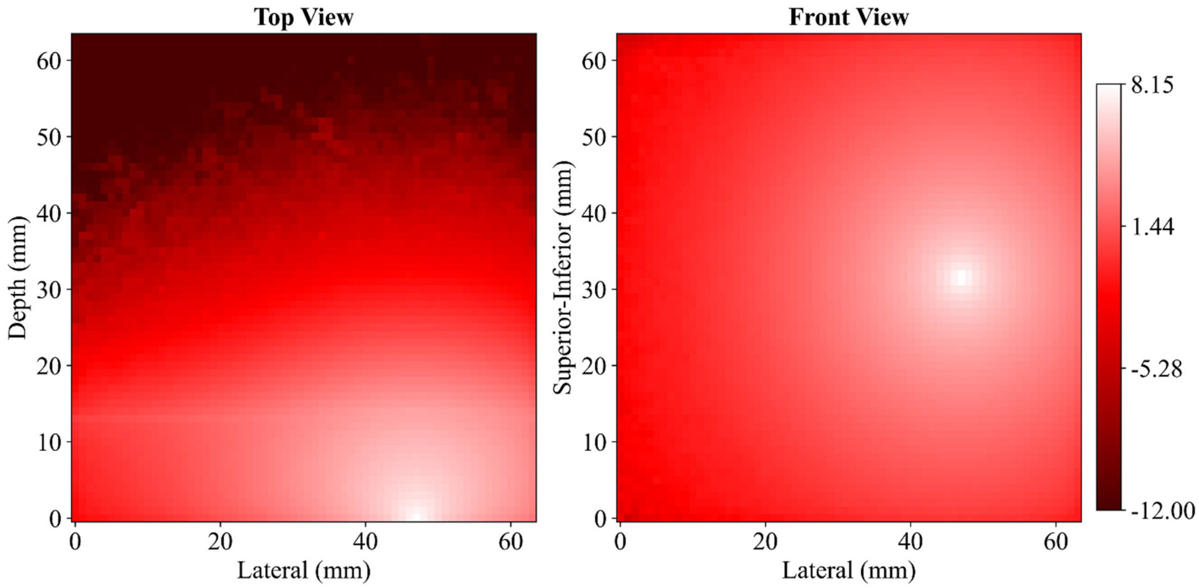


**Figure S51** Spatial fluence color maps for cadaveric head #5 at 31 mm source-detector separation from top view (left) and front view (right). The bright white spots indicate the position of the light source. The color bar represents photon fluence in a logarithmic scale to enhance visibility across a wide dynamic range.

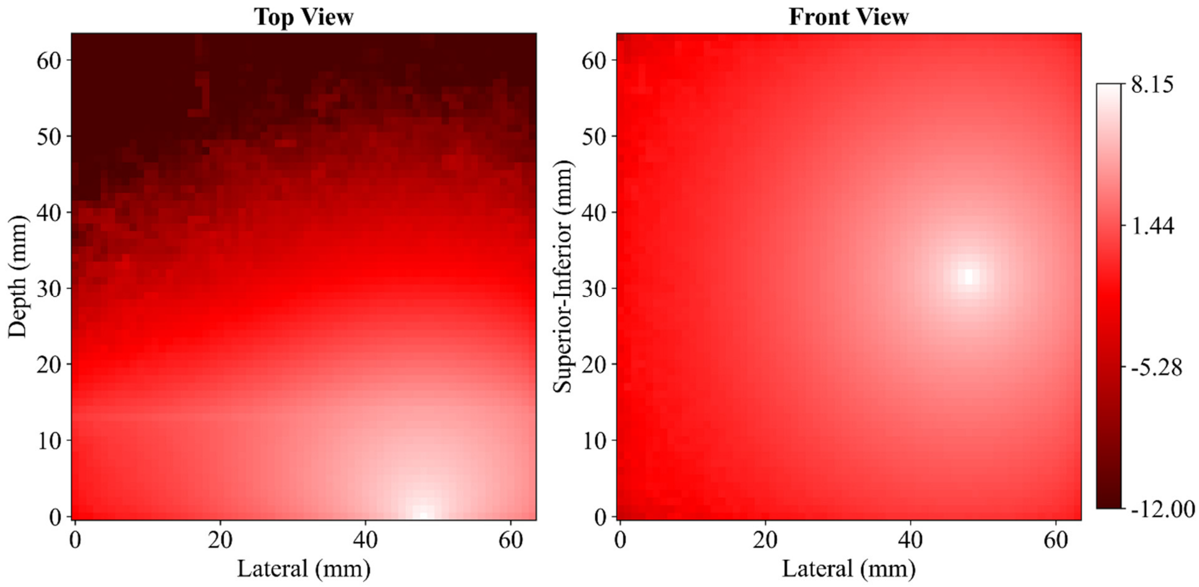


**Figure S52** Spatial fluence color maps for cadaveric head #5 at 33 mm source-detector separation from top view (left) and front view (right). The bright white spots indicate the position of the light source. The color bar represents photon fluence in a logarithmic scale to enhance visibility across a wide dynamic range.

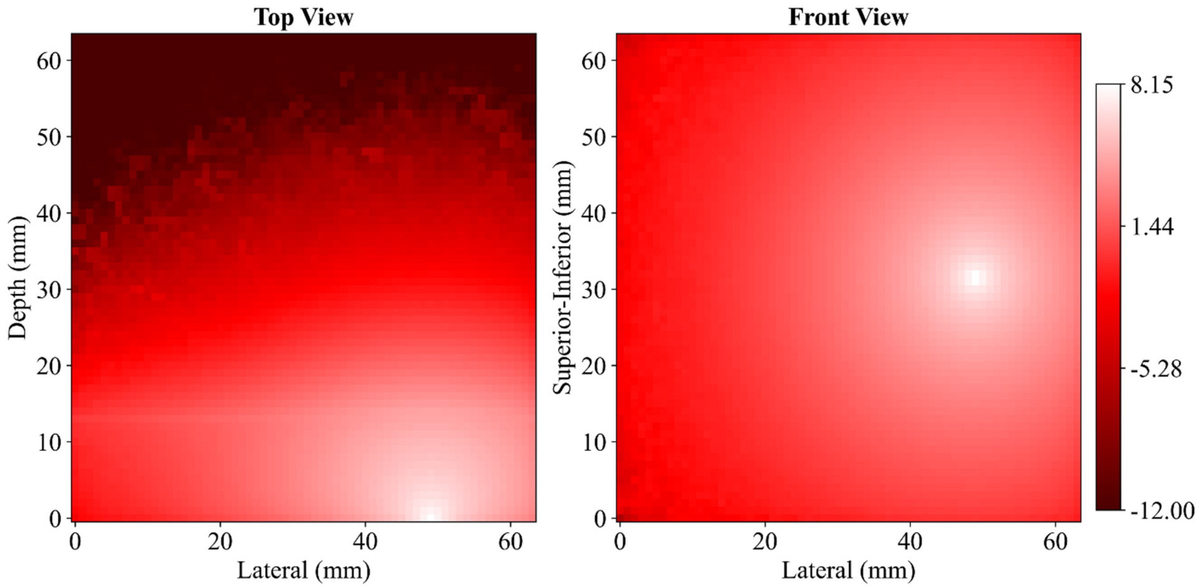


**Figure S53** Spatial fluence color maps for cadaveric head #5 at 35 mm source-detector separation from top view (left) and front view (right). The bright white spots indicate the position of the light source. The color bar represents photon fluence in a logarithmic scale to enhance visibility across a wide dynamic range.

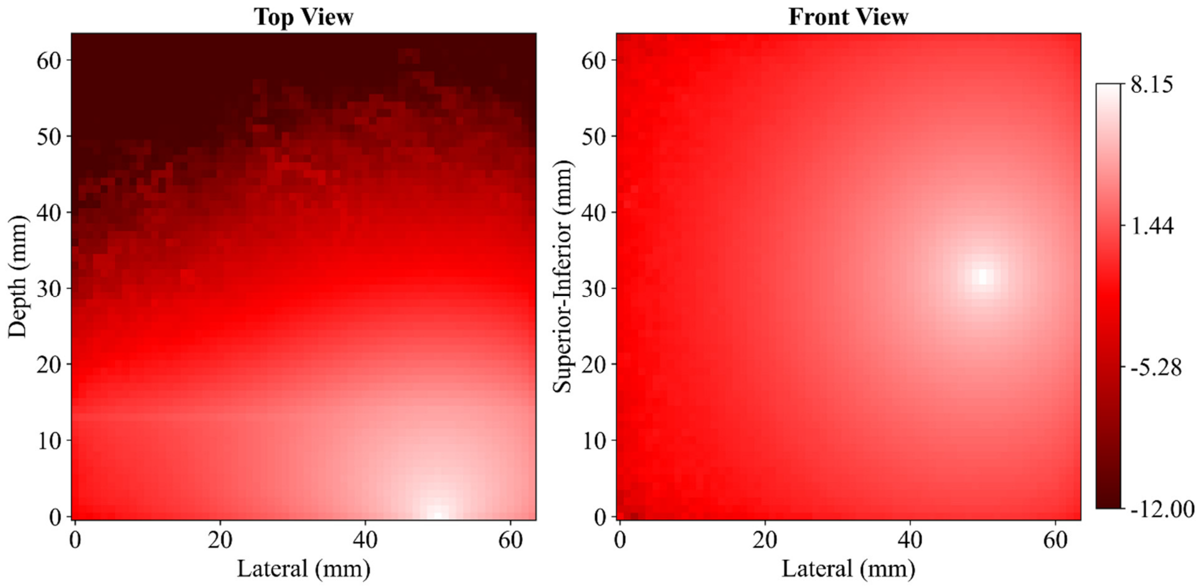


**Figure S54** Spatial fluence color maps for cadaveric head #5 at 37 mm source-detector separation from top view (left) and front view (right). The bright white spots indicate the position of the light source. The color bar represents photon fluence in a logarithmic scale to enhance visibility across a wide dynamic range.

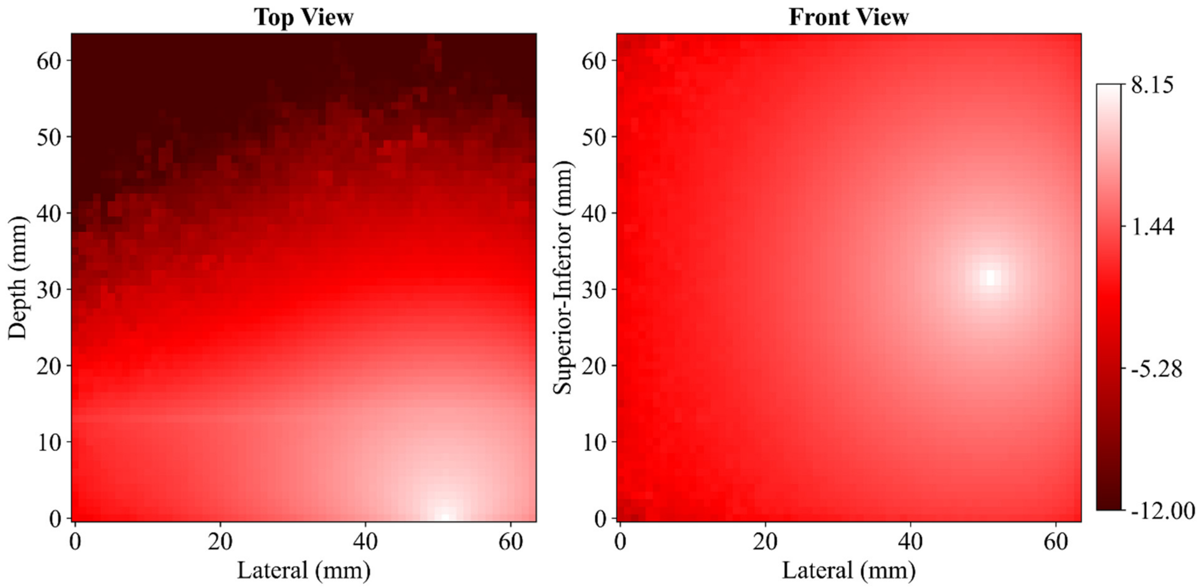


**Figure S55** Spatial fluence color maps for cadaveric head #5 at 39 mm source-detector separation from top view (left) and front view (right). The bright white spots indicate the position of the light source. The color bar represents photon fluence in a logarithmic scale to enhance visibility across a wide dynamic range.

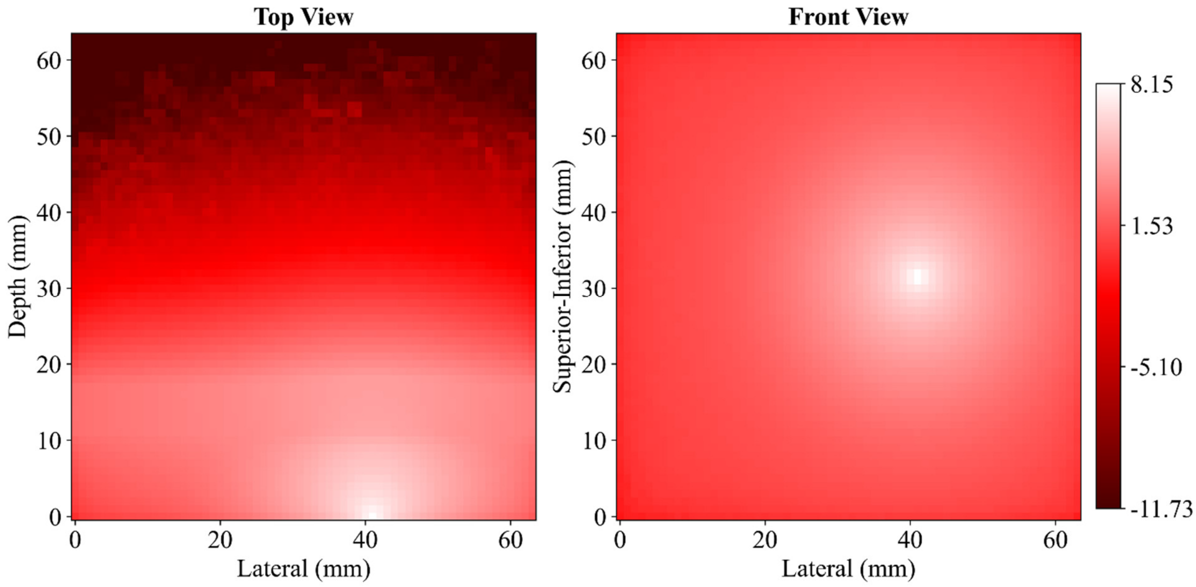


**Figure S56** Spatial fluence color maps for cadaveric head #6 at 19 mm source-detector separation from top view (left) and front view (right). The bright white spots indicate the position of the light source. The color bar represents photon fluence in a logarithmic scale to enhance visibility across a wide dynamic range.

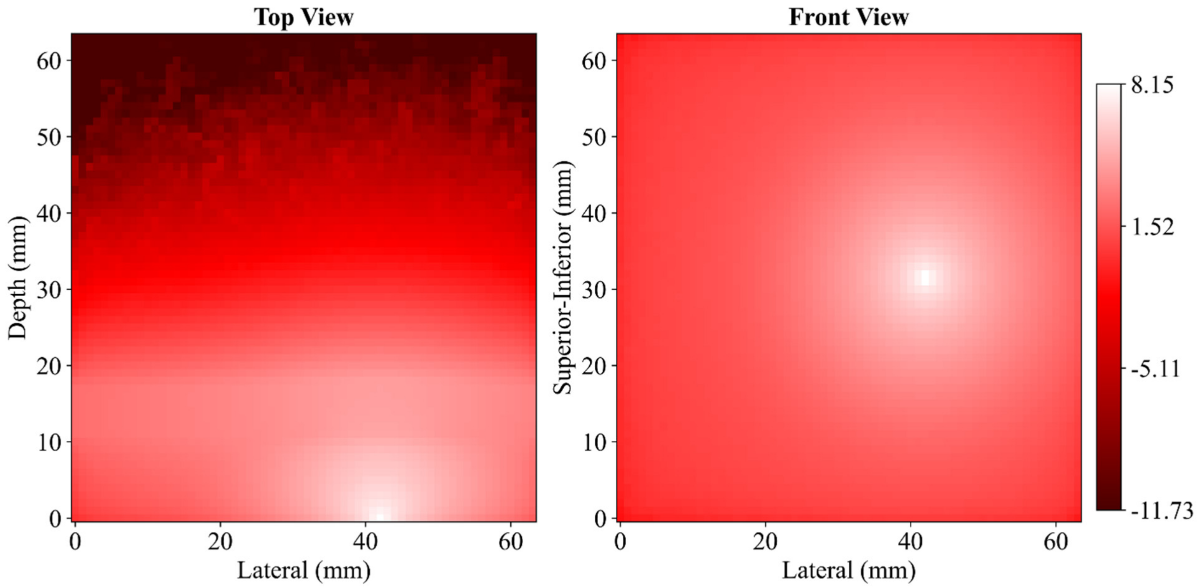


**Figure S57** Spatial fluence color maps for cadaveric head #6 at 21 mm source-detector separation from top view (left) and front view (right). The bright white spots indicate the position of the light source. The color bar represents photon fluence in a logarithmic scale to enhance visibility across a wide dynamic range.

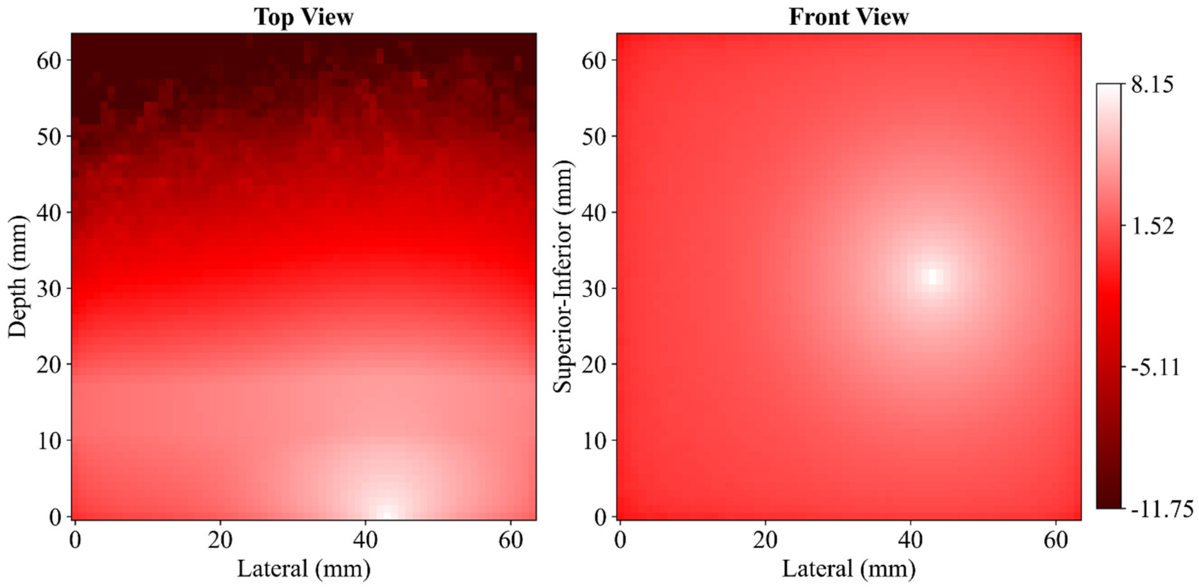


**Figure S58** Spatial fluence color maps for cadaveric head #6 at 23 mm source-detector separation from top view (left) and front view (right). The bright white spots indicate the position of the light source. The color bar represents photon fluence in a logarithmic scale to enhance visibility across a wide dynamic range.

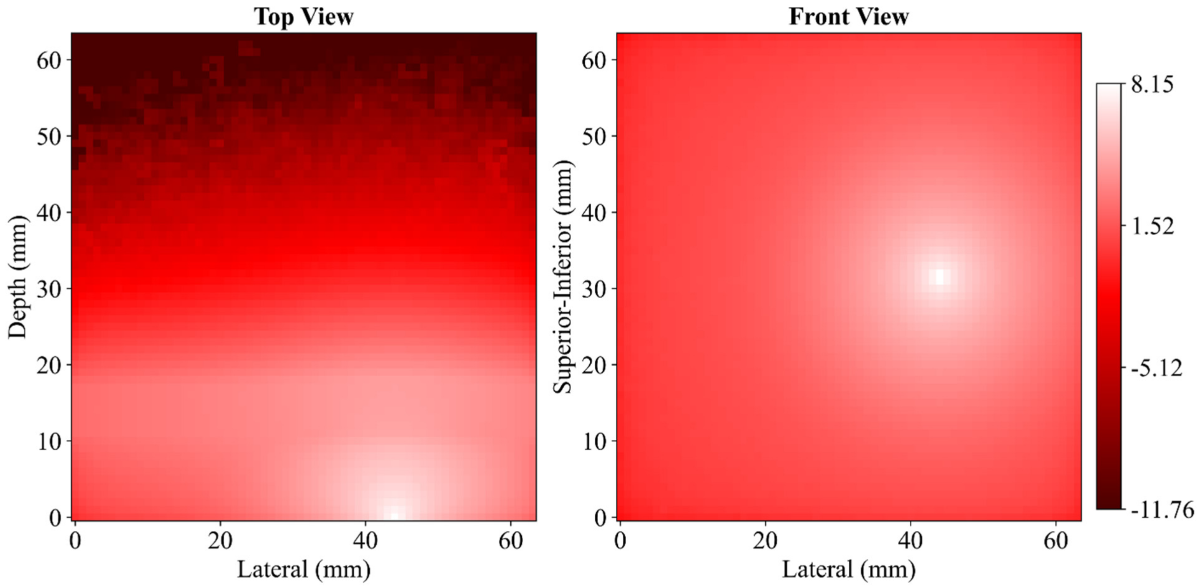


**Figure S59** Spatial fluence color maps for cadaveric head #6 at 25 mm source-detector separation from top view (left) and front view (right). The bright white spots indicate the position of the light source. The color bar represents photon fluence in a logarithmic scale to enhance visibility across a wide dynamic range.

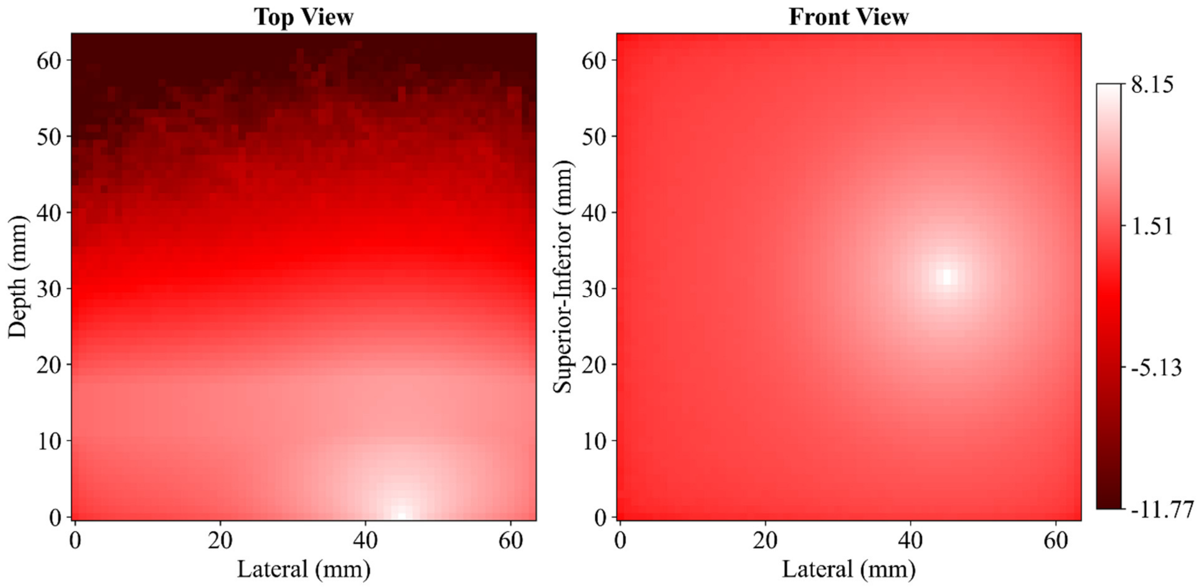


**Figure S60** Spatial fluence color maps for cadaveric head #6 at 27 mm source-detector separation from top view (left) and front view (right). The bright white spots indicate the position of the light source. The color bar represents photon fluence in a logarithmic scale to enhance visibility across a wide dynamic range.

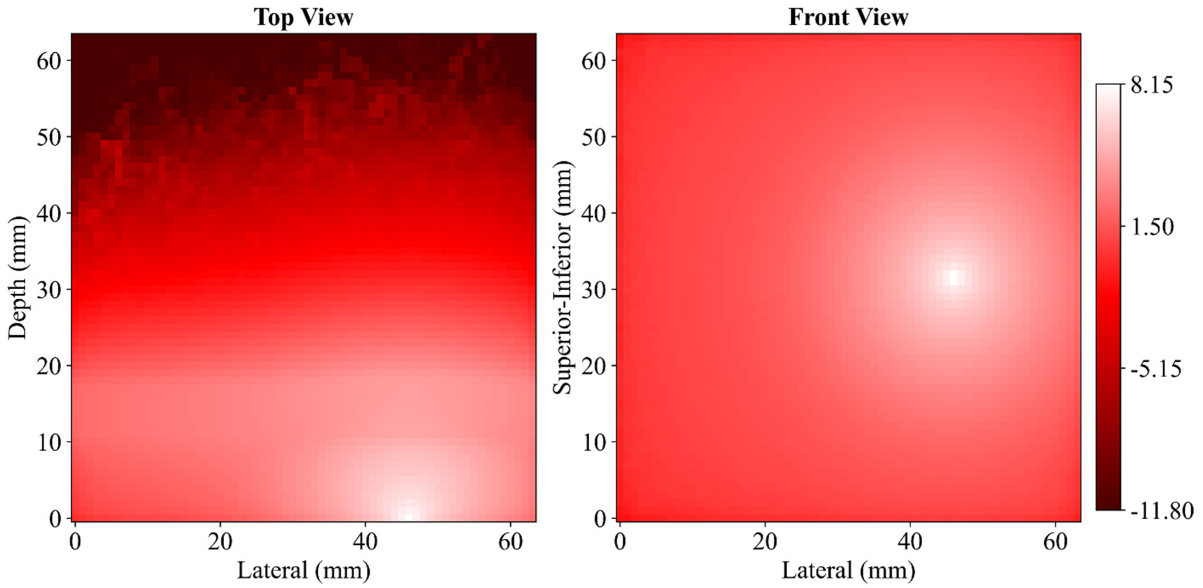


**Figure S61** Spatial fluence color maps for cadaveric head #6 at 29 mm source-detector separation from top view (left) and front view (right). The bright white spots indicate the position of the light source. The color bar represents photon fluence in a logarithmic scale to enhance visibility across a wide dynamic range.

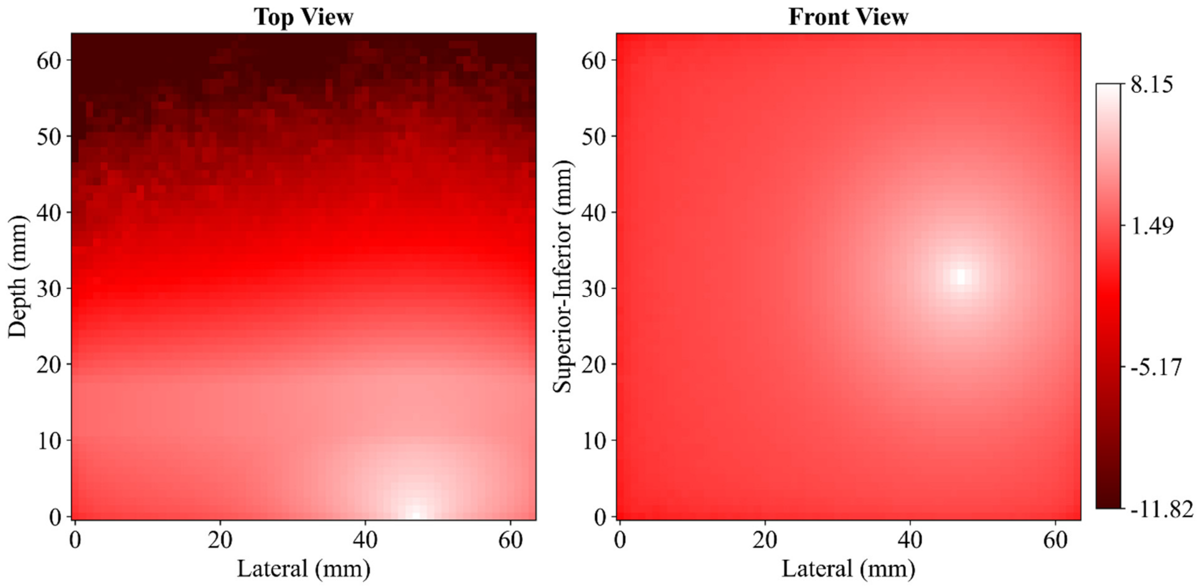


**Figure S62** Spatial fluence color maps for cadaveric head #6 at 31 mm source-detector separation from top view (left) and front view (right). The bright white spots indicate the position of the light source. The color bar represents photon fluence in a logarithmic scale to enhance visibility across a wide dynamic range.

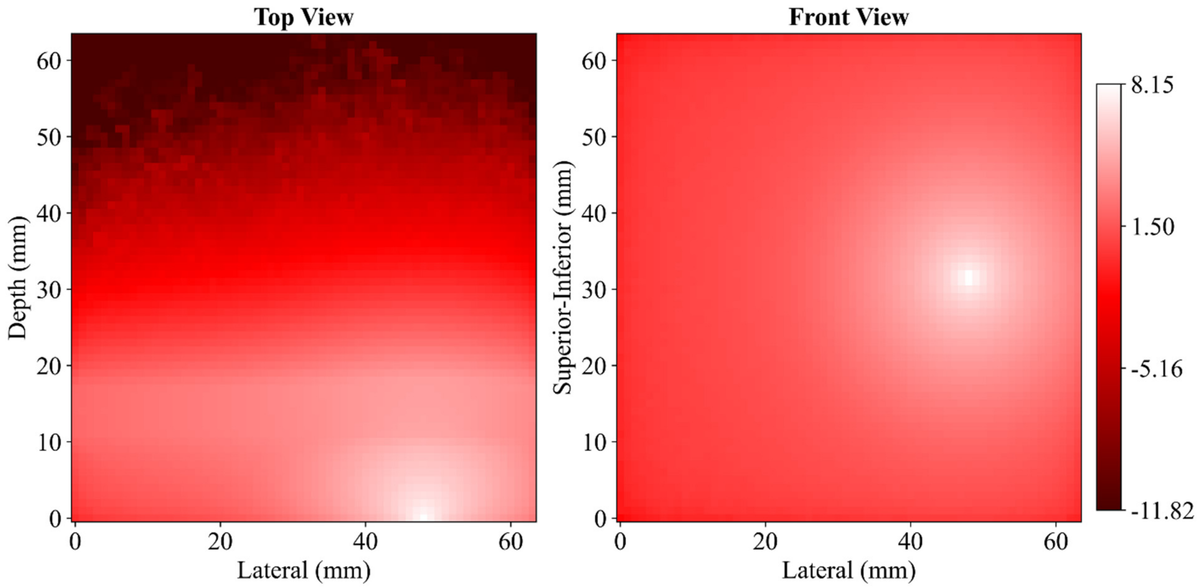


**Figure S63** Spatial fluence color maps for cadaveric head #6 at 33 mm source-detector separation from top view (left) and front view (right). The bright white spots indicate the position of the light source. The color bar represents photon fluence in a logarithmic scale to enhance visibility across a wide dynamic range.

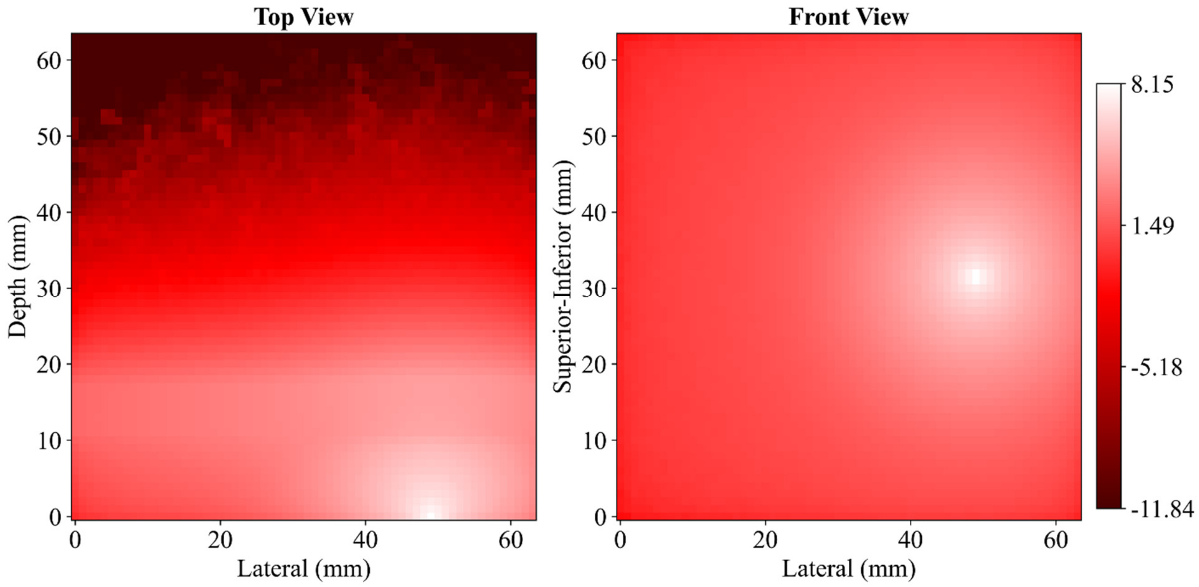


**Figure S64** Spatial fluence color maps for cadaveric head #6 at 35 mm source-detector separation from top view (left) and front view (right). The bright white spots indicate the position of the light source. The color bar represents photon fluence in a logarithmic scale to enhance visibility across a wide dynamic range.

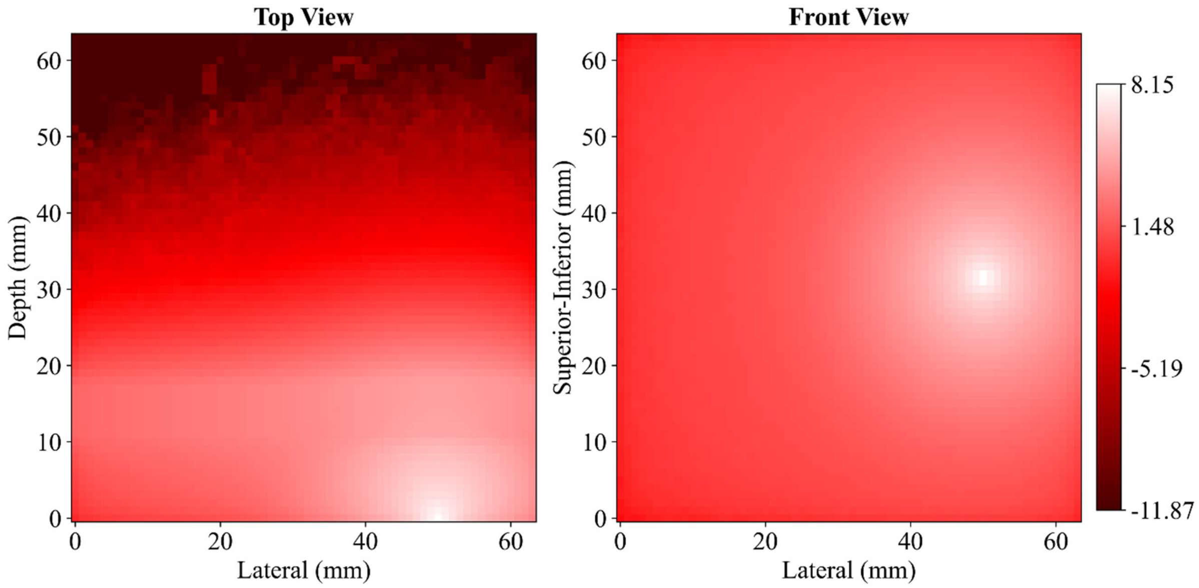


**Figure S65** Spatial fluence color maps for cadaveric head #6 at 37 mm source-detector separation from top view (left) and front view (right). The bright white spots indicate the position of the light source. The color bar represents photon fluence in a logarithmic scale to enhance visibility across a wide dynamic range.

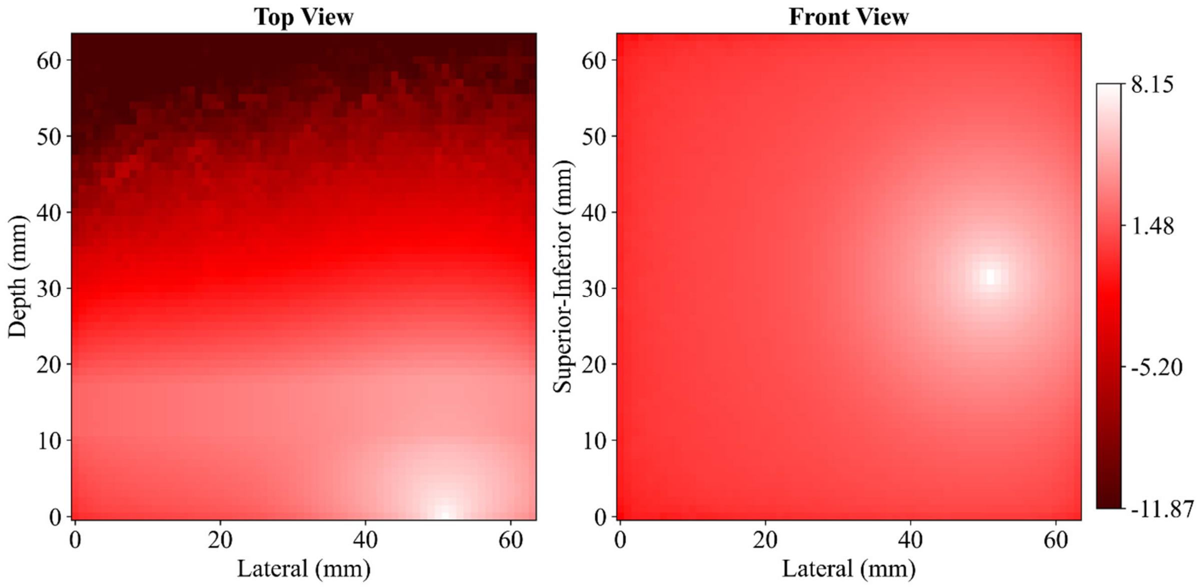


**Figure S66** Spatial fluence color maps for cadaveric head #6 at 39 mm source-detector separation from top view (left) and front view (right). The bright white spots indicate the position of the light source. The color bar represents photon fluence in a logarithmic scale to enhance visibility across a wide dynamic range.

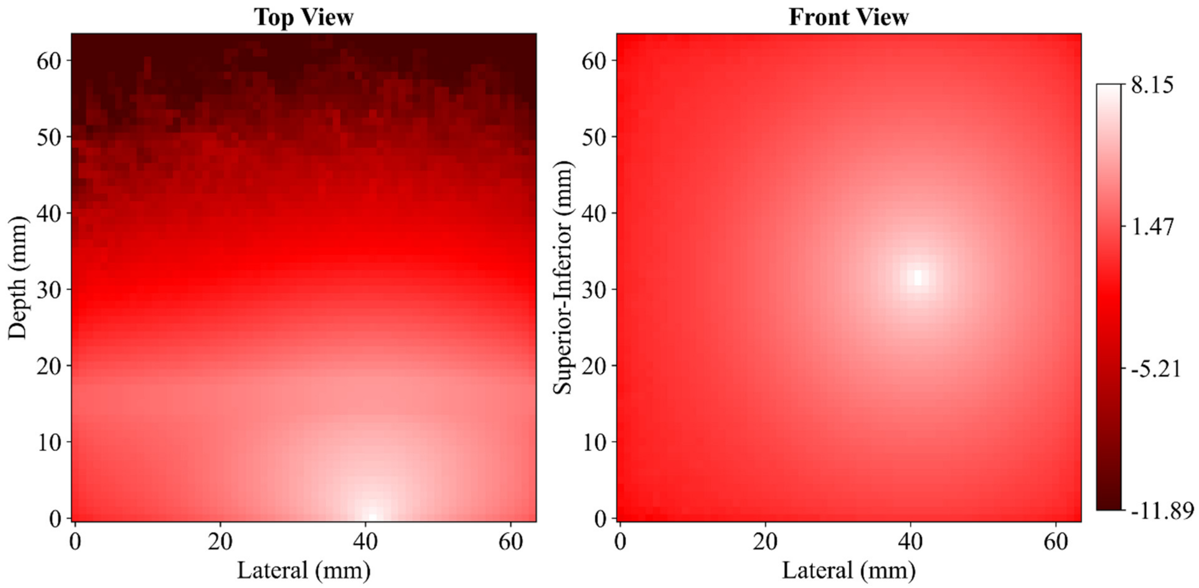


**Figure S67** Spatial fluence color maps for cadaveric head #7 at 19 mm source-detector separation from top view (left) and front view (right). The bright white spots indicate the position of the light source. The color bar represents photon fluence in a logarithmic scale to enhance visibility across a wide dynamic range.

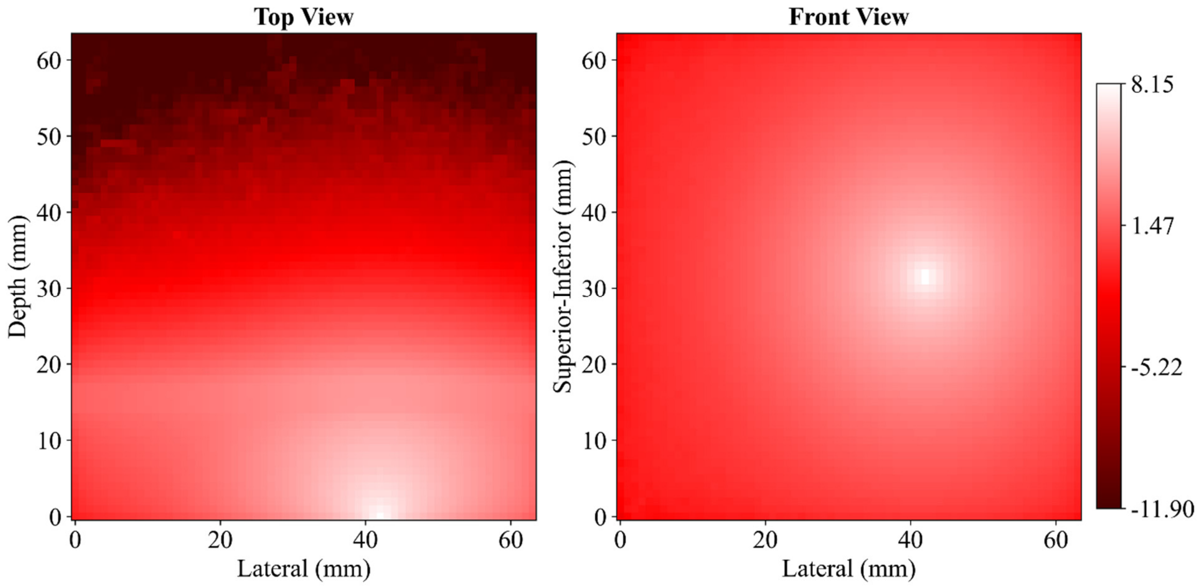


**Figure S68** Spatial fluence color maps for cadaveric head #7 at 21 mm source-detector separation from top view (left) and front view (right). The bright white spots indicate the position of the light source. The color bar represents photon fluence in a logarithmic scale to enhance visibility across a wide dynamic range.

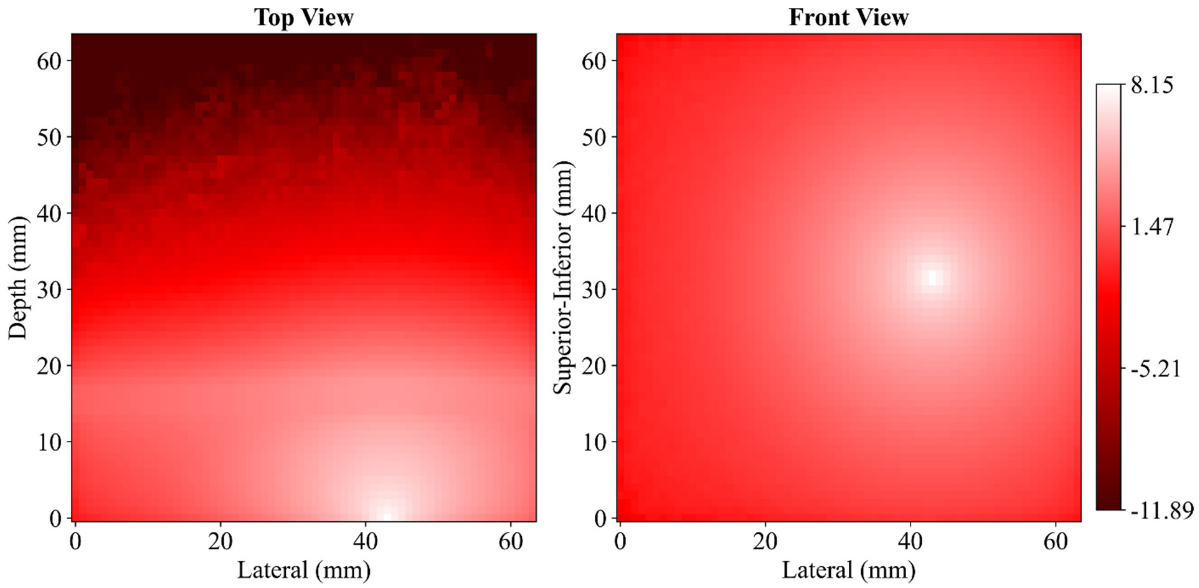


**Figure S69** Spatial fluence color maps for cadaveric head #7 at 23 mm source-detector separation from top view (left) and front view (right). The bright white spots indicate the position of the light source. The color bar represents photon fluence in a logarithmic scale to enhance visibility across a wide dynamic range.

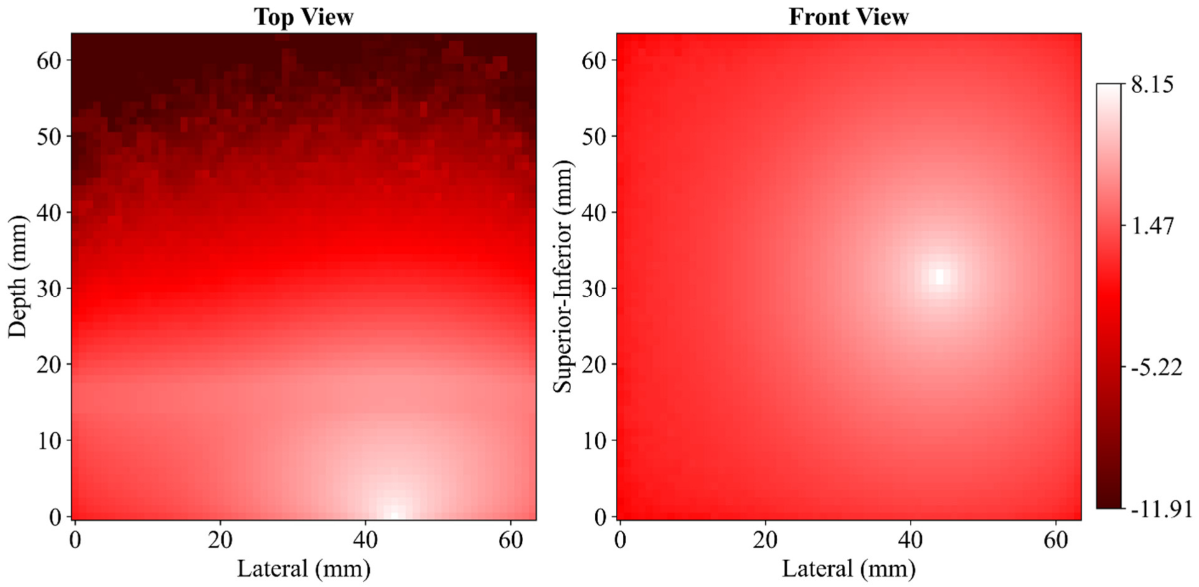


**Figure S70** Spatial fluence color maps for cadaveric head #7 at 25 mm source-detector separation from top view (left) and front view (right). The bright white spots indicate the position of the light source. The color bar represents photon fluence in a logarithmic scale to enhance visibility across a wide dynamic range.

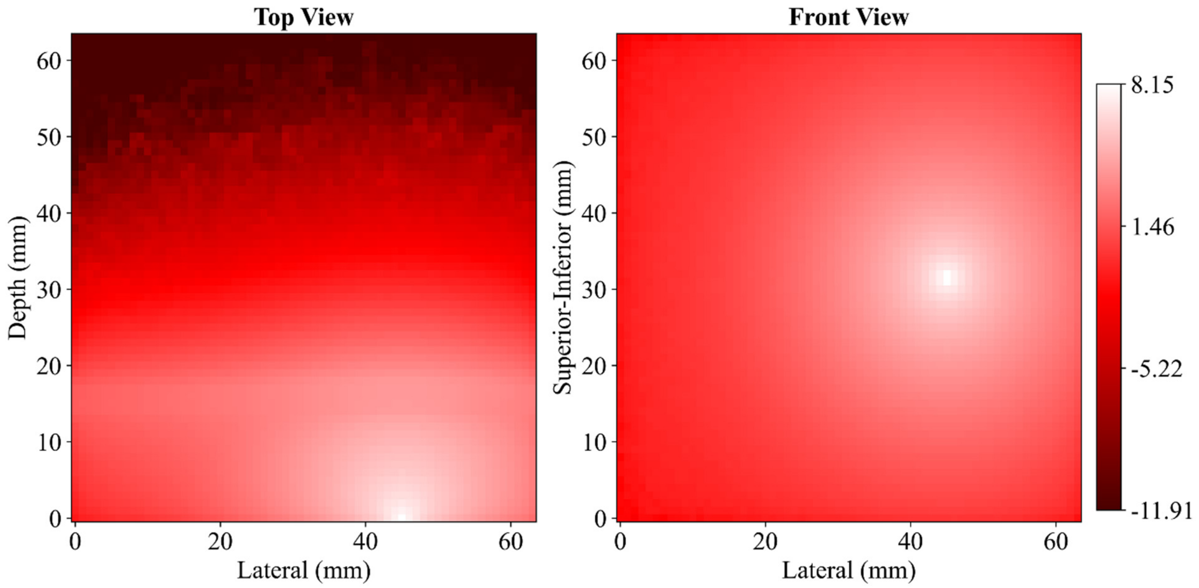


**Figure S71** Spatial fluence color maps for cadaveric head #7 at 27 mm source-detector separation from top view (left) and front view (right). The bright white spots indicate the position of the light source. The color bar represents photon fluence in a logarithmic scale to enhance visibility across a wide dynamic range.

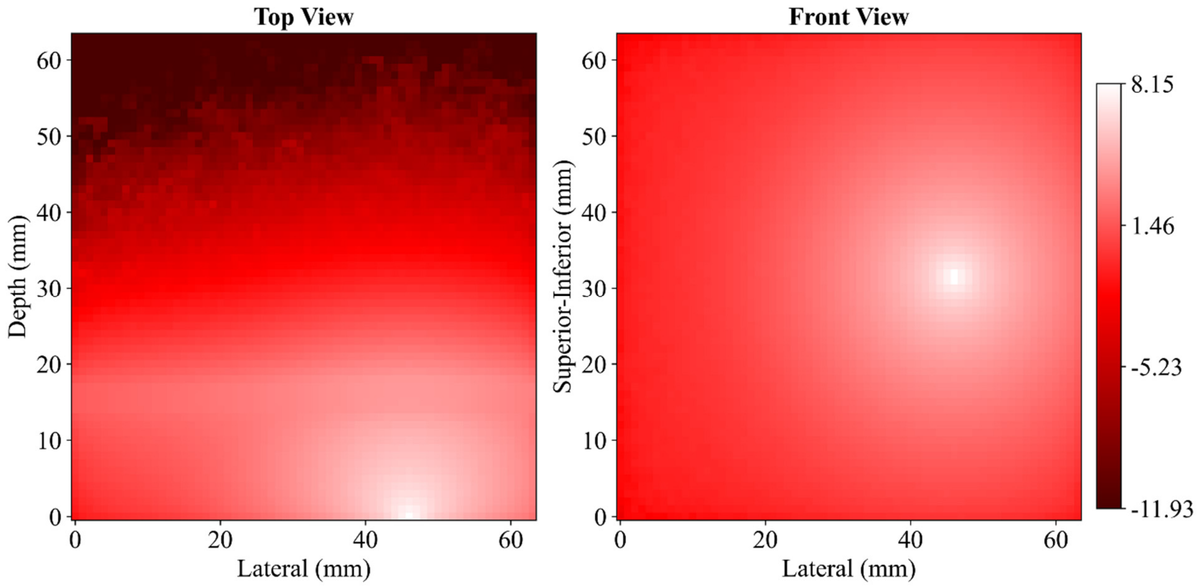


**Figure S72** Spatial fluence color maps for cadaveric head #7 at 29 mm source-detector separation from top view (left) and front view (right). The bright white spots indicate the position of the light source. The color bar represents photon fluence in a logarithmic scale to enhance visibility across a wide dynamic range.

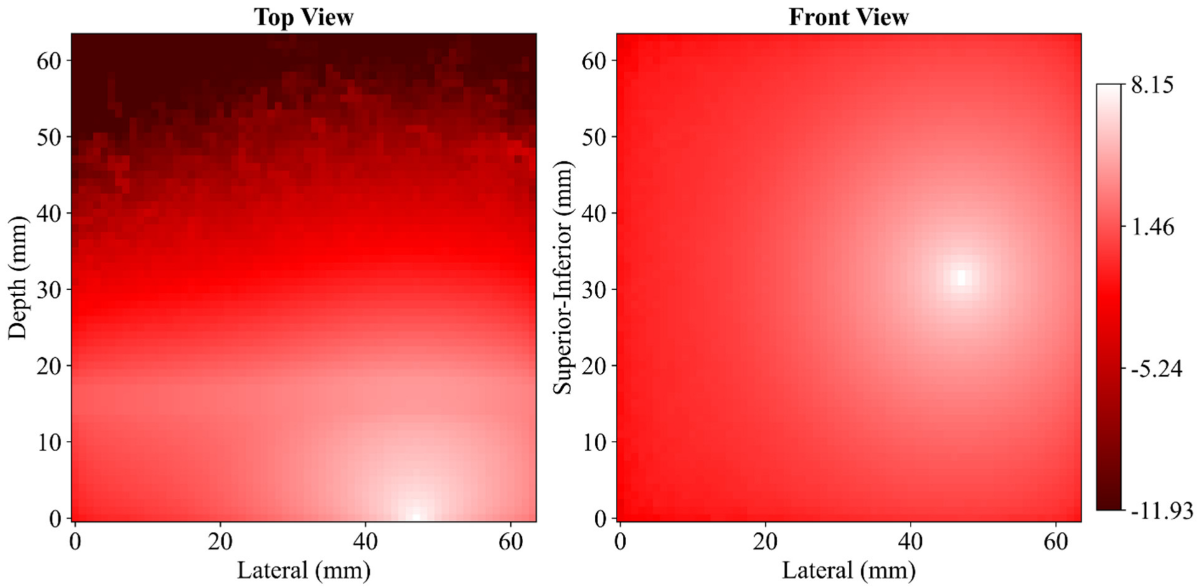


**Figure S73** Spatial fluence color maps for cadaveric head #7 at 31 mm source-detector separation from top view (left) and front view (right). The bright white spots indicate the position of the light source. The color bar represents photon fluence in a logarithmic scale to enhance visibility across a wide dynamic range.

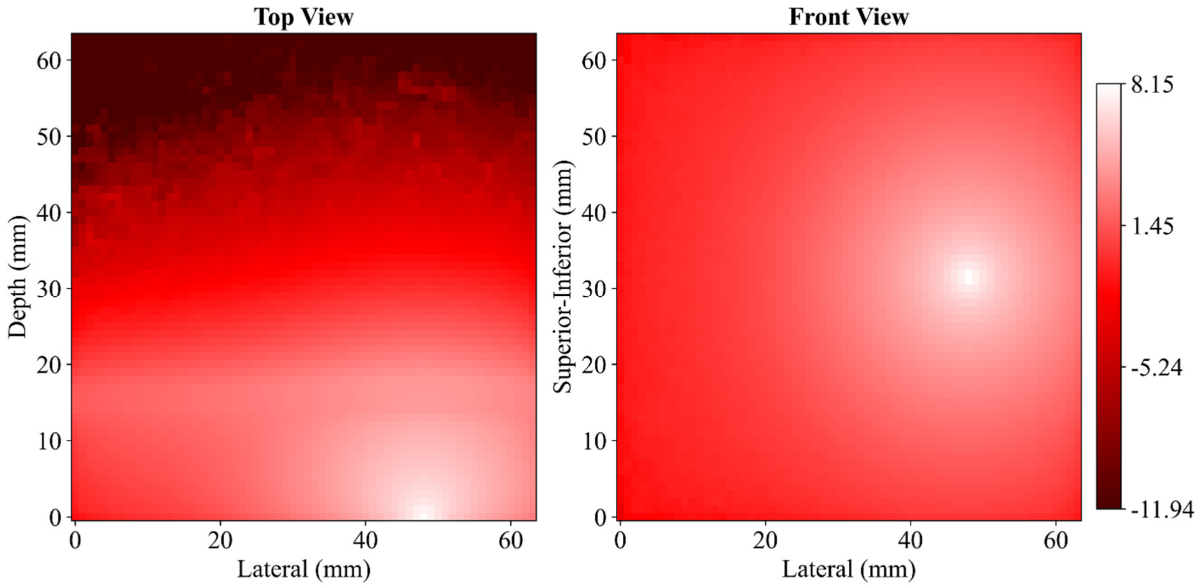


**Figure S74** Spatial fluence color maps for cadaveric head #7 at 33 mm source-detector separation from top view (left) and front view (right). The bright white spots indicate the position of the light source. The color bar represents photon fluence in a logarithmic scale to enhance visibility across a wide dynamic range.

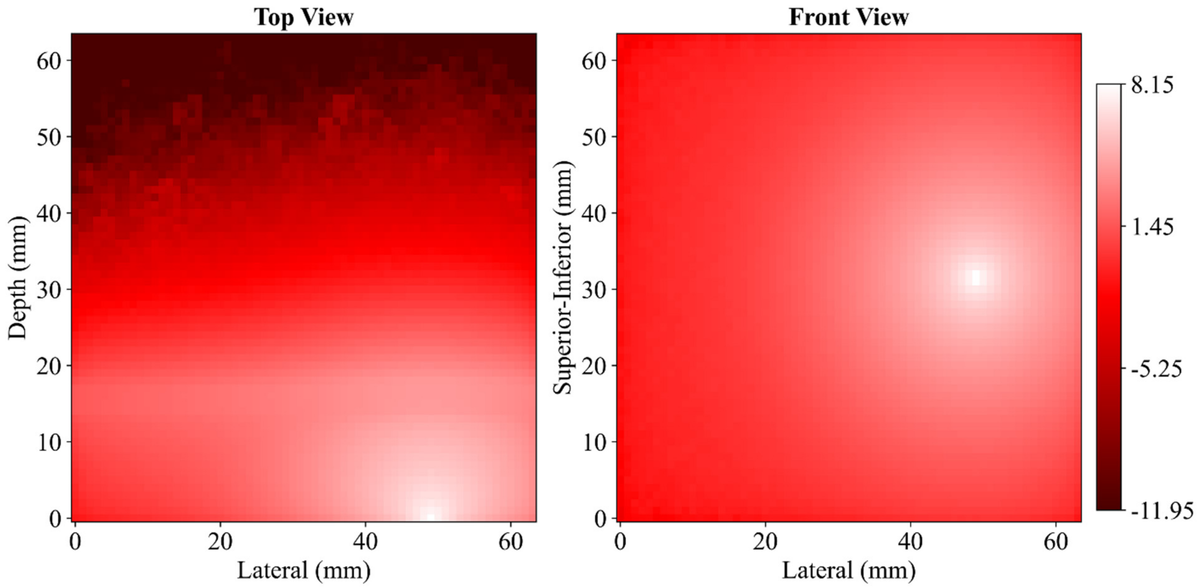


**Figure S75** Spatial fluence color maps for cadaveric head #7 at 35 mm source-detector separation from top view (left) and front view (right). The bright white spots indicate the position of the light source. The color bar represents photon fluence in a logarithmic scale to enhance visibility across a wide dynamic range.

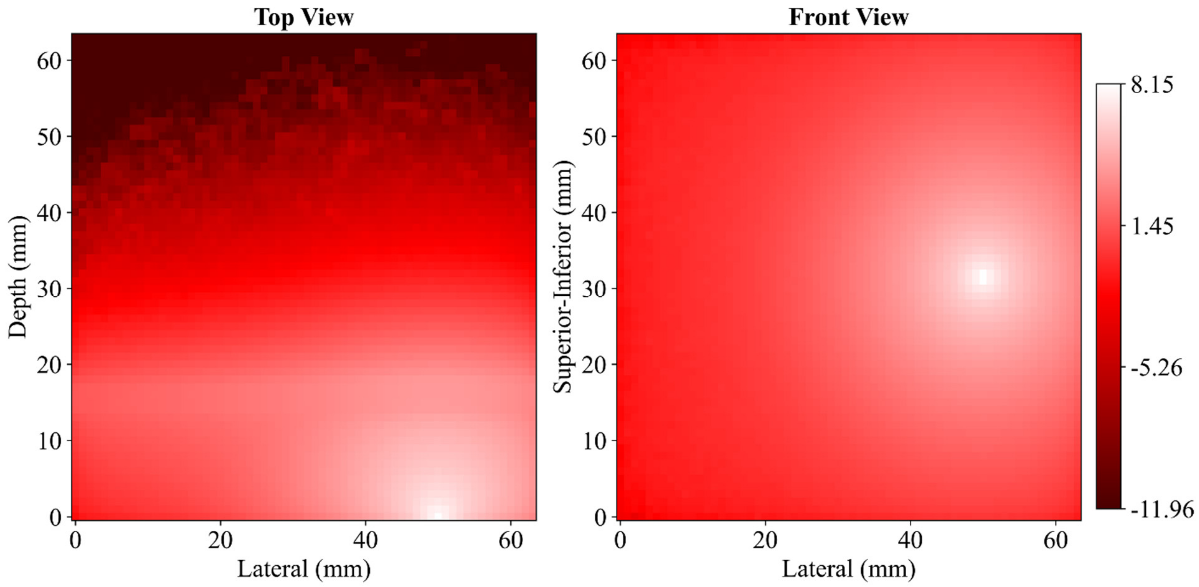


**Figure S76** Spatial fluence color maps for cadaveric head #7 at 37 mm source-detector separation from top view (left) and front view (right). The bright white spots indicate the position of the light source. The color bar represents photon fluence in a logarithmic scale to enhance visibility across a wide dynamic range.

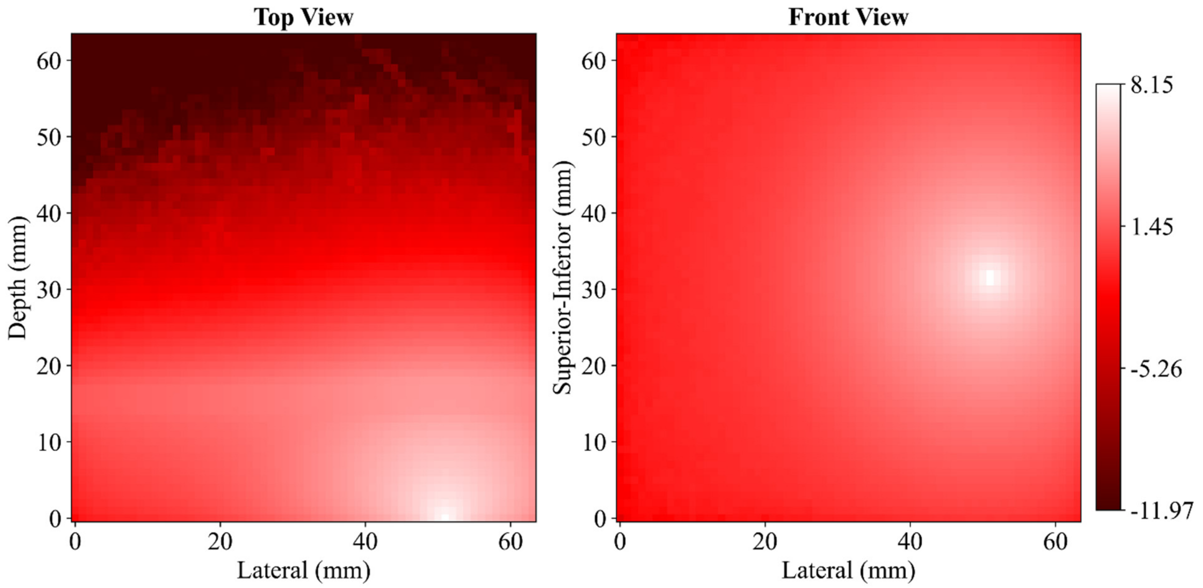


**Figure S77** Spatial fluence color maps for cadaveric head #7 at 39 mm source-detector separation from top view (left) and front view (right). The bright white spots indicate the position of the light source. The color bar represents photon fluence in a logarithmic scale to enhance visibility across a wide dynamic range.

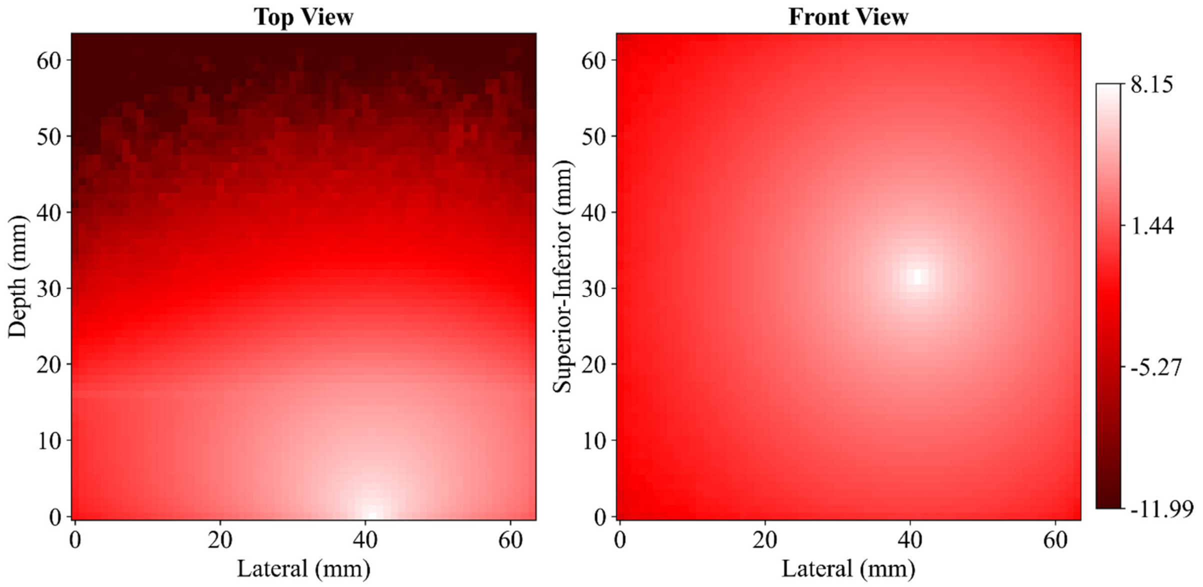


**Figure S78** Spatial fluence color maps for cadaveric head #8 at 19 mm source-detector separation from top view (left) and front view (right). The bright white spots indicate the position of the light source. The color bar represents photon fluence in a logarithmic scale to enhance visibility across a wide dynamic range.

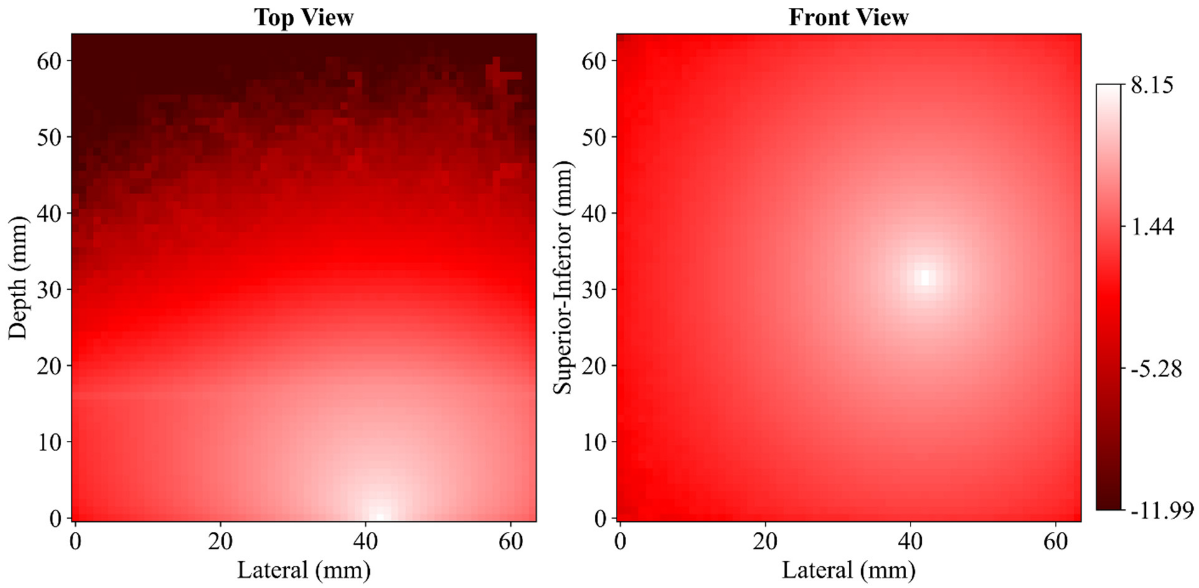


**Figure S79** Spatial fluence color maps for cadaveric head #8 at 21 mm source-detector separation from top view (left) and front view (right). The bright white spots indicate the position of the light source. The color bar represents photon fluence in a logarithmic scale to enhance visibility across a wide dynamic range.

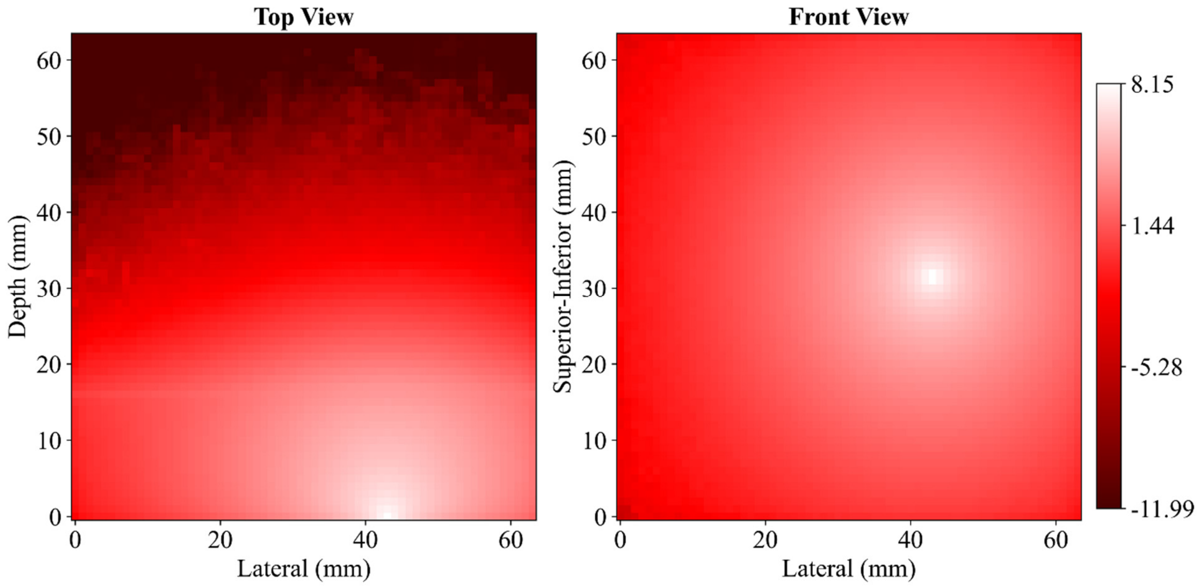


**Figure S80** Spatial fluence color maps for cadaveric head #8 at 23 mm source-detector separation from top view (left) and front view (right). The bright white spots indicate the position of the light source. The color bar represents photon fluence in a logarithmic scale to enhance visibility across a wide dynamic range.

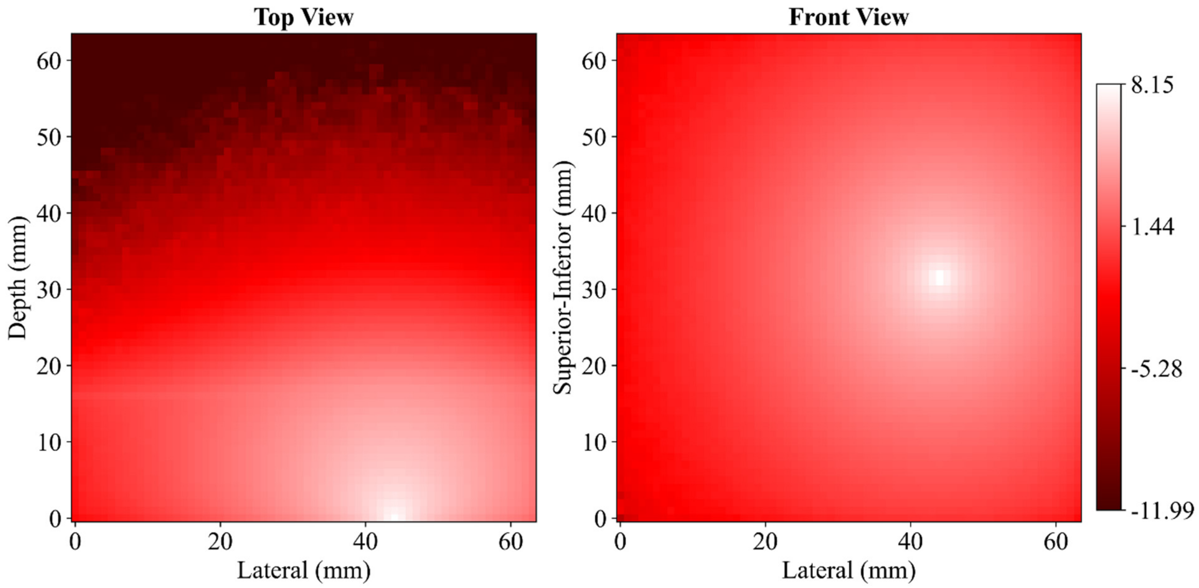


**Figure S81** Spatial fluence color maps for cadaveric head #8 at 25 mm source-detector separation from top view (left) and front view (right). The bright white spots indicate the position of the light source. The color bar represents photon fluence in a logarithmic scale to enhance visibility across a wide dynamic range.

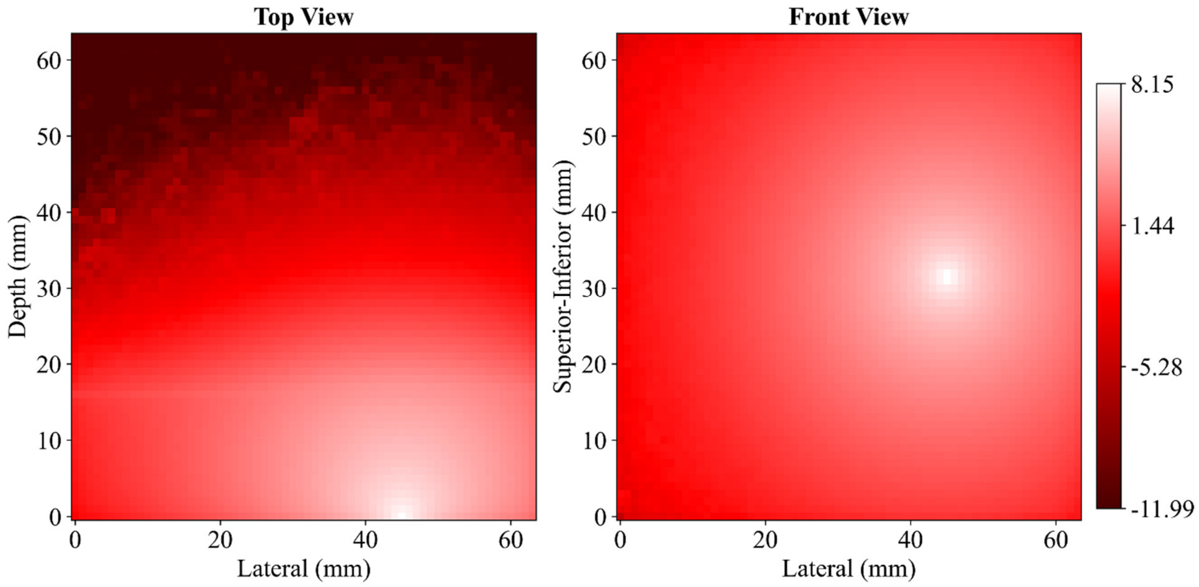


**Figure S82** Spatial fluence color maps for cadaveric head #8 at 27 mm source-detector separation from top view (left) and front view (right). The bright white spots indicate the position of the light source. The color bar represents photon fluence in a logarithmic scale to enhance visibility across a wide dynamic range.

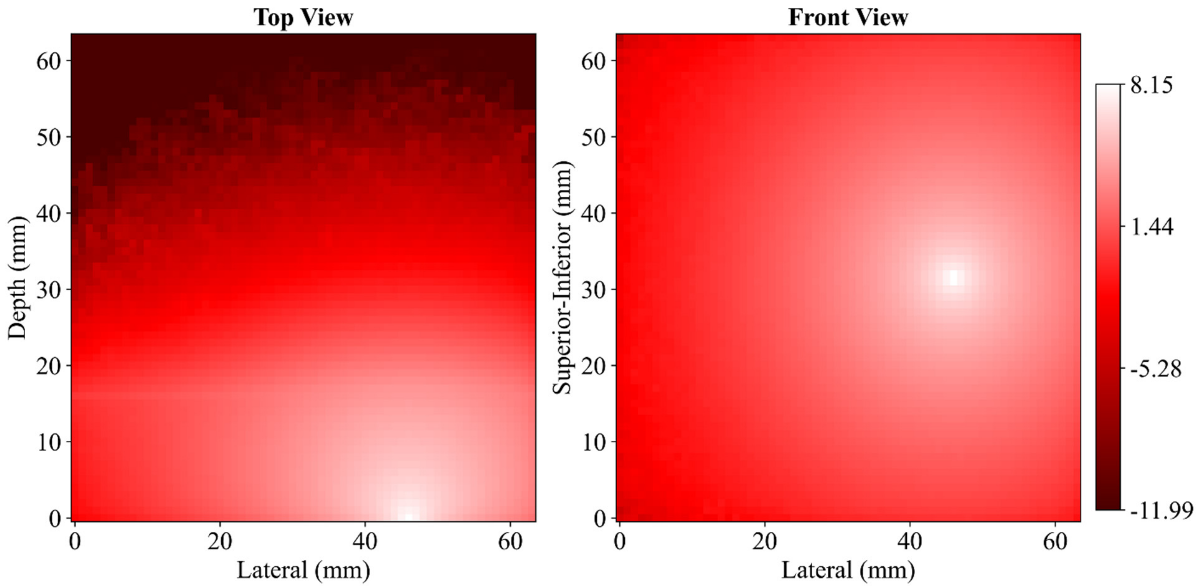


**Figure S83** Spatial fluence color maps for cadaveric head #8 at 29 mm source-detector separation from top view (left) and front view (right). The bright white spots indicate the position of the light source. The color bar represents photon fluence in a logarithmic scale to enhance visibility across a wide dynamic range.

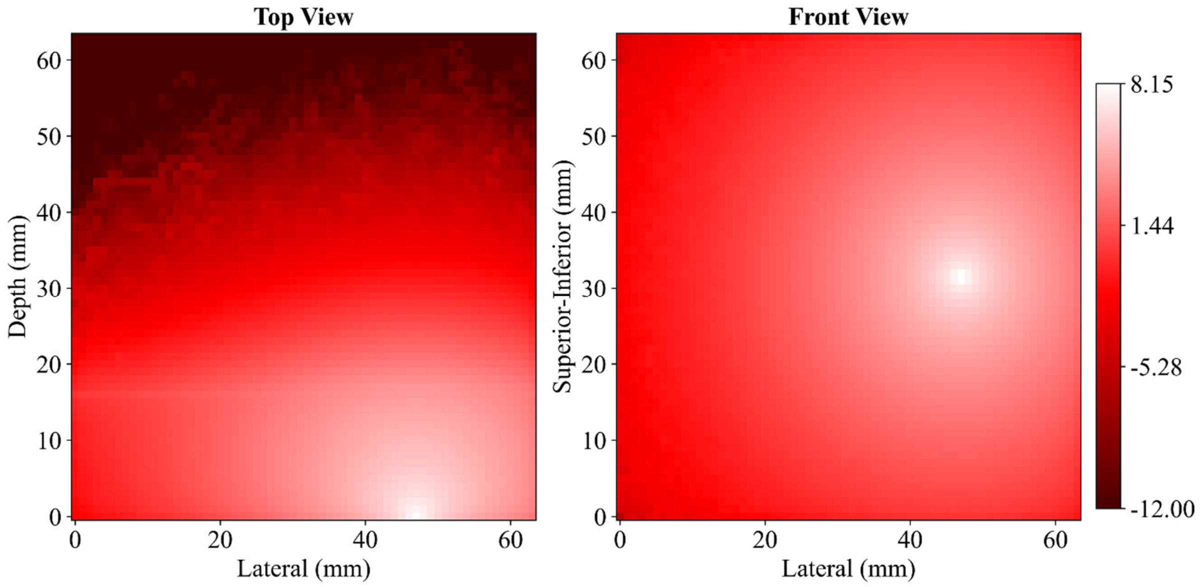


**Figure S84** Spatial fluence color maps for cadaveric head #8 at 31 mm source-detector separation from top view (left) and front view (right). The bright white spots indicate the position of the light source. The color bar represents photon fluence in a logarithmic scale to enhance visibility across a wide dynamic range.

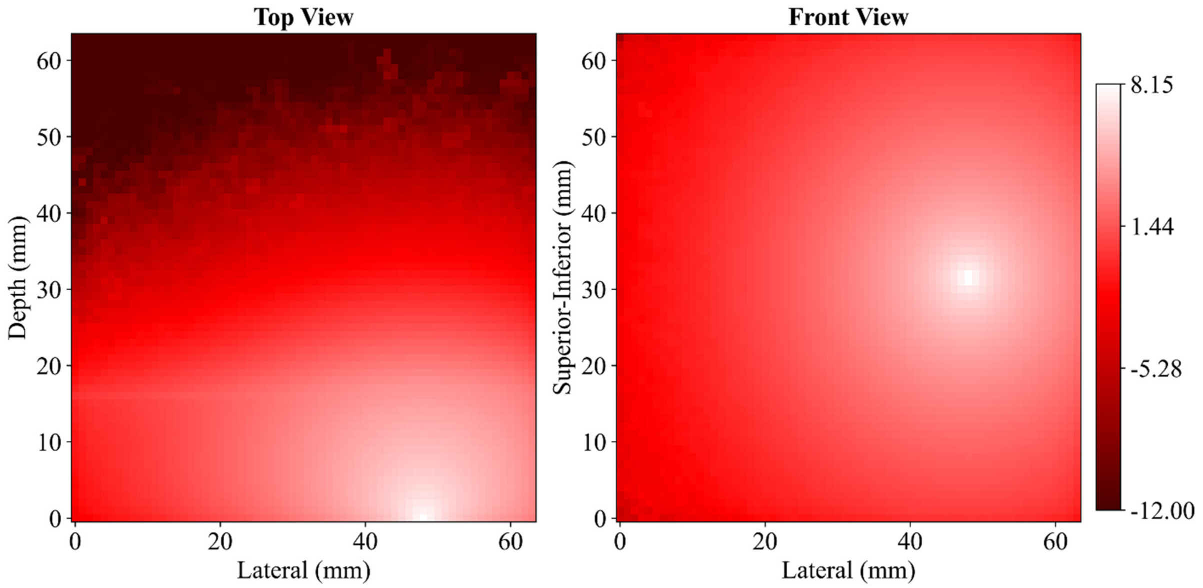


**Figure S85** Spatial fluence color maps for cadaveric head #8 at 33 mm source-detector separation from top view (left) and front view (right). The bright white spots indicate the position of the light source. The color bar represents photon fluence in a logarithmic scale to enhance visibility across a wide dynamic range.

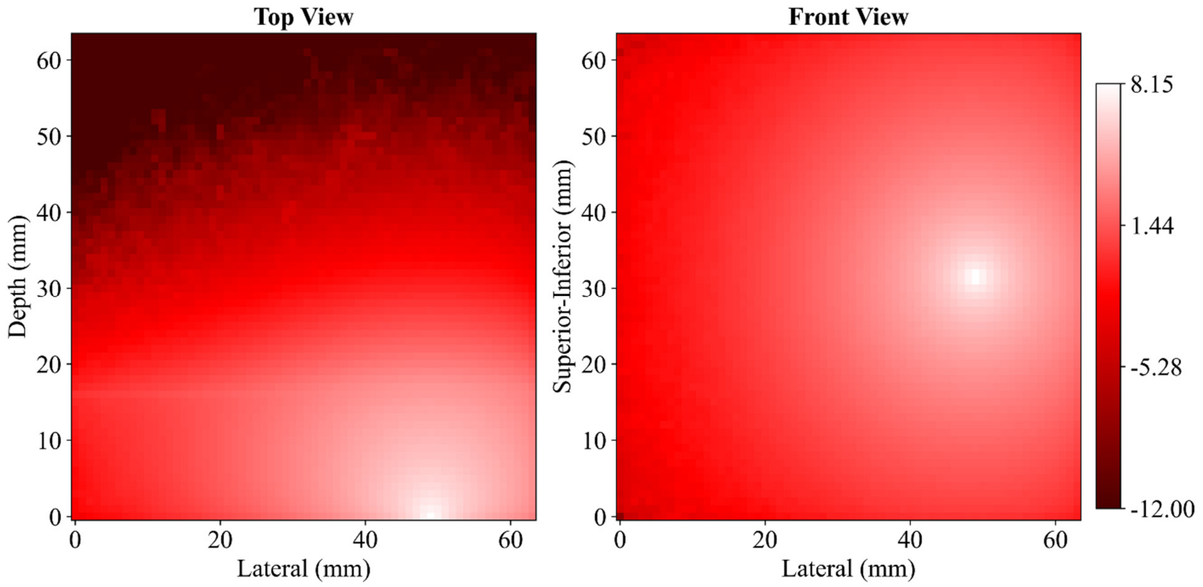


**Figure S86** Spatial fluence color maps for cadaveric head #8 at 35 mm source-detector separation from top view (left) and front view (right). The bright white spots indicate the position of the light source. The color bar represents photon fluence in a logarithmic scale to enhance visibility across a wide dynamic range.

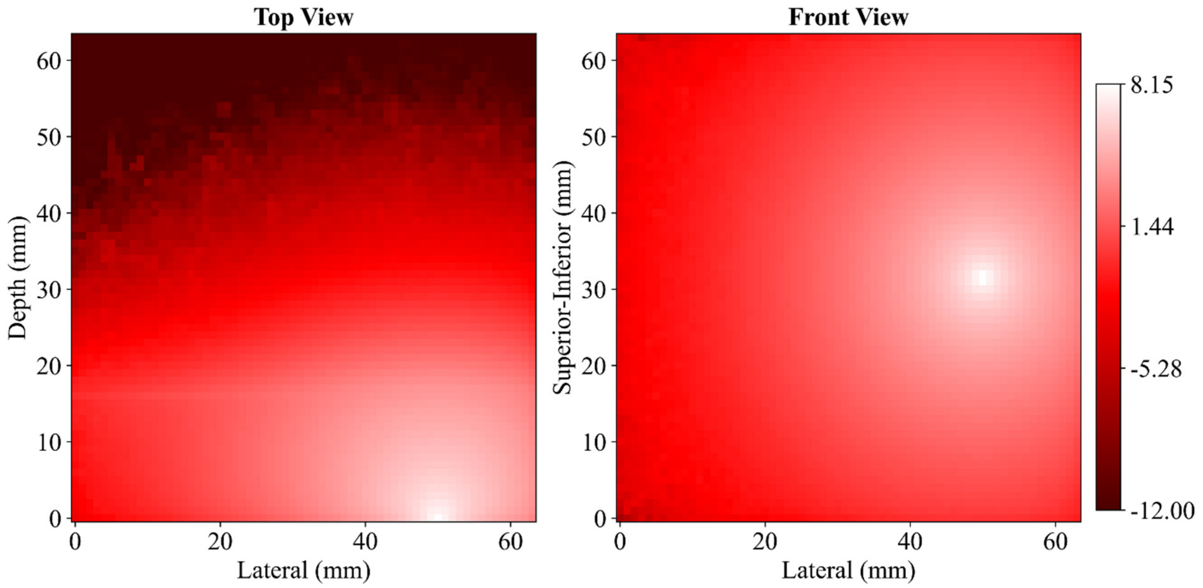


**Figure S87** Spatial fluence color maps for cadaveric head #8 at 37 mm source-detector separation from top view (left) and front view (right). The bright white spots indicate the position of the light source. The color bar represents photon fluence in a logarithmic scale to enhance visibility across a wide dynamic range.

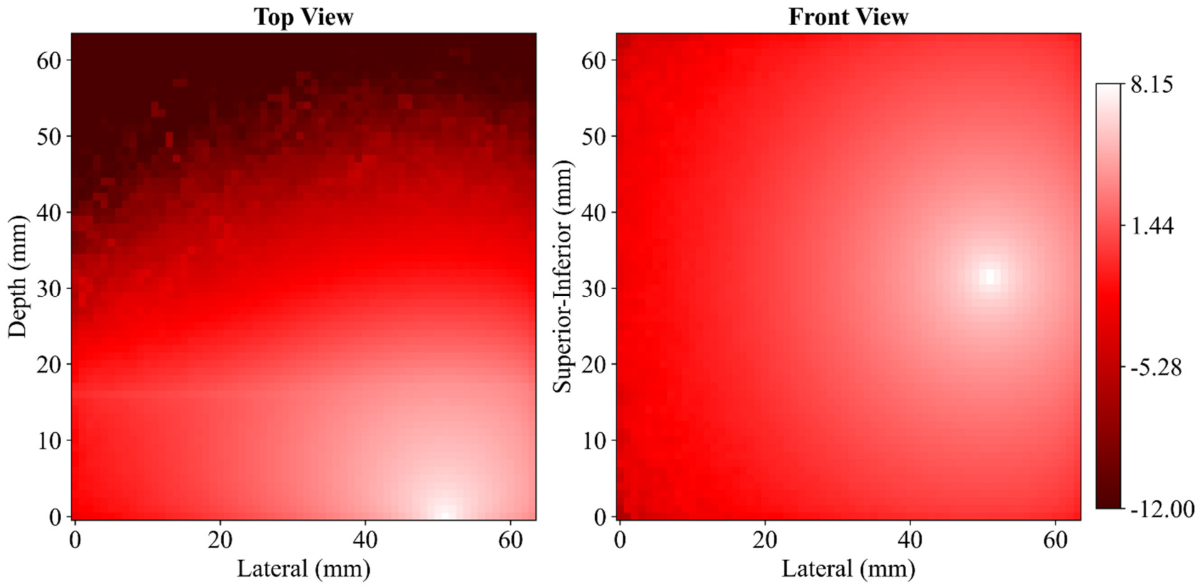


**Figure S88** Spatial fluence color maps for cadaveric head #8 at 39 mm source-detector separation from top view (left) and front view (right). The bright white spots indicate the position of the light source. The color bar represents photon fluence in a logarithmic scale to enhance visibility across a wide dynamic range.

## B. Sensitivity at Depth vs. Depth (2D Plots)

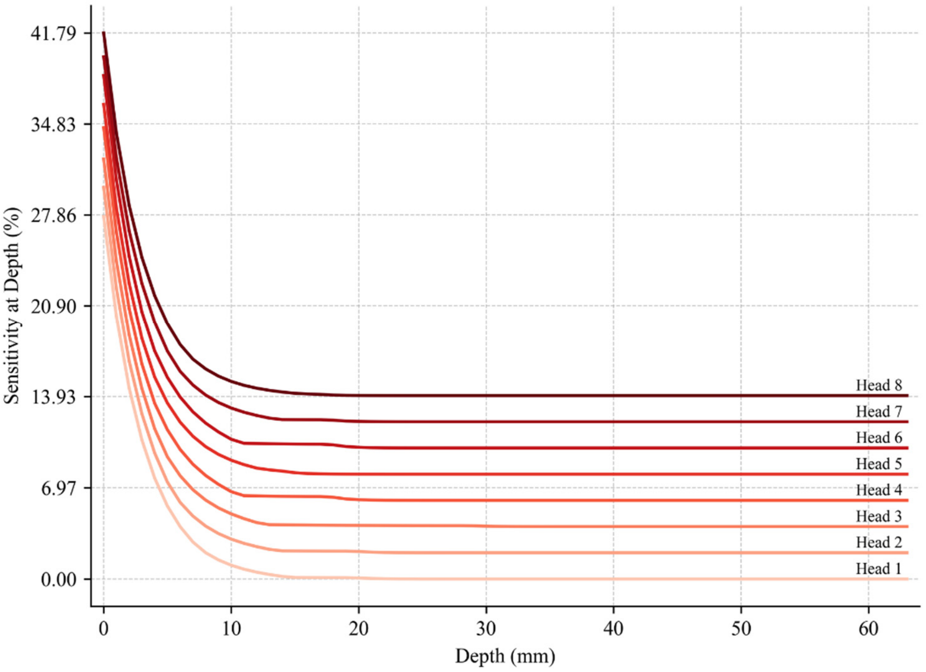


**Figure S89** Sensitivity at depth vs. depth 2D plots for all cadaveric heads at 19 mm source-detector separation. Curves correspond to Heads #1–8, shown from lightest to darkest red. For clarity, curves for Heads #2–8 are vertically shifted in 2% increments to reduce overlap.

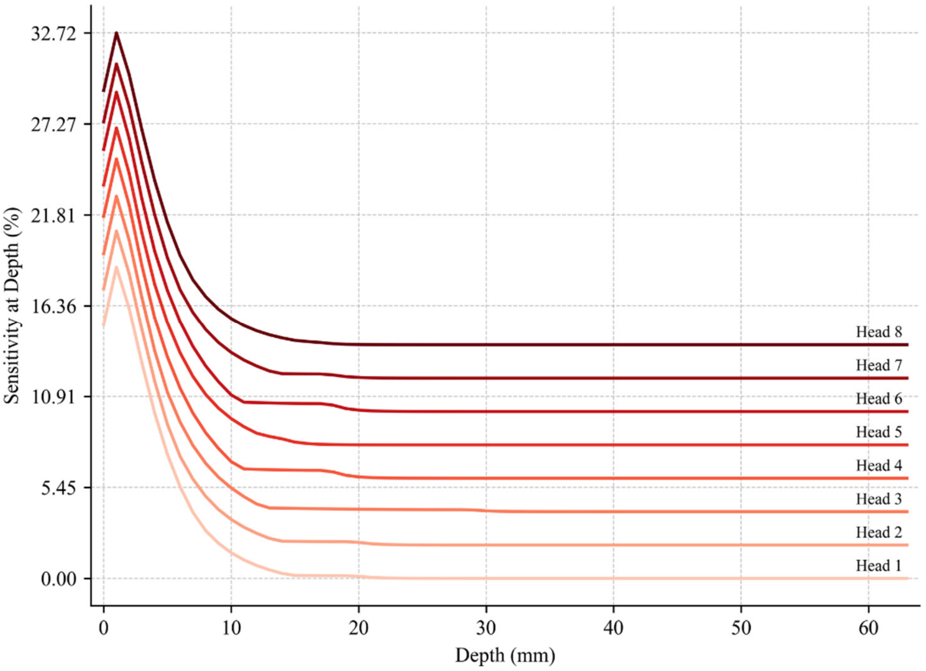


**Figure S90** Sensitivity at depth vs. depth 2D plots for all cadaveric heads at 21 mm source-detector separation. Curves correspond to Heads #1–8, shown from lightest to darkest red. For clarity, curves for Heads #2–8 are vertically shifted in 2% increments to reduce overlap.

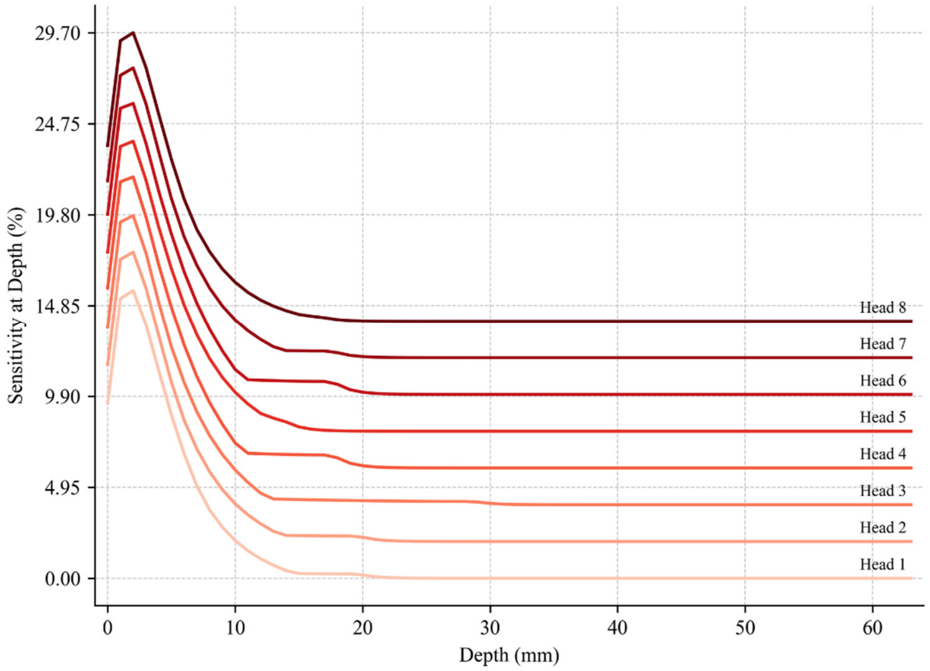


**Figure S91** Sensitivity at depth vs. depth 2D plots for all cadaveric heads at 23 mm source-detector separation. Curves correspond to Heads #1–8, shown from lightest to darkest red. For clarity, curves for Heads #2–8 are vertically shifted in 2% increments to reduce overlap.

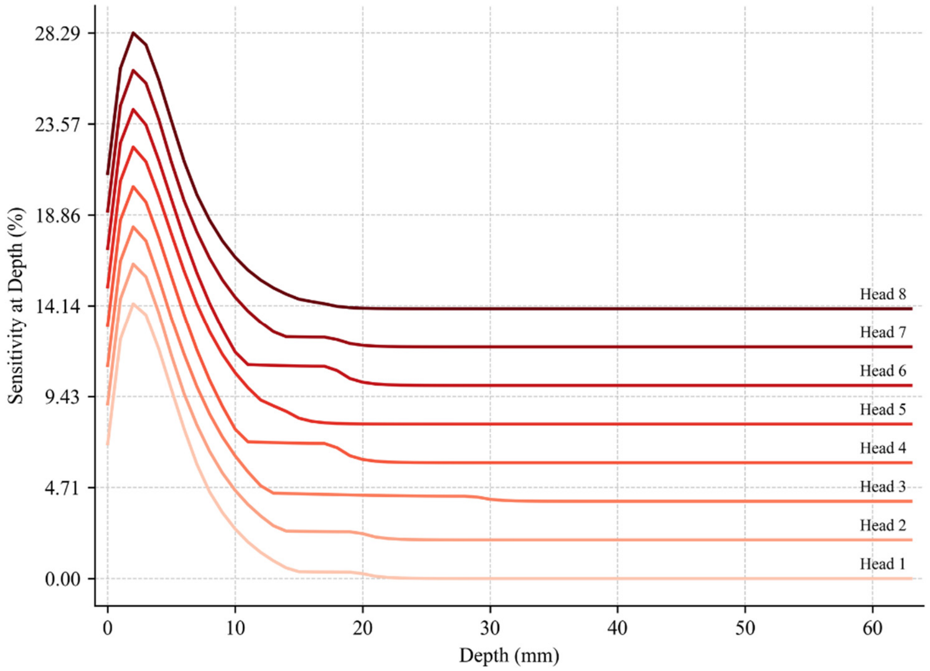


**Figure S92** Sensitivity at depth vs. depth 2D plots for all cadaveric heads at 25 mm source-detector separation. Curves correspond to Heads #1–8, shown from lightest to darkest red. For clarity, curves for Heads #2–8 are vertically shifted in 2% increments to reduce overlap.

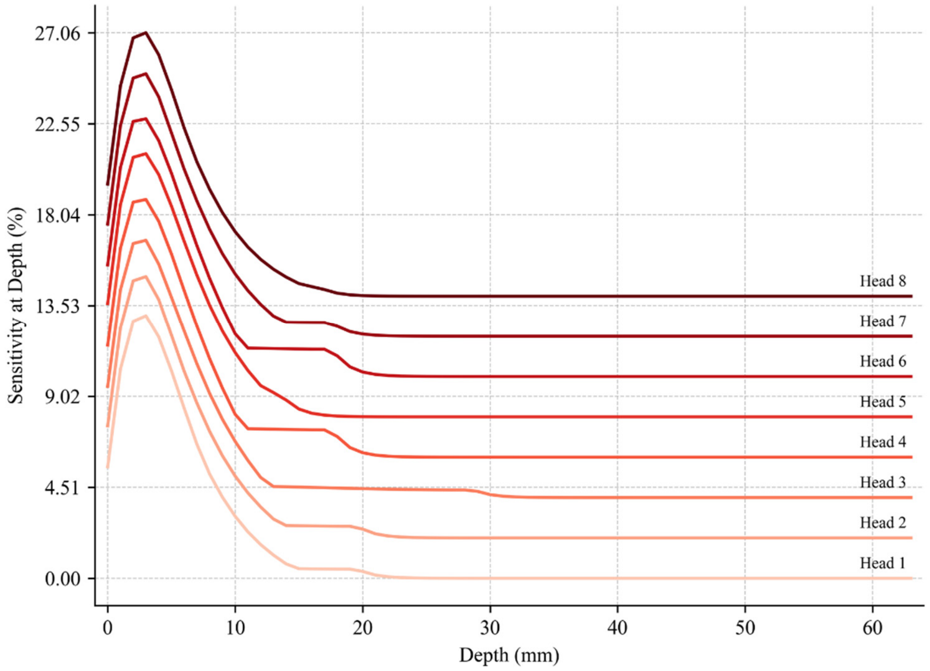


**Figure S93** Sensitivity at depth vs. depth 2D plots for all cadaveric heads at 27 mm source-detector separation. Curves correspond to Heads #1–8, shown from lightest to darkest red. For clarity, curves for Heads #2–8 are vertically shifted in 2% increments to reduce overlap.

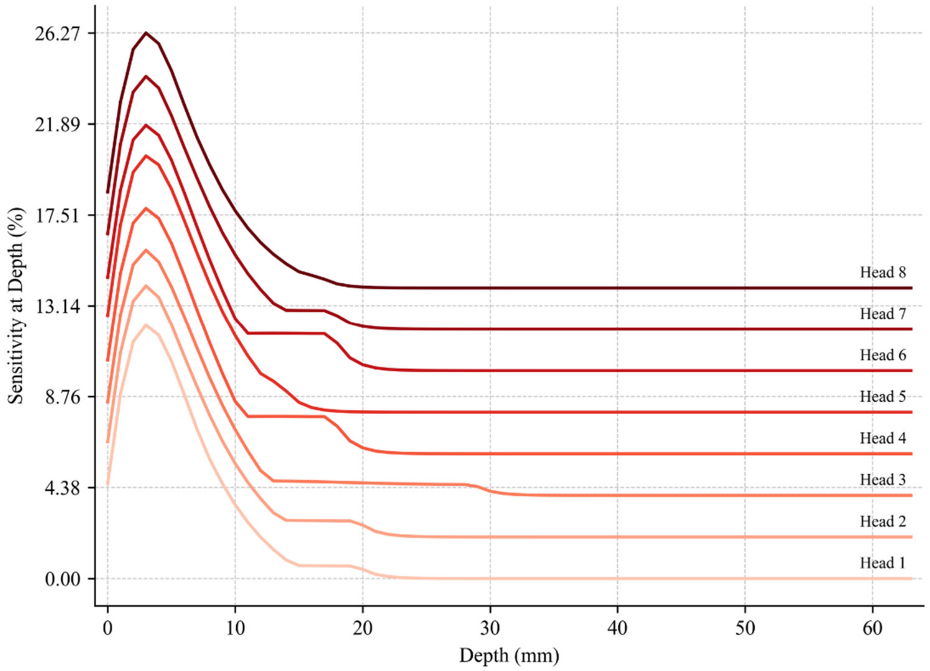


**Figure S94** Sensitivity at depth vs. depth 2D plots for all cadaveric heads at 29 mm source-detector separation. Curves correspond to Heads #1–8, shown from lightest to darkest red. For clarity, curves for Heads #2–8 are vertically shifted in 2% increments to reduce overlap.

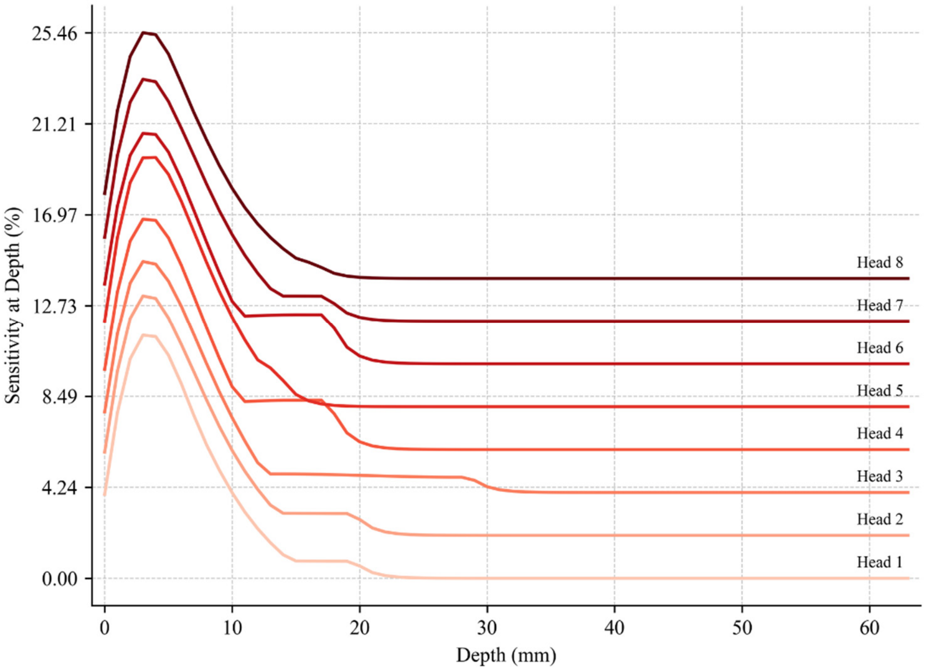


**Figure S95** Sensitivity at depth vs. depth 2D plots for all cadaveric heads at 31 mm source-detector separation. Curves correspond to Heads #1–8, shown from lightest to darkest red. For clarity, curves for Heads #2–8 are vertically shifted in 2% increments to reduce overlap.

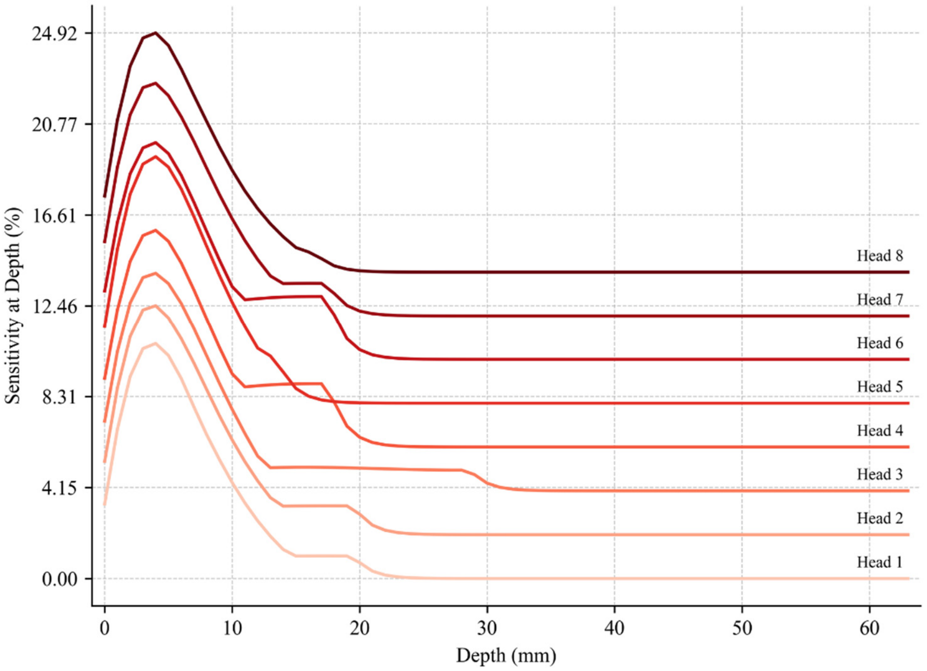


**Figure S96** Sensitivity at depth vs. depth 2D plots for all cadaveric heads at 33 mm source-detector separation. Curves correspond to Heads #1–8, shown from lightest to darkest red. For clarity, curves for Heads #2–8 are vertically shifted in 2% increments to reduce overlap.

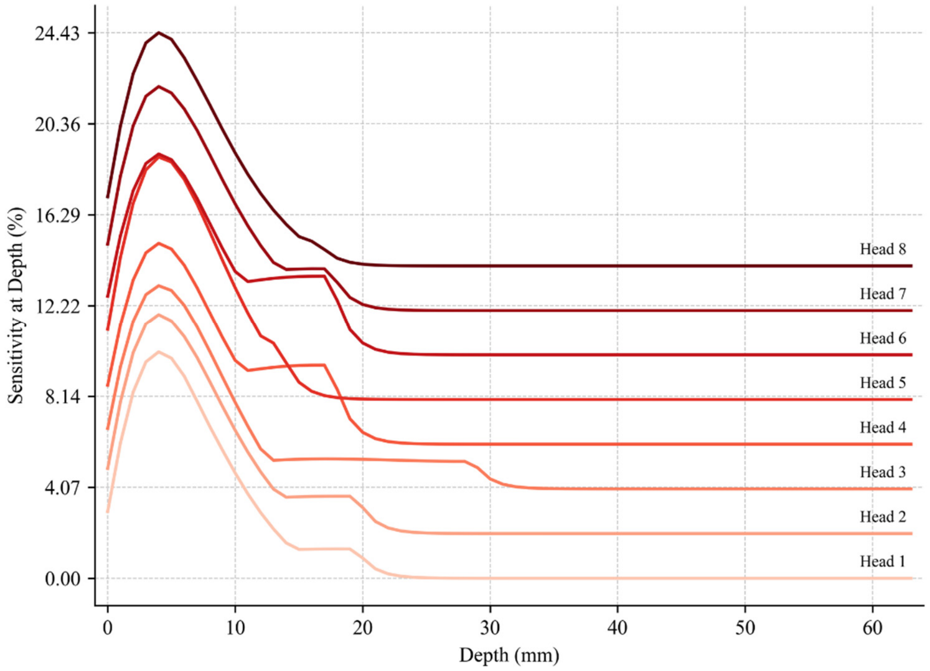


**Figure S97** Sensitivity at depth vs. depth 2D plots for all cadaveric heads at 35 mm source-detector separation. Curves correspond to Heads #1–8, shown from lightest to darkest red. For clarity, curves for Heads #2–8 are vertically shifted in 2% increments to reduce overlap.

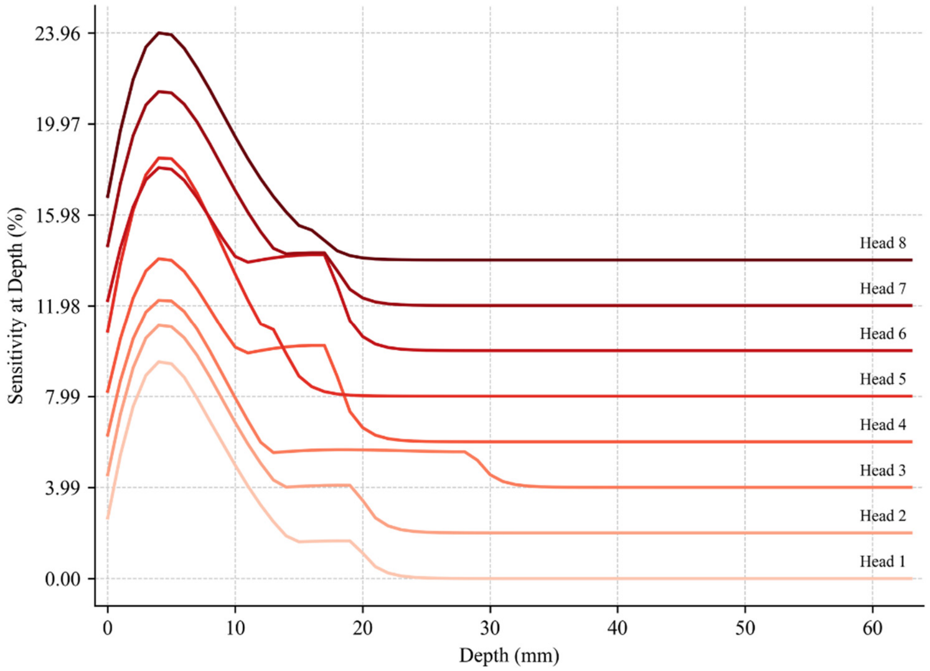


**Figure S98** Sensitivity at depth vs. depth 2D plots for all cadaveric heads at 37 mm source-detector separation. Curves correspond to Heads #1–8, shown from lightest to darkest red. For clarity, curves for Heads #2–8 are vertically shifted in 2% increments to reduce overlap.

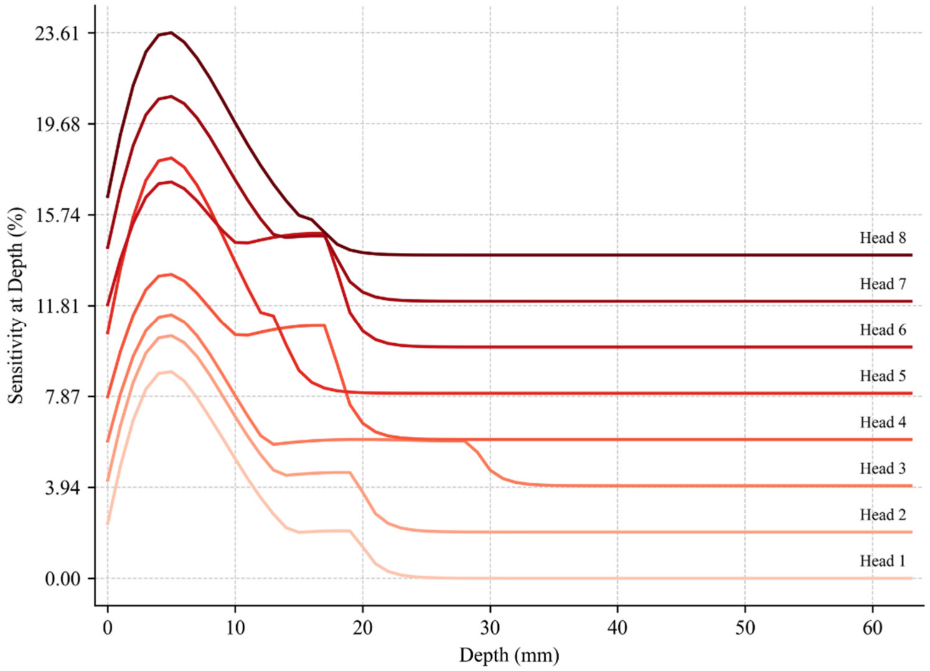


**Figure S99** Sensitivity at depth vs. depth 2D plots for all cadaveric heads at 39 mm source-detector separation. Curves correspond to Heads #1–8, shown from lightest to darkest red. For clarity, curves for Heads #2–8 are vertically shifted in 2% increments to reduce overlap.

### C. Sensitivity at Depth vs. Source-Detector Separation (Box Plots)

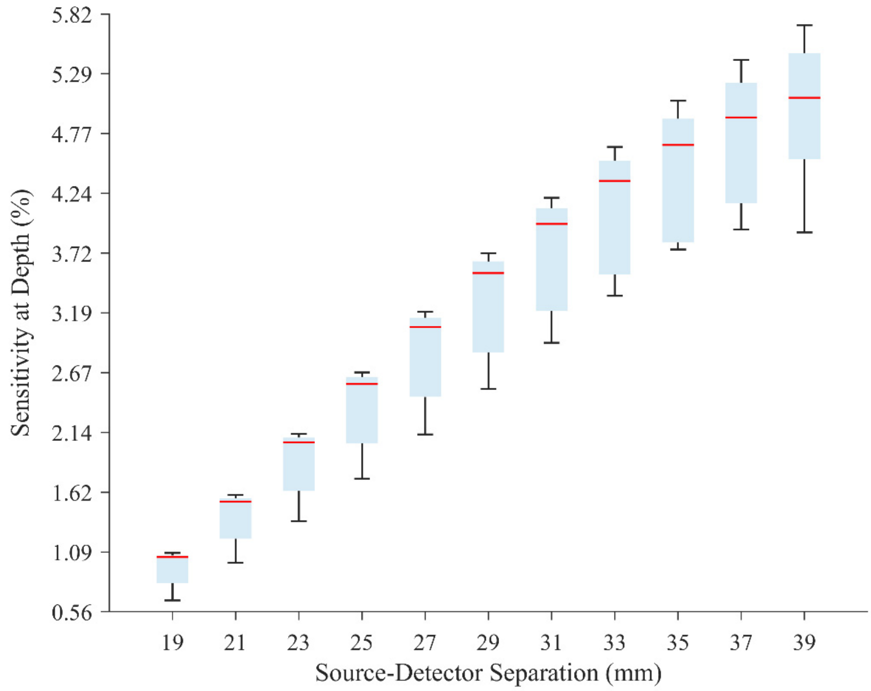


**Figure S100** Sensitivity at depth vs. source-detector separation box plots at 10 mm depth. (Each box plot is generated over all cadaveric heads).

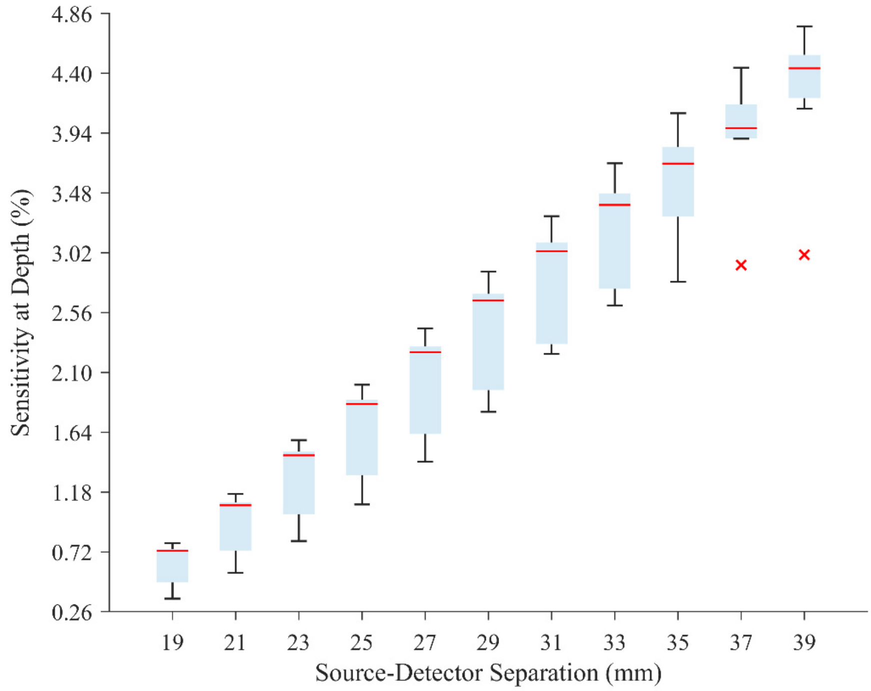


**Figure S101** Sensitivity at depth vs. source-detector separation box plots at 11 mm depth. (Each box plot is generated over all cadaveric heads).

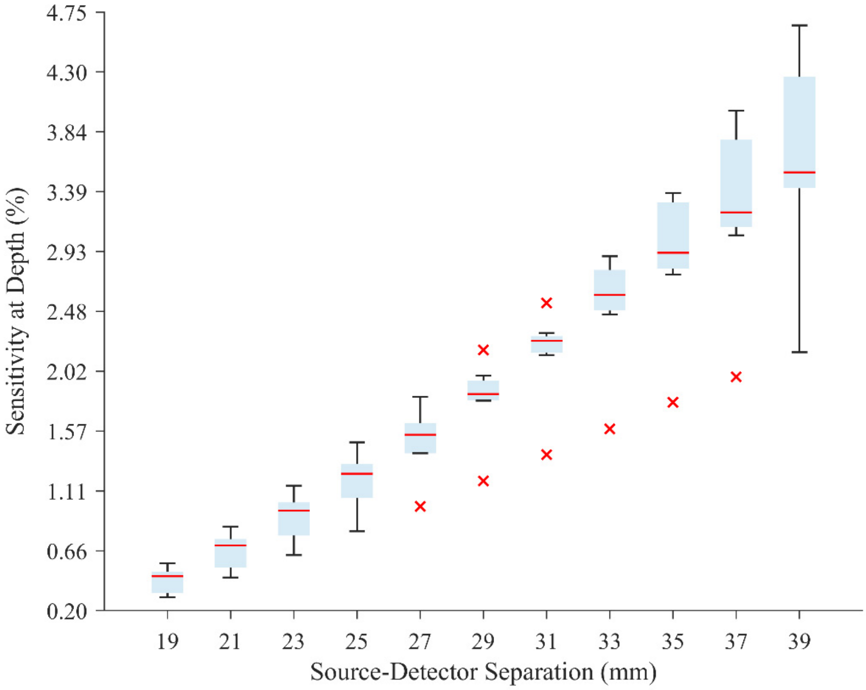


**Figure S102** Sensitivity at depth vs. source-detector separation box plots at 12 mm depth. (Each box plot is generated over all cadaveric heads).

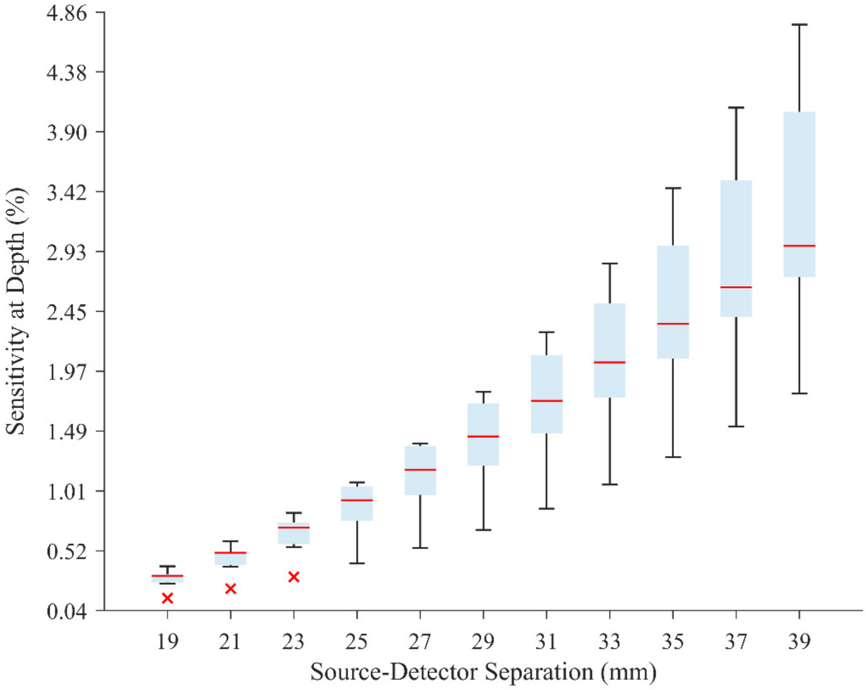


**Figure S103** Sensitivity at depth vs. source-detector separation box plots at 13 mm depth. (Each box plot is generated over all cadaveric heads).

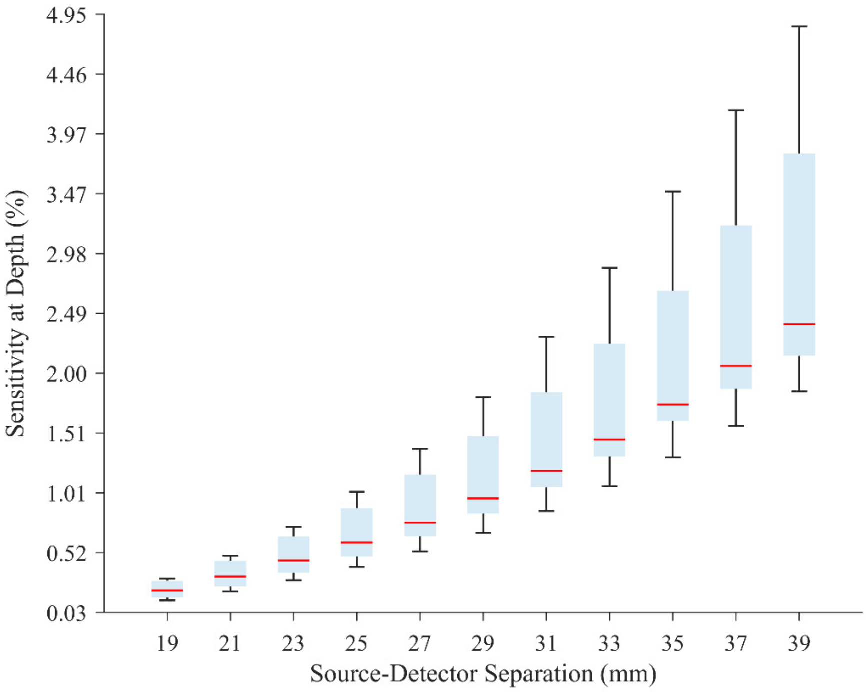


**Figure S104** Sensitivity at depth vs. source-detector separation box plots at 14 mm depth. (Each box plot is generated over all cadaveric heads).

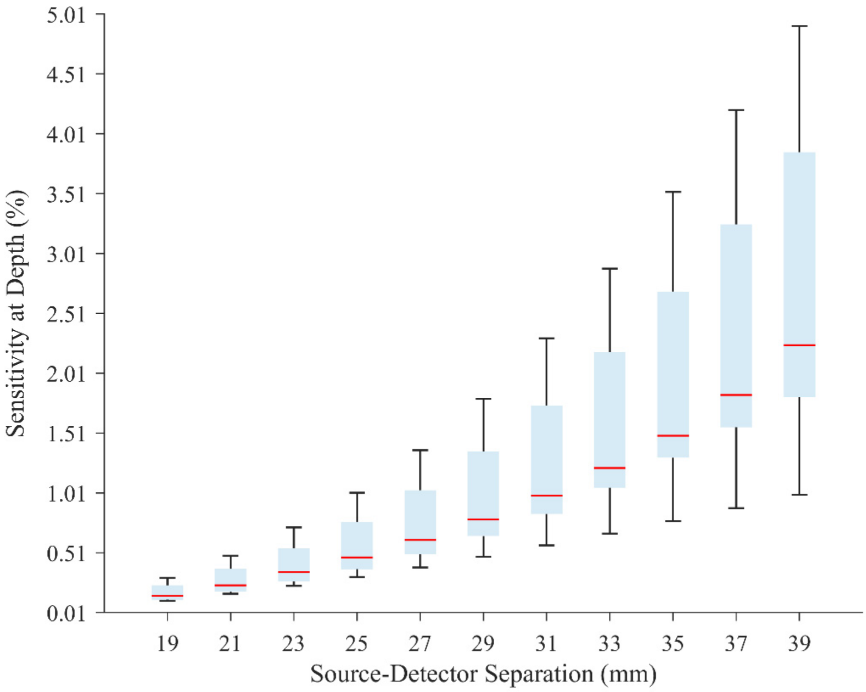


**Figure S105** Sensitivity at depth vs. source-detector separation box plots at 15 mm depth. (Each box plot is generated over all cadaveric heads).

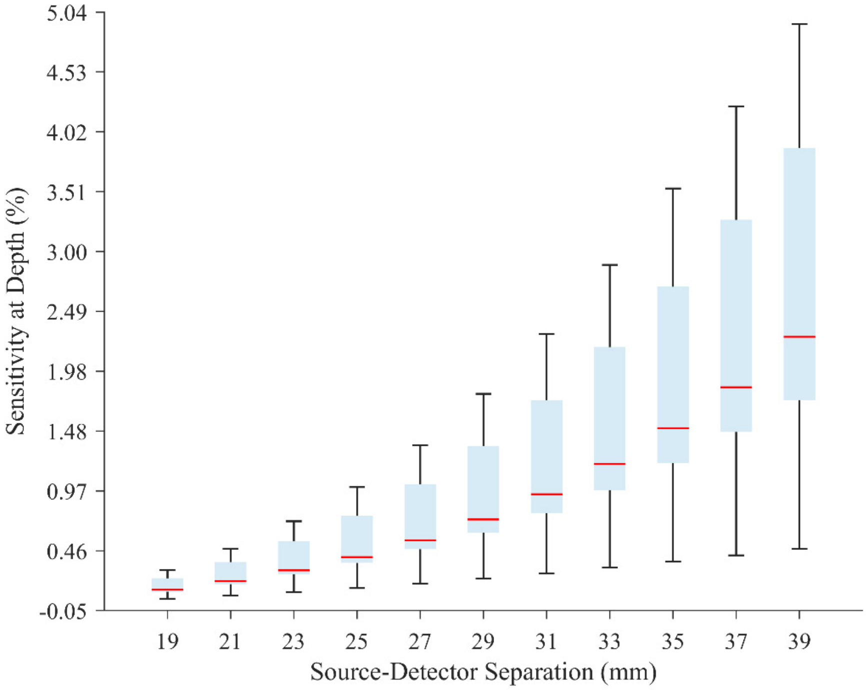


**Figure S106** Sensitivity at depth vs. source-detector separation box plots at 16 mm depth. (Each box plot is generated over all cadaveric heads).

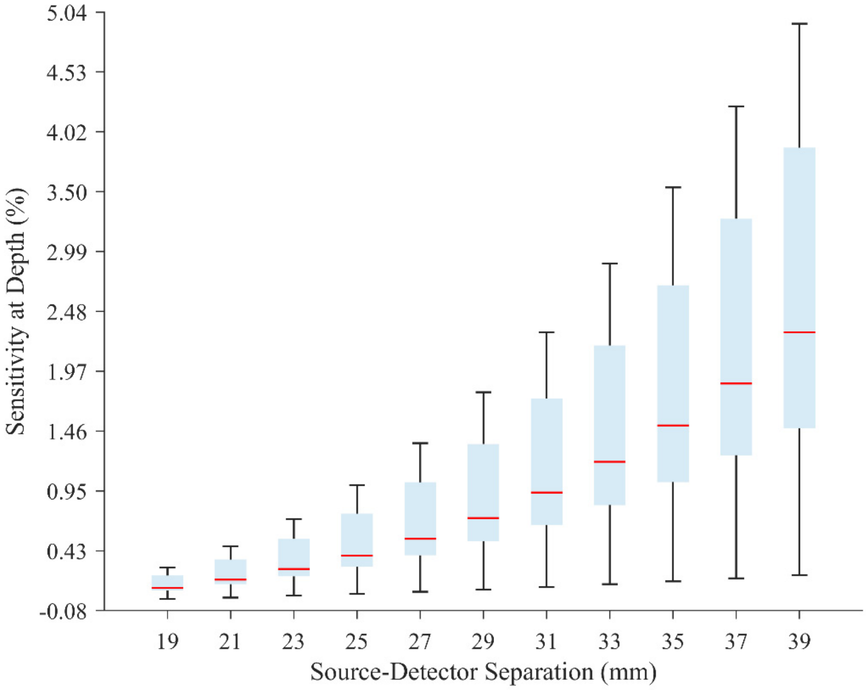


**Figure S107** Sensitivity at depth vs. source-detector separation box plots at 17 mm depth. (Each box plot is generated over all cadaveric heads).

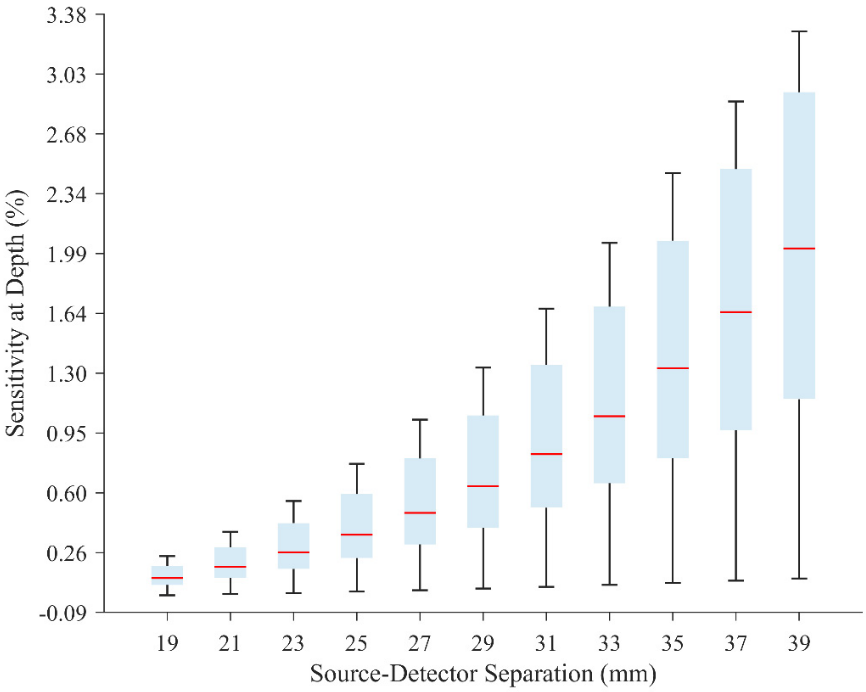


**Figure S108** Sensitivity at depth vs. source-detector separation box plots at 18 mm depth. (Each box plot is generated over all cadaveric heads).

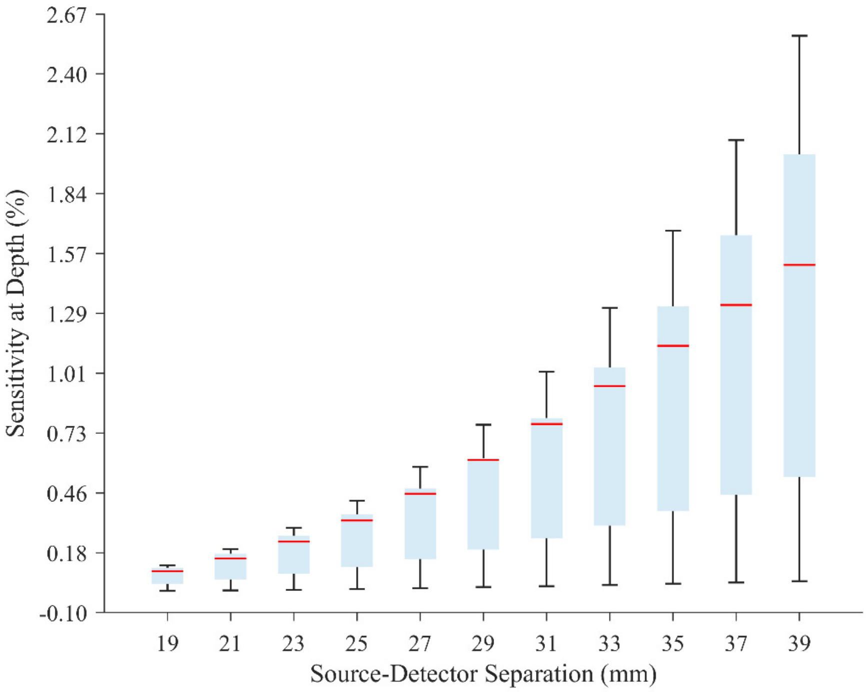


**Figure S109** Sensitivity at depth vs. source-detector separation box plots at 19 mm depth. (Each box plot is generated over all cadaveric heads).

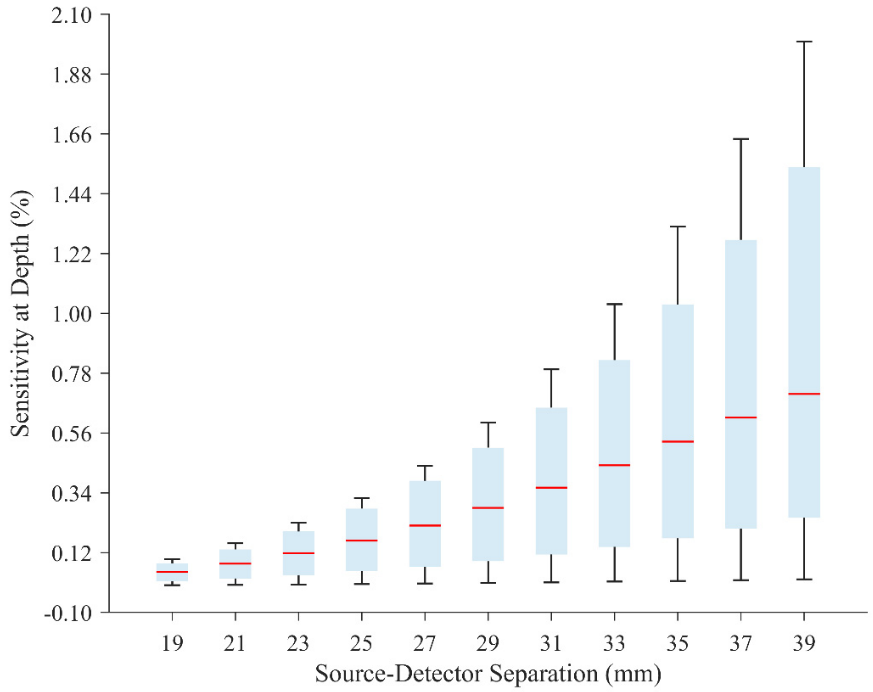


**Figure S110** Sensitivity at depth vs. source-detector separation box plots at 20 mm depth. (Each box plot is generated over all cadaveric heads).

## D. Photon Count vs. Path Length (Histograms)

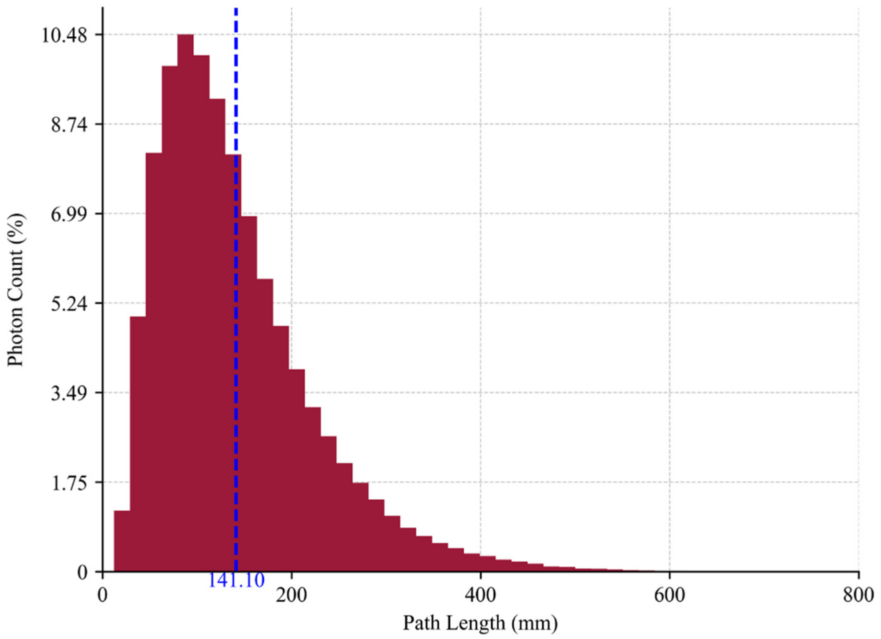


**Figure S111** Photon count vs. path length histogram at 19 mm source-detector separation. Each histogram bin is generated over mean across all cadaveric heads for that data range. Vertical blue dashed line shows mean across all data.

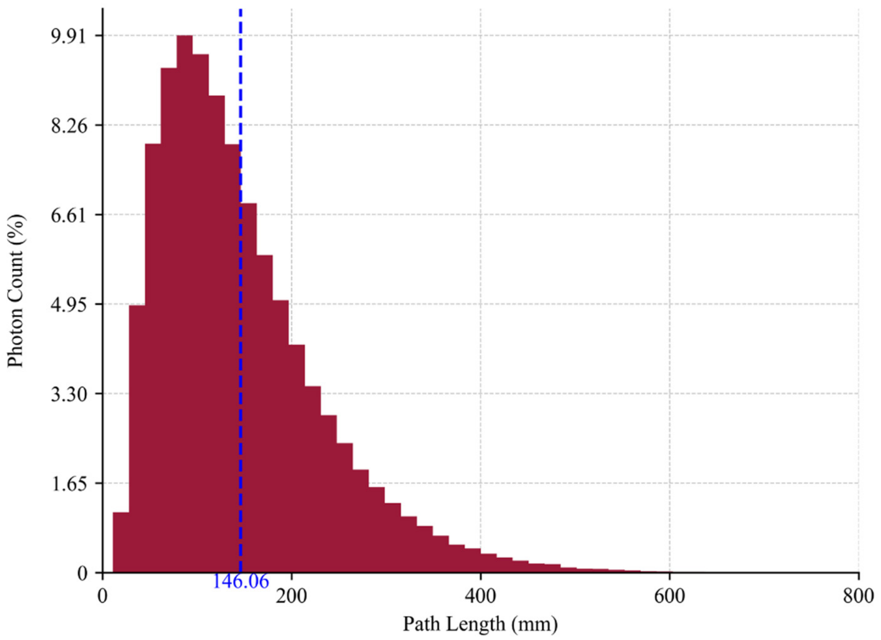


**Figure S112** Photon count vs. path length histogram at 21 mm source-detector separation. Each histogram bin is generated over mean across all cadaveric heads for that data range. Vertical blue dashed line shows mean across all data.

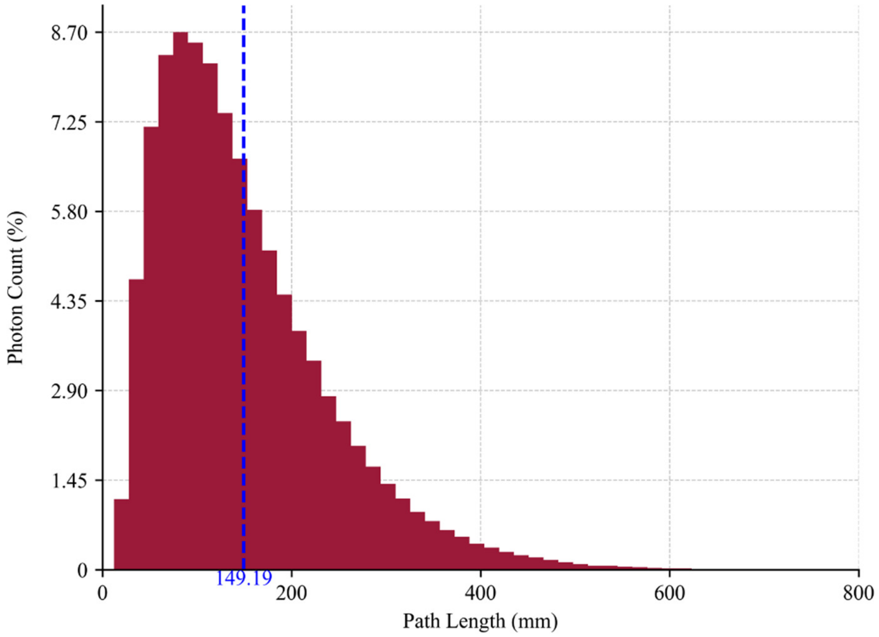


**Figure S113** Photon count vs. path length histogram at 23 mm source-detector separation. Each histogram bin is generated over mean across all cadaveric heads for that data range. Vertical blue dashed line shows mean across all data.

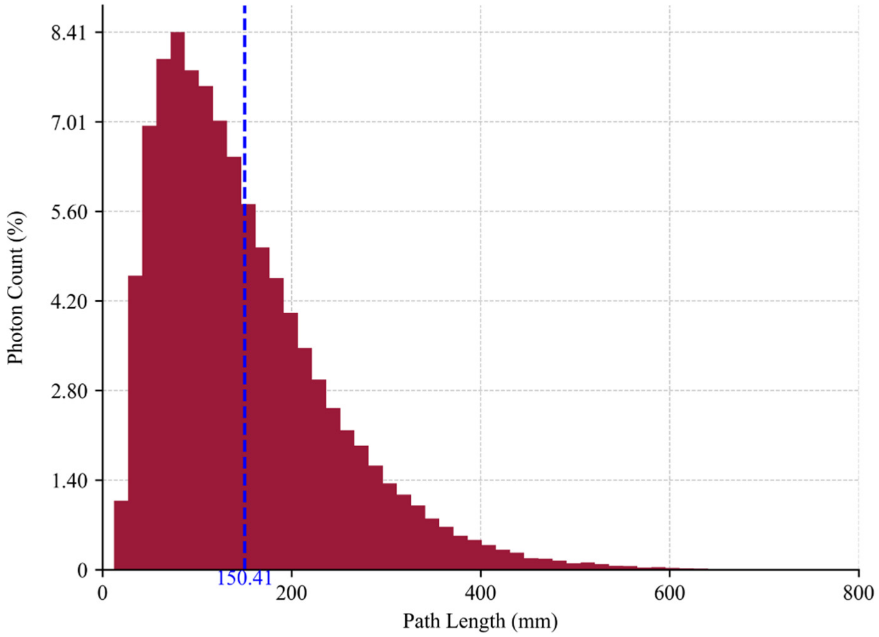


**Figure S114** Photon count vs. path length histogram at 25 mm source-detector separation. Each histogram bin is generated over mean across all cadaveric heads for that data range. Vertical blue dashed line shows mean across all data.

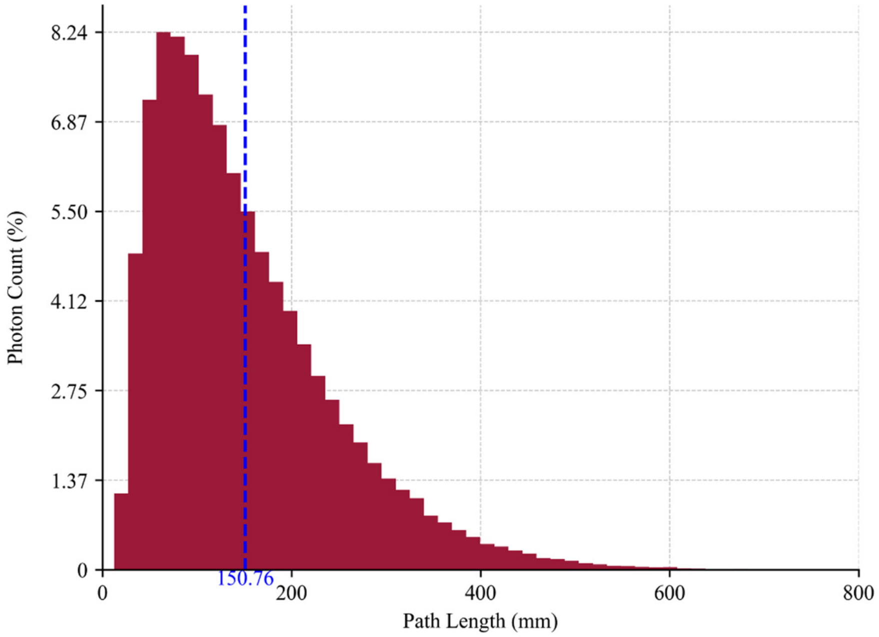


**Figure S115** Photon count vs. path length histogram at 27 mm source-detector separation. Each histogram bin is generated over mean across all cadaveric heads for that data range. Vertical blue dashed line shows mean across all data.

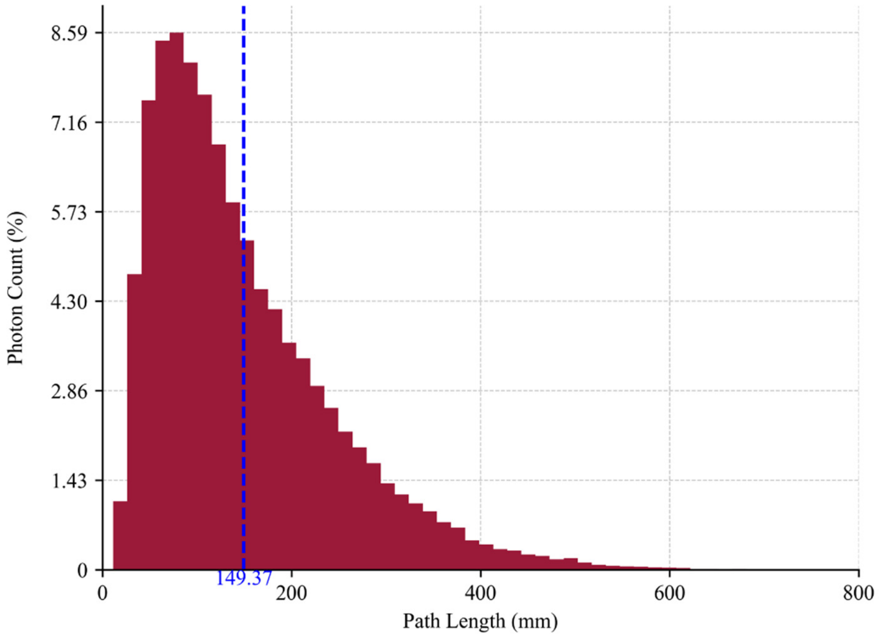


**Figure S116** Photon count vs. path length histogram at 29 mm source-detector separation. Each histogram bin is generated over mean across all cadaveric heads for that data range. Vertical blue dashed line shows mean across all data.

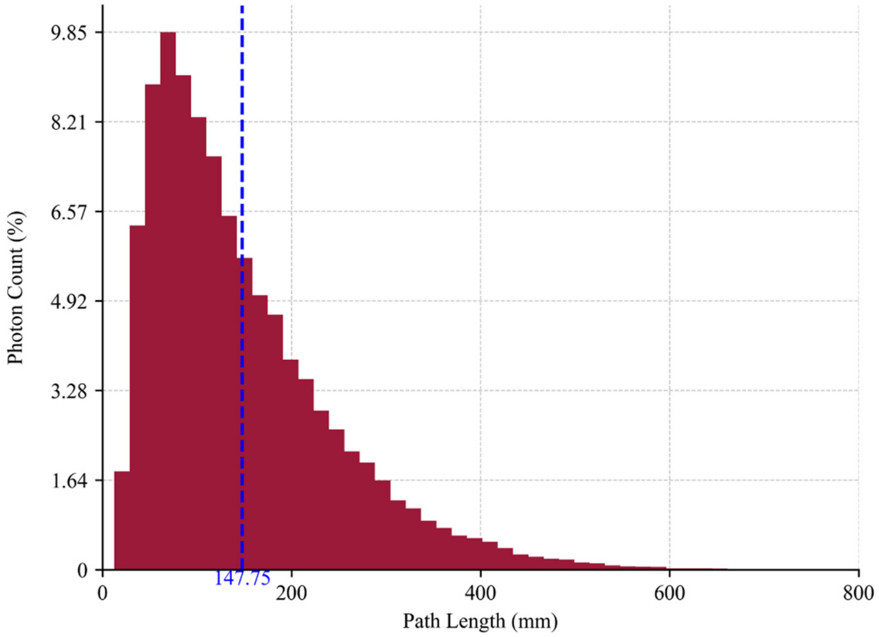


**Figure S117** Photon count vs. path length histogram at 31 mm source-detector separation. Each histogram bin is generated over mean across all cadaveric heads for that data range. Vertical blue dashed line shows mean across all data.

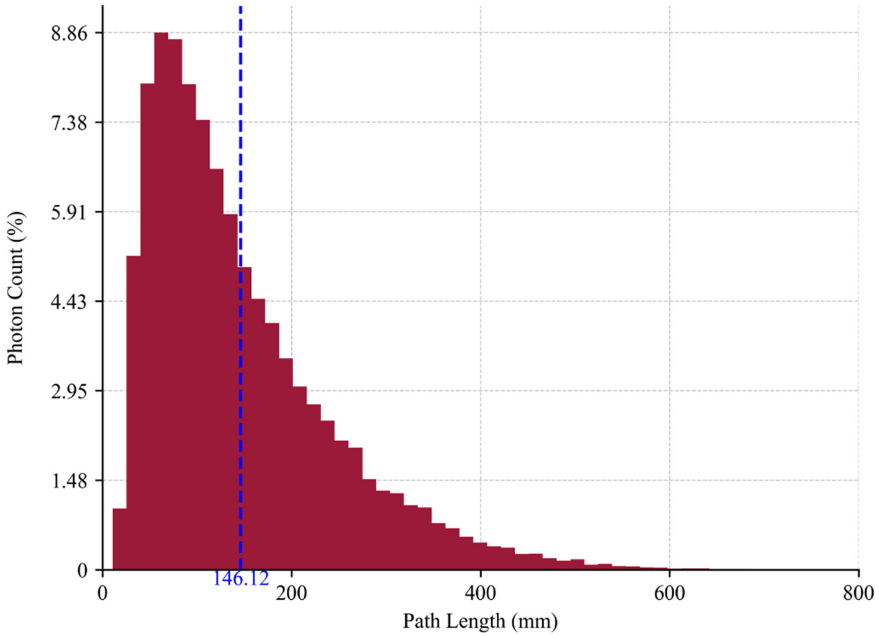


**Figure S118** Photon count vs. path length histogram at 33 mm source-detector separation. Each histogram bin is generated over mean across all cadaveric heads for that data range. Vertical blue dashed line shows mean across all data.

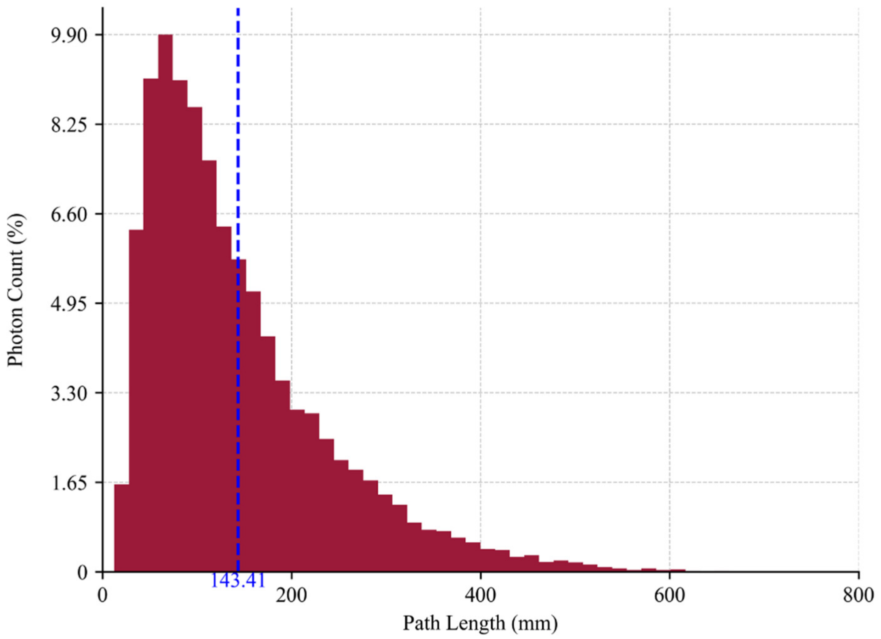


**Figure S119** Photon count vs. path length histogram at 35 mm source-detector separation. Each histogram bin is generated over mean across all cadaveric heads for that data range. Vertical blue dashed line shows mean across all data.

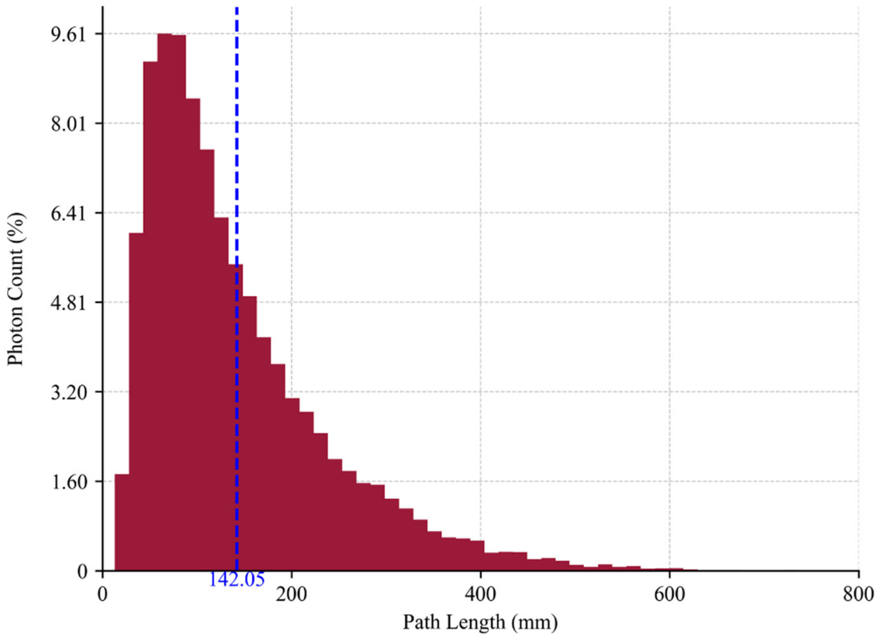


**Figure S120** Photon count vs. path length histogram at 37 mm source-detector separation. Each histogram bin is generated over mean across all cadaveric heads for that data range. Vertical blue dashed line shows mean across all data.

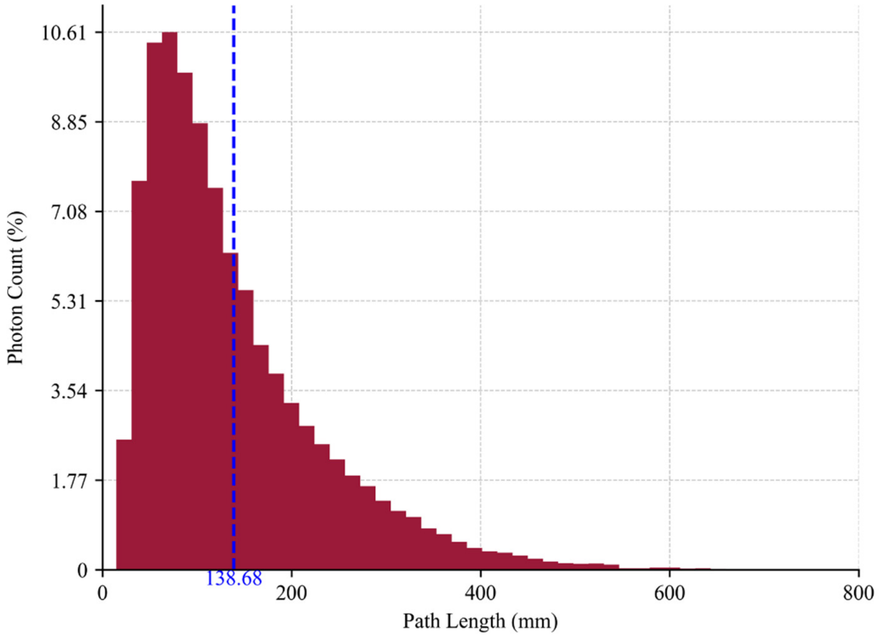


**Figure S121** Photon count vs. path length histogram at 39 mm source-detector separation. Each histogram bin is generated over mean across all cadaveric heads for that data range. Vertical blue dashed line shows mean across all data.
